# Dinuclear Nickel Complexes Containing Imidazole-Based Bis(iminophenolate) Derivatives: Highly Active Catalysts for Ring-Opening Copolymerization of Carbon Dioxide with Alicyclic Epoxides

**DOI:** 10.1021/acs.inorgchem.6c02993

**Published:** 2026-09-01

**Authors:** Jun-Cheng Chen, Guan-Lin Liu, Bao-Tsan Ko

**Affiliations:** Department of Chemistry, 34916 National Chung Hsing University, Taichung 402202, Taiwan

## Abstract

We report the synthesis of a series of dinuclear nickel complexes based on triphenylimidazole-derived salen-type ligands bearing different substituents on the phenyl rings. These complexes were applied as single-component catalysts for the copolymerization of CO_2_ and alicyclic epoxides. All dinuclear Ni complexes exhibited high catalytic activity and excellent selectivity for the alternating copolymerization of CO_2_ and cyclohexene oxide (CHO). Among them, the methoxy-substituted bis­(acetate) and dichloride complexes **2** and **6** showed the outstanding performance, achieving turnover frequencies (TOFs) exceeding 2000 h^–1^ and turnover numbers (TONs) over 9000. Notably, the dichloride complex **6** also enabled terpolymerization of different alicyclic epoxides, affording CO_2_-based polycarbonates bearing vinyl or acrylate functionalities. Kinetic analysis suggests that the copolymerization follows first-order dependence on both complex **6** and CHO concentrations.

## Graphical abstract

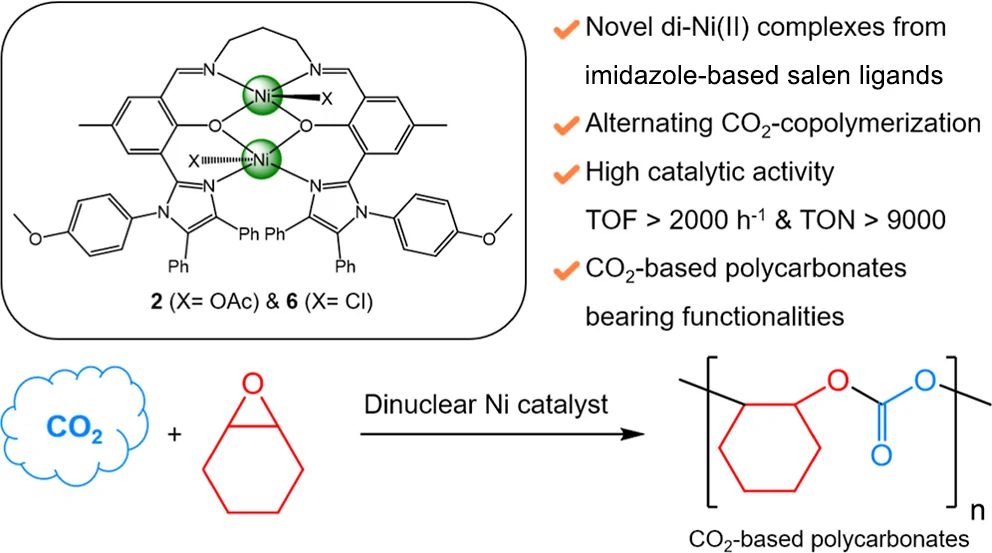

## Introduction

The ring-opening copolymerization (ROCOP) of carbon dioxide (CO_2_) with epoxides to produce polycarbonates is considered a promising strategy for CO_2_ utilization and fixation.^1–5^ Pioneering studies by the group of Inoue have inspired extensive research into the catalyst development for this kind of copolymerization, in which metal complexes have been shown to play a key role.^6,7^ Within these metal complex-based catalysts, various complex families have been reported,^8–12^ among which salen-based chromium­(III) and cobalt­(III) complexes have demonstrated favorable catalytic properties in prior work, attracting considerable attention.^13–17^ As a result, salen-derived metal catalyst systems have been widely developed, with distinct structural modifications to enhance the catalytic performance. For example, the incorporation of nucleophilic groups such as quaternary ammonium halides or sterically hindered organic bases into the salen-type ligand frameworks enabled the development of bifunctional catalysts with significantly improved performances.^18–21^ In addition, motivated by the high efficiency of β-diiminate zinc catalysts,^22–27^ considerable efforts have been devoted to the design and development of dinuclear or multinuclear salen-based systems.^28–36^ For instance, Lu and co-workers have reported a new class of ligands featuring two salen-type Al­(III) centers, with quaternary ammonium groups and multiple chiral centers introduced into the ligand framework. This bifunctional dinuclear Al­(III) catalyst exhibited excellent performance in the asymmetric copolymerization of epoxides with cyclic anhydrides or CO_2_, affording copolymers with high enantioselectivity.^37,38^ It was noted that the group of Williams has developed a series of multidentate ligands for the preparation of bimetallic complexes.^9^ They have systematically investigated homo- and heterodinuclear catalysts based on reduced Robson-type ligands for the copolymerization of CO_2_ with cyclohexene oxide (CHO).^39–41^ Among them, the Mg­(II)/Co­(II) complex exhibited excellent catalytic activity, with a turnover frequency (TOF) exceeding 12,000 h^–1^.^41^ Furthermore, the introduction of crown ether-like structures into the salen-based framework enabled the construction of a series of heterodinuclear or trinuclear complexes based on differences in the coordination environment of the designed ligands.^42–49^ Accordingly, a family of Co­(III)/Na­(I) or Co­(III)/K­(I) complexes have been reported as single-component catalysts for the copolymerization of CO_2_ with propylene oxide, showing the notable performance. In particular, the Co­(III)/Na­(I) catalyst exhibited a TOF of 1428 h^–1^ at 70 °C, along with high selectivity toward poly­(propylene carbonate).^47^ Moreover, Mashima et al. reported a macrocyclic ligand system in which three salen-type units were assembled into a crown ether-like framework.^50–52^ The resulting multimetallic Co_3_(II)/Nd­(III) complex showed good thermal tolerance and excellent activity in CO_2_/CHO copolymerization, with a TOF of 1625 h^–1^ and a turnover number (TON) of up to 13,000.^51^

Our group has focused on developing dinuclear metal complexes as homogeneous catalysts for the ROCOP of epoxides with CO_2_ or cyclic anhydride to afford polycarbonates or polyesters. In our earlier studies, we reported an array of dinuclear metal complexes based on benzotriazole-derived bisphenolate ligand systems. The effects of the central metal,^53–55^ the diimine or diamine backbone of the ligands,^56–59^ and the characteristics of the auxiliary ligands on catalytic performance were deeply investigated.^60–65^ In addition, the scope of nitrogen-containing heterocycles in the ligand framework was expanded, including benzothiazole, pyrazole, imidazole, and imidazopyridine motifs.^66–72^ These structurally well-defined bimetallic complexes were demonstrated to be efficient catalysts for such catalytic epoxide copolymerization. Based on these previous studies, tuning the auxiliary ligands coordinated to the metal centers, as well as the nitrogen-containing heterocycles in the ligand framework, can significantly influence the copolymerization activity. As depicted in Scheme 1, representative catalyst systems exhibited high catalytic performances, reaching TOF values of up to 688 h^–1^ for the dinickel dichloride complex **I** based on a benzotriazole-containing salen-type ligand and 816 h^–1^ for the dinickel diacetate complex **II** containing a triphenylimidazole-based diamine-bisphenolate ligand, respectively.^61,69^ However, the effect of substituent groups on the ligand framework itself has been less explored. Therefore, in this work, triphenylimidazole-derived phenolate compounds were synthesized from various aniline derivatives through an effective one-pot multicomponent modular approach, allowing the introduction of substituents with different electronic properties on the N-containing heterocycles. These compounds were further used to construct a series of salen-like dinickel complexes bearing either bis­(acetate) or dichloride ancillary ligands, which were evaluated as one-component catalysts for the ROCOP of CO_2_ with alicyclic epoxides. In addition, the kinetics of CO_2_/CHO copolymerization was investigated using the most active dichloride dinickel complex as the catalyst in this contribution.

**Scheme 1:**
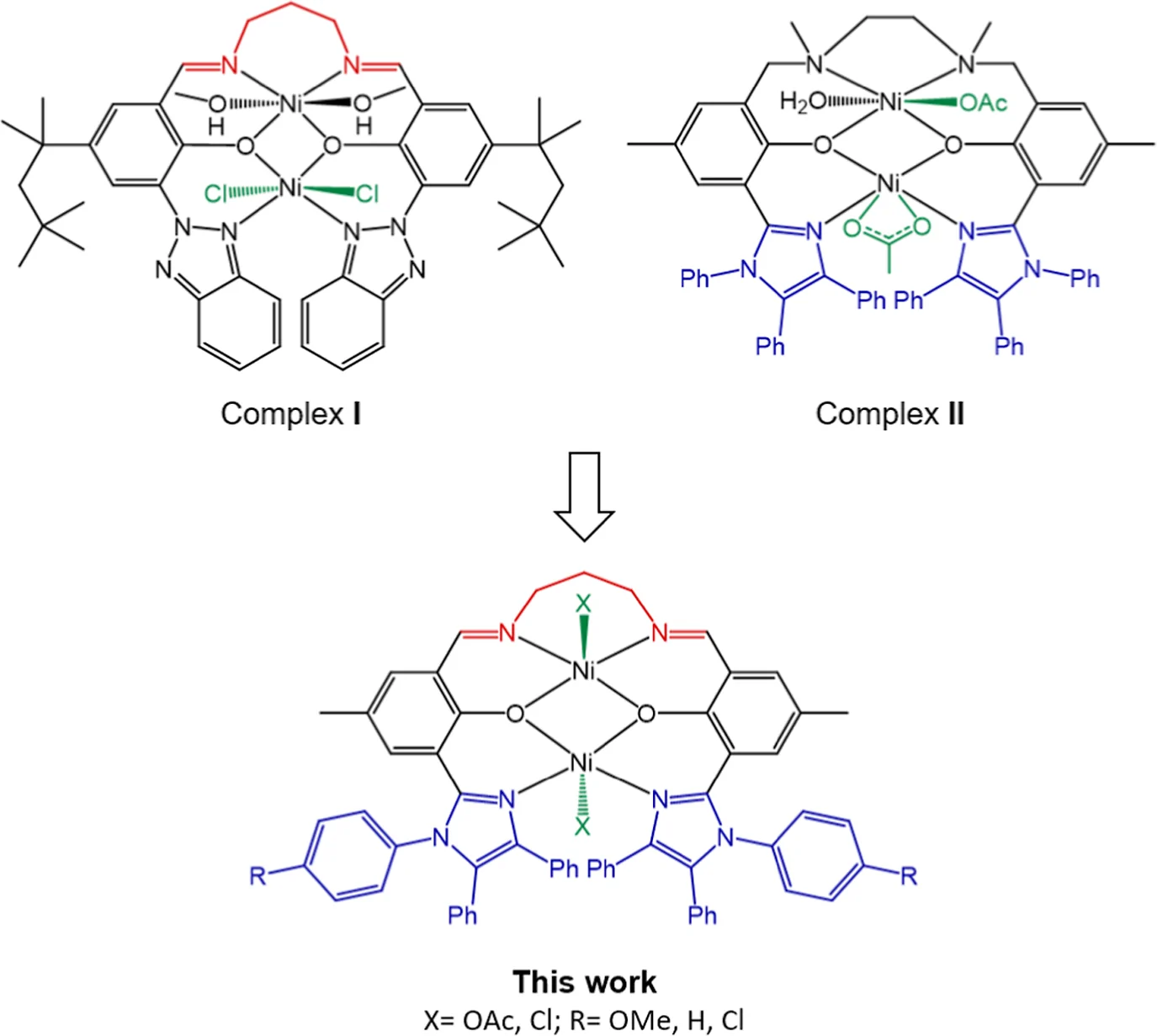
Inspiration and Development for Dinickel Complexes of Nitrogen-Containing Heterocyclic Phenolate Ligands Used for CO_2_/Epoxide Copolymerization in This Study

## Results and Discussion

### Synthesis and Crystal Structure of Dinickel­(II) Complexes

According to our previous reports,^69,71^ a series of triphenylimidazole-derived phenolate compounds, ^*H*^TPImP-H, ^*OMe*^TPImP-H, and ^*Cl*^TPImP-H, were prepared from aniline derivatives containing different functional groups. Subsequent Duff formylation and imination reaction afforded the imidazole-containing salen-like hexadentate ligand precursors, ^*H3C*^L-H_2_, ^*OMe3C*^L-H_2_, ^*OMe4C*^L-H_2_, and ^*Cl3C*^L-H_2_ bearing various substituents, and their synthetic routes are illustrated in Scheme S1. Further reactions with 2 equiv of nickel­(II) acetate tetrahydrate or nickel­(II) chloride hexahydrate in the presence of excess triethylamine (NEt_3_) in methanol afforded the dinuclear nickel complexes **1**–**7** incorporated with two acetate ([LNi_2_(CH_3_COO)_2_], **1**–**4**) or two chloro ([LNi_2_Cl_2_], **5**–**7**) auxiliary coligands, as presented in Scheme 2. The above dinickel complexes were fully characterized by elemental analysis and mass spectrometry. In addition, the molecular structures of several complexes were further confirmed by single-crystal X-ray crystallography (Figures 1, 2, S1 and S2). The selected bond lengths and bond angles between the nickel ions and ligands in these solid-state structures are summarized in Table S2.

**Scheme 2:**
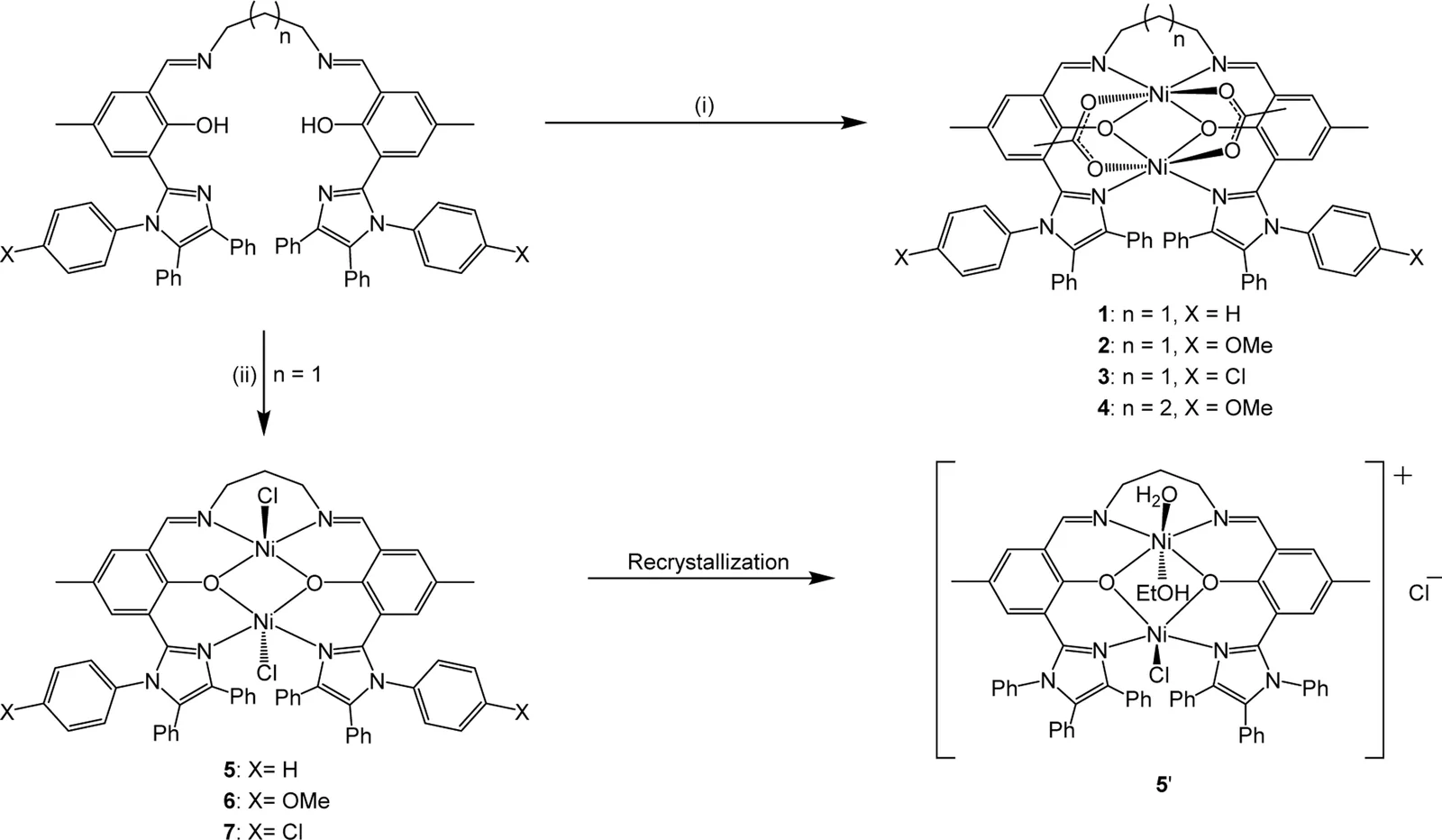
Synthetic Pathways of Dinuclear Nickel Complexes **1–7** ^a^

a Reagents and conditions: (i) Ni­(OAc)_2_.4H_2_O (2.0 equiv)/NEt_3_ (5.0 equiv), MeOH, reflux, 24 h, 70% for **1**, 86% for **2**, 77% for **3** and 75% for **4**. (ii) NiCl_2_·6H_2_O (2.0 equiv)/NEt_3_ (5.0 equiv), MeOH, reflux, 24 h, 88% for **5**, 86% for **6** and 82% for **7**.

**Figure 1:**
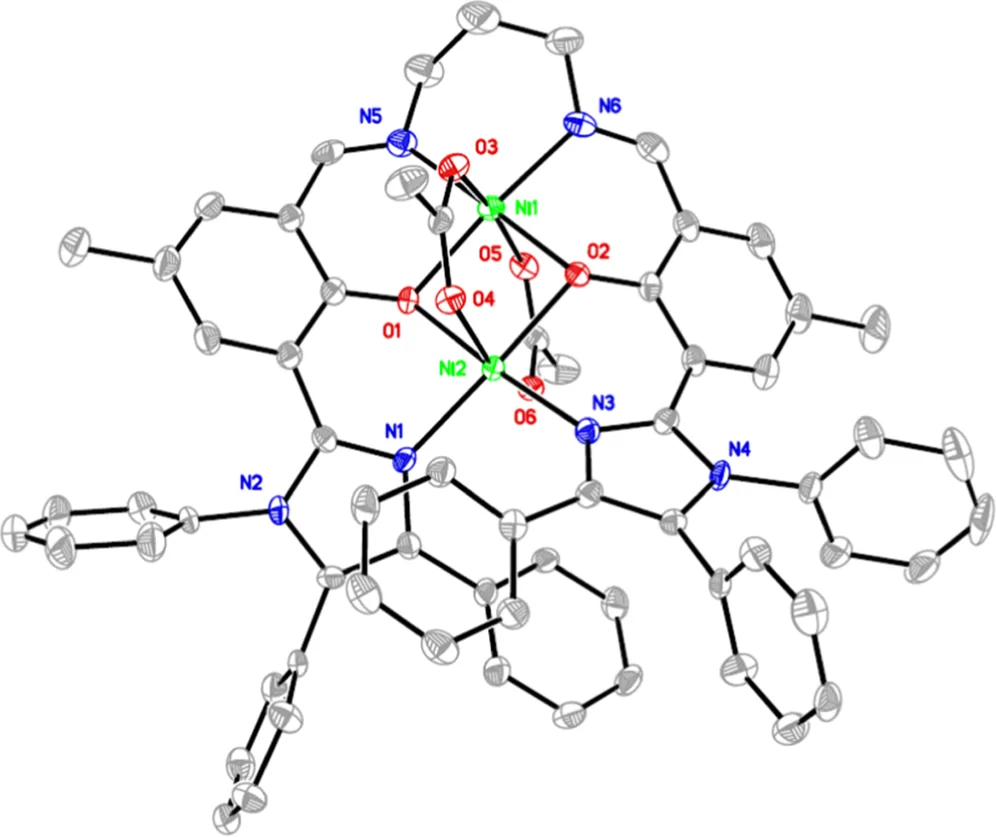
ORTEP drawing of complex **1** (50% probability level).

**Figure 2:**
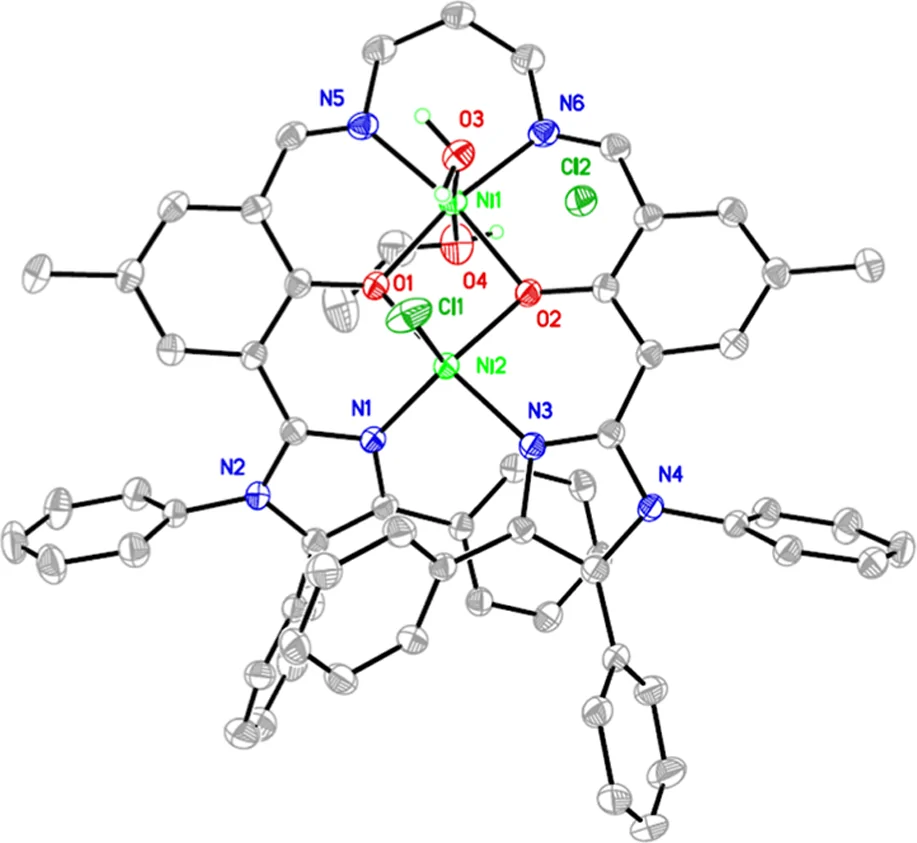
Solid-state molecular structure of complex **5′** (50% probability level).

In the crystal structures, except for the different coordinated coligands, all complexes revealed dinuclear nickel species chelated by the hexadentate ligands derived from triphenylimidazole-phenolate derivatives. Complex **1** is described as a representative bis­(acetate) dinickel complex in the following discussion. The ORTEP drawing of **1** is shown in Figure 1. This structure features a dinuclear nickel complex chelated by a salen-type hexadentate imidazole-based ligand together with two bidentate bridging acetate coligands, and this complex is structurally analogous to the bis­(acetate) dinickel salen-like complexes previously reported by our group.^56,59,66^ In complex **1**, two phenolate oxygen atoms of the salen-type ligand bridge to two nickel centers. Both nickel ions are chelated by the ligand framework in a N_2_O_2_ coordination environment; however, slight differences are observed in their coordination environments. The Ni(1) center is coordinated by two nitrogen atoms from the propylene-bridged imine moiety, whereas Ni(2) is chelated by the two nitrogen donors of the triphenylimidazole group. The coordination geometries of dinickel centers are both distorted octahedral configurations, evidenced from the axial bond angles, O(3)–Ni(1)–O(5) = 169.08(16)° and O(4)–Ni(2)–O(6) = 158.22(16)°, respectively. Notably, for the bis­(acetate) dinickel complex **1** reported herein (Figure 3a), the average Ni–N­(imidazole) bond length (∼2.07 Å) is slightly longer than the average Ni–N­(imine) bond length (∼2.04 Å), indicating a weaker coordination interaction between Ni­(II) and N-donor of the imidazole moiety. This may be attributed to the steric hindrance arising from the three phenyl substituents on the imidazole ring, which weakens the metal–ligand interaction. As a consequence, electron-donating or electron-withdrawing substituents on the phenyl ring exert no pronounced influence on the Ni–N­(imidazole) bond length. In addition, similar steric effects are reflected in the O­(acetate)–Ni–O­(acetate) bond lengths and angles. The average Ni(2)–O­(acetate) bond distances (2.10–2.13 Å) are slightly longer than the corresponding Ni(1)–O­(acetate) bond distances (∼2.07 Å), whereas the O–Ni(2)–O angles (156°–159°) are smaller than the O–Ni(1)–O angles (169°–171°). These structural features further highlight how the steric bulkiness of the triphenylimidazole moiety influences the coordination environment around the nickel centers.

**Figure 3:**
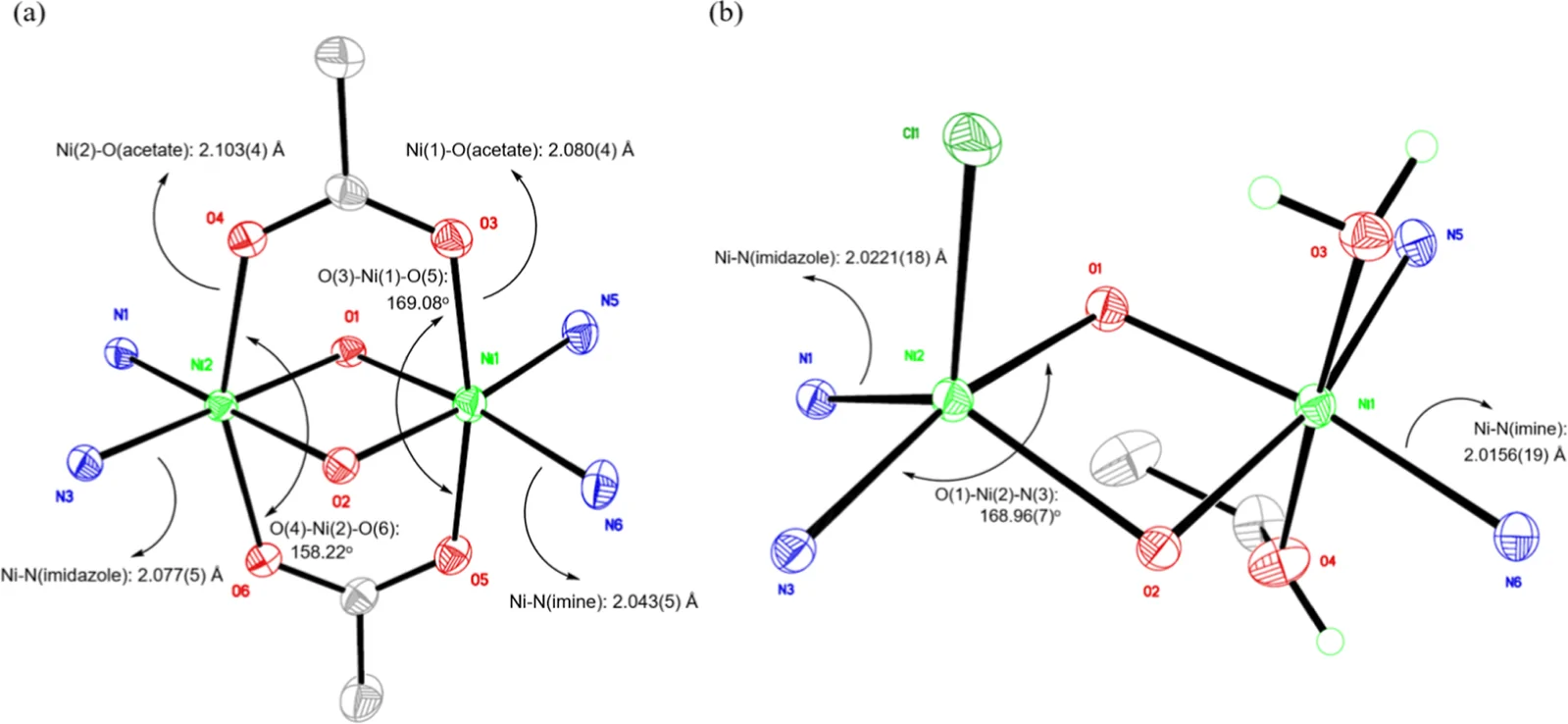
Bonding modes of auxiliary coligands and nickel ions of (a) complex **1** and (b) complex **5′**.

The molecular structure of complex **5** was initially inferred to be a neutral dichloride dinuclear nickel species based on elemental analysis and mass spectrometric characterization (Figure S3). Single crystals suitable for X-ray diffraction were obtained from a mixed solvent system of ethanol and diethyl ether; however, an unexpected molecular structure, denoted as **5′**, was isolated. The solid-state structure of **5′** is shown in the ORTEP diagram (Figure 2). This crystal structure is distinct from the halide-coordinated dinickel structures reported in our previous studies.^61,65,70^ The imidazole-based salen-type ligand in complex **5′** chelates two nickel centers with a coordination mode identical to that observed in the bis­(acetate) dinickel complexes described above. The Ni(2) center is coordinated by one chloride and adopts a distorted trigonal bipyramidal geometry, with a τ_5_ value of 0.71, in which the O(1)–Ni(2)–N(3) axis defines the principal coordination axis. In contrast, the anticipated chloride does not bind to the Ni(1) atom; instead, ethanol and water molecules occupy trans positions on Ni(1), resulting in a distorted octahedral coordination geometry (Figure 3b). The uncoordinated chloride anion acts as a counterion to maintain the overall charge neutrality of complex **5′**.

The separation between two nickel ions in this work is approximately 2.80–3.10 Å, which is close to the Ni–Ni distance (∼3.0 Å) observed in our previously reported bimetallic nickel complexes supported by benzotriazole-based salen-like ligands.^56,59,61,65,66^ As indicated by the solid-state structures of the aforementioned complexes, the designed imidazole-containing salen-type ligands provide a N_4_O_2_-hexadentate coordination environment, which coordinates with nickel acetate or nickel chloride to form bimetallic complexes. Such dinickel complex structures hold promise for catalyzing the ROCOP of CO_2_ and alicyclic epoxides. Notably, in complex **5′**, ethanol and water molecules are coordinated to the Ni(1) atom, which may serve as an active site for effective substrate activation during CO_2_/epoxide copolymerization.

### Copolymerization of Carbon Dioxide (CO_2_) with Alicyclic Epoxides by Dinickel­(II) Complexes

We explored the performances of this series of dinuclear nickel complexes **1**–**7** without the addition of cocatalysts for the copolymerization of CO_2_ with cyclohexene oxide (CHO), and the catalytic data are summarized in Table 1. First, the copolymerization of CO_2_ and CHO was carried out under the conditions of [CHO]_0_/[catalyst]_0_ = 3200/1, at 120 °C, with an initial CO_2_ pressure of 20.7 bar (300 psi) for 2 h. On the basis of the copolymerization results, all Ni complexes as single-component catalysts exhibited good catalytic activities and demonstrated excellent reaction selectivity. No cyclohexene carbonate (CHC) byproducts were observed, and a perfectly alternating CO_2_-based poly­(cyclohexene carbonate) (PCHC) containing >99% carbonate repeat units was obtained. The CO_2_-based polycarbonates obtained from the above copolymerization were characterized by ^1^H NMR spectroscopy. The resulting PCHC exhibited the characteristic signals of perfectly alternating CO_2_ and CHO linkages, with no detectable polyether sequences arising from the homopolymerization of CHO (Figure S4a). Subsequently, the catalytic data from entries 1–3 of Table 1 revealed the influence of substituents in the ligand framework on the copolymerization performance of the dinickel bis­(acetate) catalysts bearing the similar salen-type backbone. Evidently, the functional groups on the phenyl ring of the imidazole moiety had a significant effect on the catalytic activity. The methoxy-substituted complex **2** displayed the highest activity, giving a CHO conversion of 56% and a turnover frequency (TOF) of up to 896 h^–1^, whereas the chloro-substituted analogue **3** showed the lowest activity with a CHO conversion of only 40%. The effect of the substituents became more pronounced in the dichloride dinickel catalysts (Table 1, entries 5–7), in which catalyst **6** exhibited twice the activity of catalyst **7** (48% vs. 24%; Table 1, entries 6 and 7). This phenomenon suggests that, within this series of nickel catalysts, electron-donating groups on the imidazole-phenolate ligands are favorable for enhancing catalytic activity. The electron-donating methoxy-substituent increases the donor ability of the imidazole moiety, thereby enhancing the electron density at the nickel center, whereas the chloro-substituent exerts the opposite effect. Although a more electron-deficient nickel center may facilitate epoxide activation, it may also strengthen the coordination of propagating alkoxide or carbonate intermediates, thereby retarding chain propagation. Therefore, an appropriate electron-donating ability appears to be more favorable for maintaining an efficient catalytic cycle. This finding provides a valuable strategy for the design of ligand frameworks toward high-performance bimetallic catalysts. In addition, the impact of the salen-like backbone on the copolymerization activity was also investigated. The nickel acetate catalyst **4** with a 1,4-butylene diimine bridging backbone exhibited significantly lower activity, affording only a 23% CHO conversion (Table 1, entry 4) under identical conditions. This trend differs from the previous report,^59^ suggesting that different ligand scaffold systems may display distinct preferences toward the salen-type backbone.

**Table 1:**
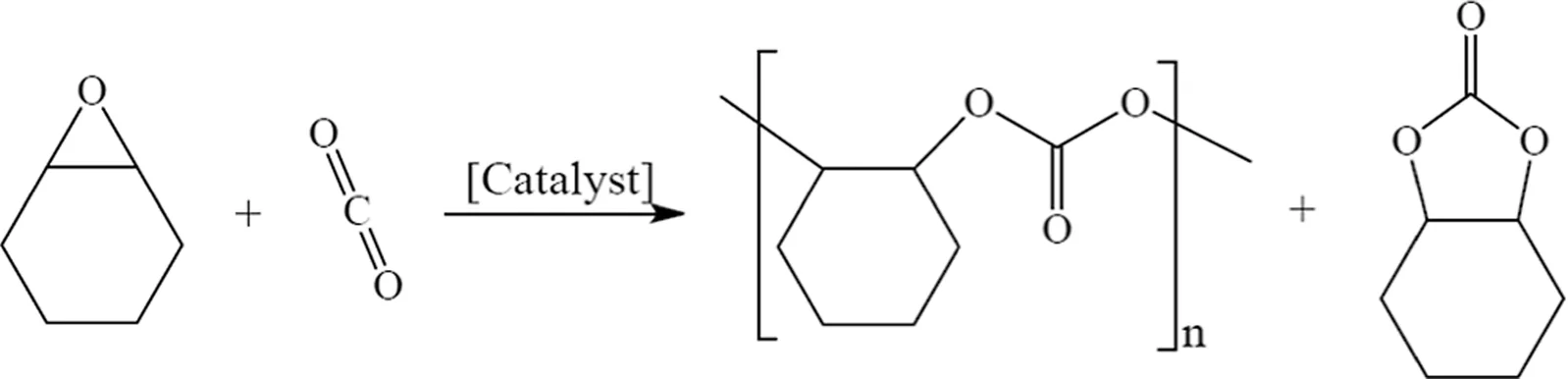
ROCOP of CO_2_ and CHO Mediated by Dinuclear Nickel Complexes **1**–**7** ^a^

entry	catalyst	% CHO conv^b^	% copolymer^b^ (%CO_3_ linkages)^c^	TON^d^	TOF/h^–1^ ^e^	*M*_n_ [D̵]^f^ (g mol^–1^)
1	**1**	47	>99 (>99)	1504	752	20,000 [1.20]
2	**2**	56	>99 (>99)	1792	896	19,000 [1.19]
3	**3**	40	>99 (>99)	1280	640	15,000 [1.19]
4	**4**	23	>99 (>99)	736	368	8400 [1.24]
5	**5**	43	>99 (>99)	1376	688	17,300 [1.19]
6	**6**	48	>99 (>99)	1536	768	18,600 [1.19]
7	**7**	24	>99 (>99)	768	384	11,000 [1.21]

a Copolymerization conditions: 0.03125 mmol of catalyst, 100.0 mmol of CHO, *p*CO_2_^0^ = 20.7 bar (300 psi), 120 °C, 2 h.

b Based on ^1^H NMR analysis of the reaction mixture.

c Based on ^1^H NMR determination of the purified copolymers.

d TON is the number of moles of CHO consumed per mole of the catalyst.

e TOF = TON per h.

f Determined by GPC in THF, calibrated using polystyrene standards.

Owing to the excellent copolymerization performances of nickel complexes **2** and **6** in the CO_2_/CHO system, their catalytic features were further investigated in detail. The catalytic results are summarized in Table 2. Under the copolymerization conditions of [CHO]_0_/[catalyst]_0_ = 5000/1 with an initial CO_2_ pressure of 20.7 bar for 2 h, the effect of reaction temperature on the copolymerization catalysis was first examined. Both complexes exhibited an enhancement in activity with increasing temperature. The bis­(acetate) complex **2** showed good activity and product selectivity within the range of 100–130 °C, reaching a TOF of 1125 h^–1^ at 130 °C (Figure 4a). The CHC formation (4%) was found at 140 °C compared to that (<1%) at 130 °C; this is likely due to backbiting reactions at elevated temperature, which is also consistent with the lower molar mass of the obtained PCHC. Notably, the dichloride complex **6** displayed the superior thermal tolerance: it reached a TOF of 1150 h^–1^ at 140 °C while maintaining high product selectivity and no detectable CHC (Figure 4b). Upon further increasing the reaction temperature to 150 °C, the CHC production (5%) and the lower-molecular-weight PCHC were observed, a behavior similar to that of complex **2**. Although this type of nickel complex exhibited relatively good thermal stability, backbiting side reactions and an overall decrease in catalytic activity were observed under the excessively high temperature. This behavior may be attributed to the partial thermal decomposition of the active nickel species or catalyst deactivation. The controllability of the CO_2_/CHO copolymerization catalyzed by binickel complexes **2** and **6** was further examined within the reaction time ranges of 0.5–2 h. Both complexes **2** (at 130 °C) and **6** (at 140 °C) exhibited excellent control over the molecular weights of the resulting PCHCs. As shown in Figure 5, a linear correlation between CHO conversion (approximately 20–45%) and the molecular weight of PCHC (10–20 kg·mol^–1^) could be observed, while the dispersity (*D̵*) values remained narrow (<1.3) throughout the reaction. Remarkably, at 0.5 h, nickel catalysts **2** and **6** displayed the highest catalytic activities in this study, with excellent TOF values of 2100 and 2200 h^–1^, respectively (Table 2, entries 10 and 13). Under the condition of the lower catalyst loadings having an increased feed ratio of [CHO]_0_/[catalyst]_0_ = 15,000/1 (Table 2, entries 16 and 17), the performances of dinickel catalysts **2** and **6** were evaluated. Both systems achieved CHO conversions above 60% at 120 °C for 24 h, with turnover numbers (TONs) of 9150 and 9,300, respectively. The resulting PCHCs reached molecular weights of up to ∼36 kg·mol^–1^, representing the highest TONs and molecular weights obtained in this study. These results highlight the excellent catalytic productivity of the dinickel catalyst systems developed in this study for CO_2_/CHO copolymerization. The microstructure of PCHC produced by Ni catalyst **6** was further analyzed by MALDI-TOF mass spectrometry. As shown in Figure S5, the MALDI-TOF mass spectrum displays two series of peaks with an *m*/*z* interval of 142, confirming the alternating enchainment of CO_2_ and CHO and supporting the speculation drawn from the ^1^H NMR analysis. Further analysis of these peak series reveals that one set can be assigned to PCHC chains initiated by chloride ion species, with the cyclohexene end groups that are likely derived from the originally produced cyclohexanol chain ends through dehydration reactions during the sample preparation or MALDI-TOF measurement, while the lower-intensity series can be reasonably attributed to PCHC diols formed through chain-transfer reactions involving trace proton sources, such as water or cyclohexane diol, during copolymerization.^73^ This interpretation is also consistent with the bimodal molecular weight distributions observed in the GPC traces (Figure S6).

**Table 2:**
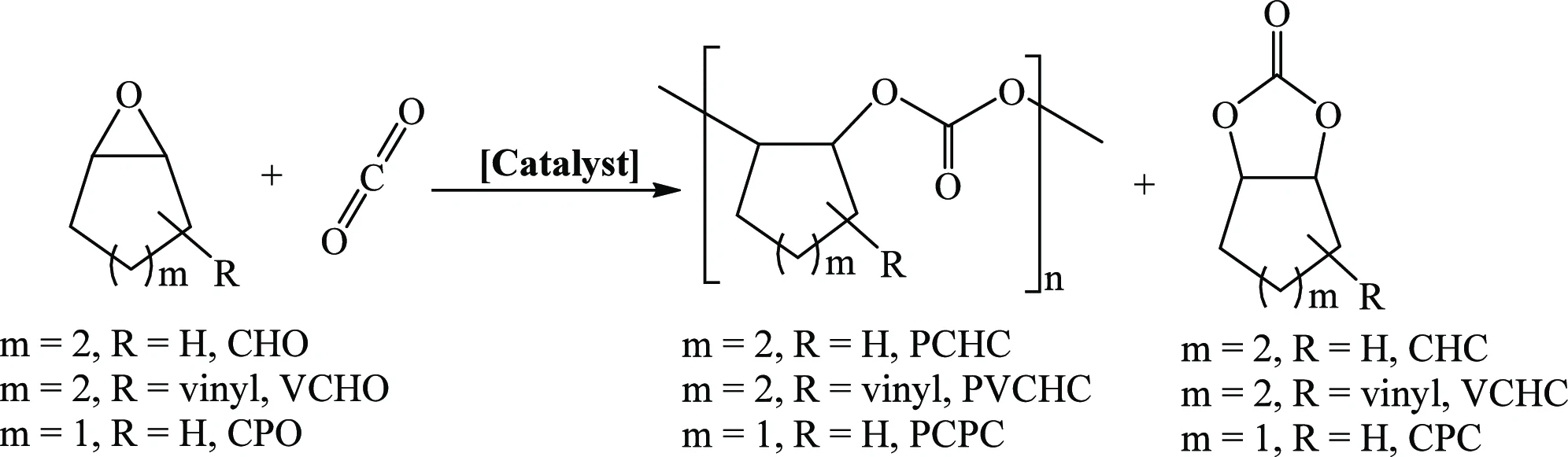
Copolymerization of CO_2_ and CHO Mediated by Dinuclear Nickel Complexes **2** and **6** ^a^

entry	catalyst	temp (°C)	time (h)	% CHO conv^b^	% copolymer^b^ (% CO_3_ linkages)^c^	TON^d^	TOF/h^–1^ ^e^	*M*_n_ [D̵]^f^ (g mol^–1^)
1	**2**	100	2	15	>99 (>99)	750	375	6100 [1.26]
2	**2**	120	2	33	>99 (>99)	1650	825	14,000 [1.21]
3	**2**	130	2	45	>99 (>99)	2250	1125	20,000 [1.23]
4	**2**	140	2	42	96 (>99)	2100	1050	17,000 [1.24]
5	**6**	100	2	12	>99 (>99)	600	300	6000 [1.23]
6	**6**	120	2	31	>99 (>99)	1550	775	14,000 [1.21]
7	**6**	130	2	41	>99 (>99)	2050	1025	18,000 [1.21]
8	**6**	140	2	46	>99 (>99)	2300	1150	20,900 [1.25]
9	**6**	150	2	41	95 (>99)	2050	1025	16,000 [1.27]
10	**2**	130	0.5	21	>99 (>99)	1050	2100	11,600 [1.24]
11	**2**	130	1	30	>99 (>99)	1500	1500	15,100 [1.23]
12	**2**	130	1.5	37	>99 (>99)	1850	1233	17,100 [1.23]
13	**6**	140	0.5	22	>99 (>99)	1100	2200	10,700 [1.23]
14	**6**	140	1	31	>99 (>99)	1550	1550	14,100 [1.23]
15	**6**	140	1.5	38	>99 (>99)	1900	1267	17,100 [1.23]
16^g^	**2**	120	24	61	>99 (>99)	9150	381	35,800 [1.17]
17^g^	**6**	120	24	62	99 (>99)	9300	388	35,700 [1.17]
18^h^	**6**	120	2	57	97 (>99)	912	456	48,800 [1.44]
19^i^	**6**	120	2	43	97 (>99)	1376	688	41,600 [1.23]
20^j^	**6**	120	2	56	<1	896	448	NR
21^j^	**6**	100	2	36	<1	576	288	NR

a Copolymerization conditions: 0.03125 mmol of catalyst, 156.25 mmol of CHO, [CHO]_0_/[catalyst]_0_ = 5000/1, *p*CO_2_^0^ = 20.7 bar.

b Based on ^1^H NMR analysis of the reaction mixture.

c Based on ^1^H NMR determination of the purified copolymers.

d TON = number of moles of CHO consumed per mole of the catalyst.

e TOF = TON per hour.

f Determined by GPC in THF and calibrated using polystyrene standards.

g 0.010417 mmol catalyst, [CHO]_0_/[catalyst]_0_ = 15,000/1.

h 50 mmol of 4-vinyl-1,2-cyclohexene oxide (VCHO) was used as the monomer.

i 100 mmol of VCHO was used as the monomer.

j 50 mmol of cyclopentene oxide (CPO) as monomer, NR = not reported.

**Figure 4:**
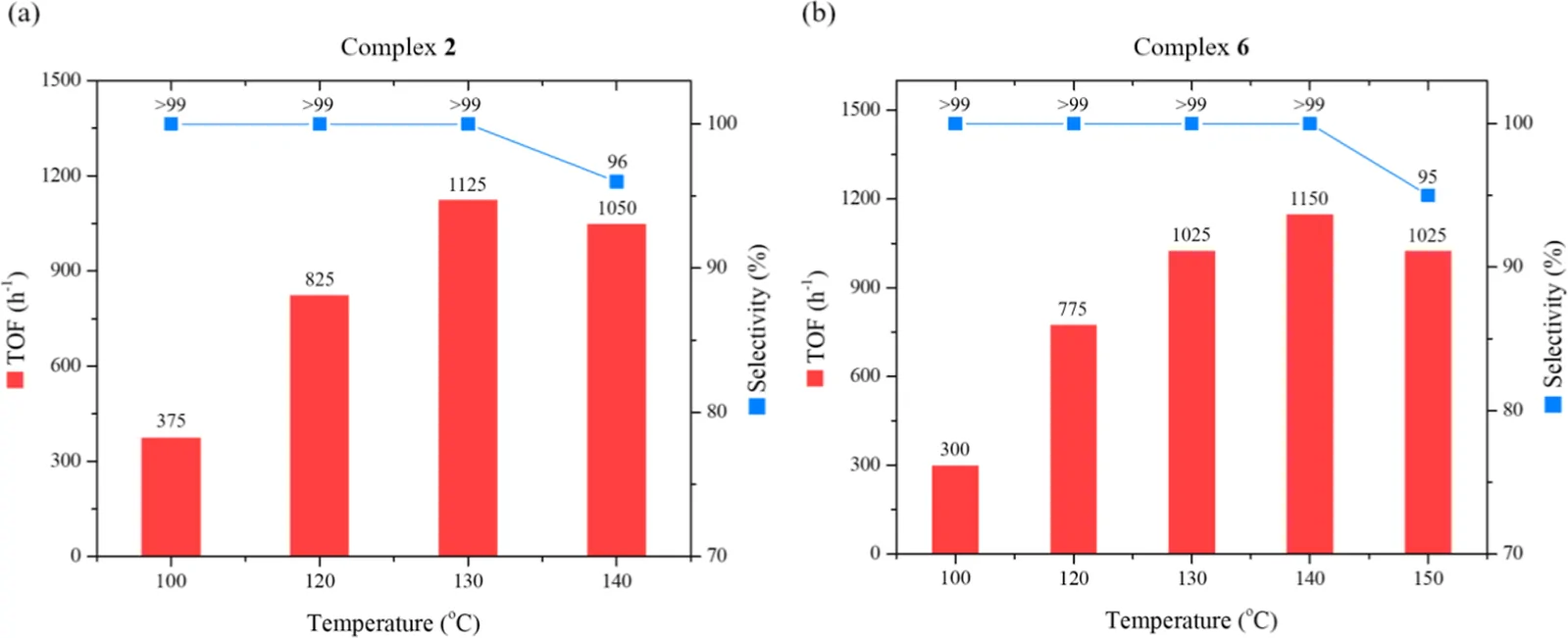
Effect of reaction temperature on the catalytic performance of (a) complex **2** and (b) complex **6**.

**Figure 5:**
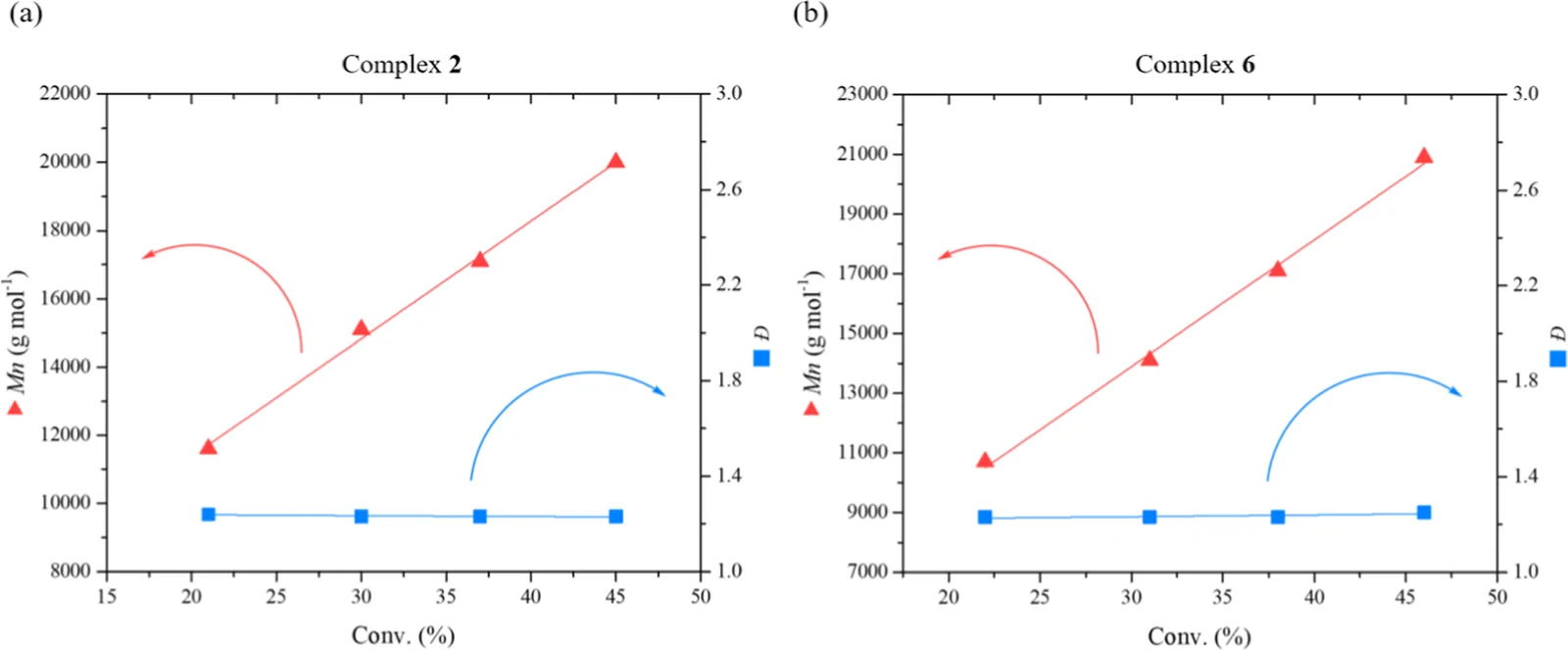
Plot of *M*_n_ and *D̵* versus CHO conversion for the copolymerization of CO_2_ and CHO mediated by (a) complex **2** and (b) complex **6**.

Motivated by the satisfactory catalytic efficiency of binuclear Ni complex **6** in the CO_2_/CHO copolymerization, we further explored the monomer scope of other alicyclic epoxides toward CO_2_ copolymerization, as summarized in entries 18–21 of Table 2. Complex **6** efficiently catalyzed the copolymerization of CO_2_ with 1600 equiv 4-vinyl-1,2-cyclohexene oxide (VCHO), affording an alternating poly­(vinylcyclohexene carbonate) (PVCHC) with high product selectivity (97%) and a large molecular weight (*M*_n_ = 48.8 kg·mol^–1^). Raising the monomer-to-catalyst ratio to [VCHO]_0_/[**6**]_0_ = 3200 maintained the high copolymerization efficiency, delivering a TON of 1376 and a TOF of 688 h^–1^ (Table 2, entry 19), which is comparable to the copolymerization activity observed with CHO as the epoxide monomer (Table 1, entry 6). However, only cyclopentene carbonate was obtained while cyclopentene oxide (CPO) was used as the monomer, and no corresponding polycarbonate formation was detected even when the reaction temperature was diminished to 100 °C. Based on a recent mechanistic study by Williams’ group, CPO exhibited the weakest binding affinity toward the catalytic metal center among common epoxides, resulting in inefficient epoxide activation and slow propagation. Such sluggish chain growth was expected to favor the competing backbiting pathway, thereby leading to the preferential formation of the cyclic carbonate rather than polycarbonate.^74^

We further investigated the terpolymerization of CO_2_, CHO, and CHO-related derivatives, and the corresponding polymerization data are summarized in Table 3. Using nickel complex **6** as the catalyst, the terpolymerization of CO_2_, CHO, and VCHO was carried out at [epoxides]_0_/[**6**]_0_ = 3,200, 120 °C, and 20.7 bar, with [CHO]_0_/[VCHO]_0_ feed ratios of 80/20 and 20/80, respectively. The results shown in entries 1 and 2 of Table 3 displayed that the poly­(cyclohexene carbonate)-*co*-poly­(vinylcyclohexene carbonate) (PCHC-*co*-PVCHC) could be successfully afforded, as determined by ^1^H NMR spectroscopy (Figure S7). Furthermore, complex **6** was capable of producing PCHC-*co*-PVCHC with a >99% carbonate linkage selectivity. Based on the previous studies on the synthesis of PCHC-*co*-PVCHC by the dinuclear nickel nitrophenolate-based complex, the microstructure of such terpolymer was suggested to be a random form.^62^ In addition, based on the integral ratios of the characteristic signals associated with the PCHC and PVCHC segments in the spectra, adjusting the [CHO]_0_/[VCHO]_0_ feed ratio allows for the facile preparation of (PCHC)_n_-*co*-(PVCHC)_m_ with different n/m ratios. The resulting copolymers exhibited repeat unit ratios (n/m) of 71/29 and 16/84, respectively. The slightly higher incorporation of PVCHC units relative to the feed ratio suggests that dinickel complex **6** performs a slightly higher copolymerization reactivity toward VCHO than CHO. Recently, Ema’s team reported the utilization of a bifunctional porphyrin-based aluminum complex for terpolymerization catalysis, enabling the synthesis of CO_2_-based polycarbonates bearing acrylate functionalities on the polymeric backbone.^75^ Inspired by this work, the acrylate-containing epoxide monomer of 4-(acryloxymethyl)-1,2-cyclohexene oxide, denoted as AMCHO, was selected for terpolymerization with CHO and CO_2_. The reaction was carried out at [epoxides]_0_/[**6**]_0_ = 3,200, 100 °C, and 20.7 bar, with a [CHO] _0_/[AMCHO]_0_ feed ratio of 90/10, in the presence of 4-methoxyphenol as an inhibitor for acrylate polymerization (Table 3, entries 3 and 4). The polymerization data showed that the molecular weight increased with reaction time, reaching 14.4 kg·mol^–1^ after 10 h. According to ^1^H NMR analysis depicted in Figure S8, the obtained poly­(cyclohexene carbonate)-*co*-poly­(acryloxymethylcyclohexene carbonate) (PCHC-*co*-PAMCHC) revealed no detectable ether linkages. The PAMCHC content was determined to be 16% (Table 3, entry 4), indicating a higher incorporation of the acrylate-functionalized carbonate units than that of the feed ratio, consistent with that observed for PCHC-*co*-PVCHC. However, insoluble solids were observed after 12 h. Attempts to optimize the reaction conditions were less than ideal. For instance, lowering the temperature to 80 °C resulted in very low catalytic activity. When the [CHO]_0_/[AMCHO]_0_ feeding ratio was adjusted to 80/20, insoluble solids formed after 6 h. Based on these preliminary observations, the acrylate group in PCHC-*co*-PAMCHC exhibits a more pronounced tendency toward cross-linking compared to the vinyl group in PCHC-*co*-PVCHC. This undesired cross-linking during copolymerization may potentially be suppressed by decreasing the catalyst loading, adjusting the initial [CHO]_0_/[AMCHO]_0_ feed ratio, or employing alternative radical inhibitors. Although the optimal conditions for such CO_2_ copolymerization still require further investigation, complex **6** represents, to the best of our knowledge, the first nickel catalyst capable of producing acrylate-functionalized polycarbonates via CO_2_ copolymerization.

**Table 3:**
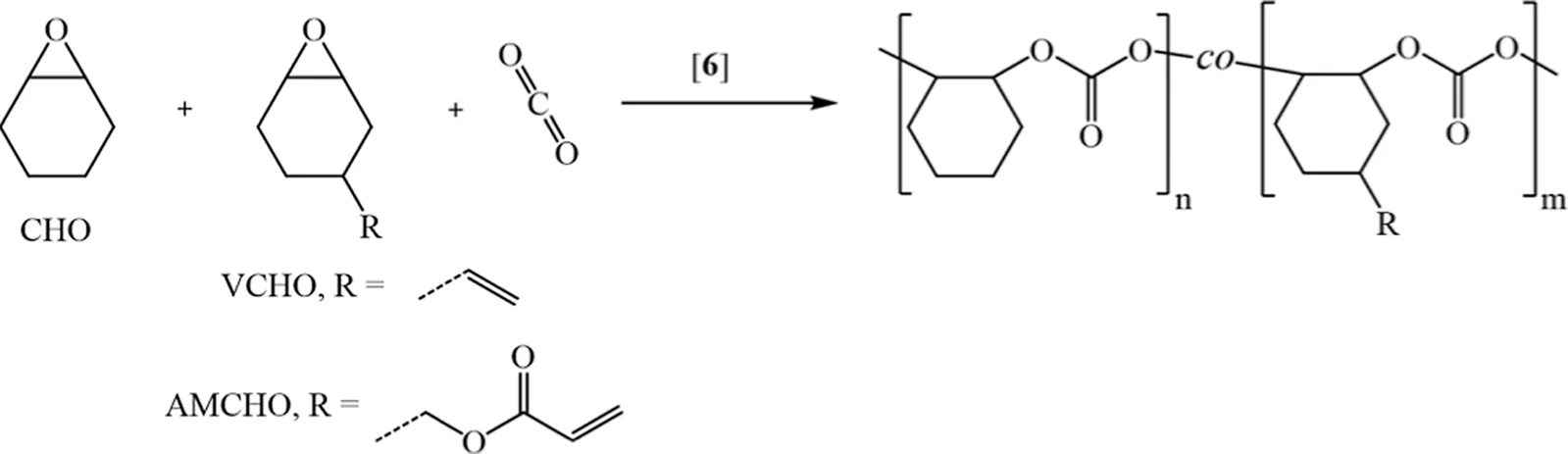
Terpolymerization of CO_2_ and Alicyclic Epoxides Mediated by Dinuclear Nickel Complex **6** ^a^

entry	RCHO	[CHO]_0_/[RCHO]_0_:[6]_0_	temp (°C)	time (h)	% yield^b^	%CO_3_ linkages^c^	%composition (n: m)^c^	*M*_n_ [*D̵*]^d^ (g mol^–1^)
1	VCHO	2560:640:1	120	2	40	>99	71:29	21,300 [1.17]
2	VCHO	640:2560:1	120	2	34	>99	16:84	37,900 [1.29]
3^e^	AMCHO	2880:320:1	100	8	43	>99	82:18	9700 [1.29]
4^e^	AMCHO	2880:320:1	100	10	58	>99	84:16	14,400 [1.33]

a Copolymerization conditions: 0.03125 mmol of catalyst, 100.0 mmol of total epoxides, and *p*CO_2_^0^ = 20.7 bar (300 psi).

b Isolated yields of the resultant copolymers.

c Based on ^1^H NMR determination of the purified copolymers (Figures S7 and S8).

d Determined by GPC in THF and calibrated using polystyrene standards.

e 4-Methoxyphenol (1 mol % AMCHO) was used as an acrylate group polymerization inhibitor.

A comparison with previously reported dinickel catalysts for CO_2_/CHO copolymerization from our group is summarized in Scheme 3. The bis­(acetate) complex **2** exhibits higher activity than complexes **A** and **B**, comparable performance to **D**, but remains inferior to benzotriazole-derived diamine-bisphenolate complex **C** (TOF = 7800 h^–1^).^57,61,68,69^ The dichloride complex **6** shows ROCOP activity comparable to dihalide complexes **E**–**G** under similar conditions.^61,65,70^ Notably, complexes **2** and **6** display the highest TOF values among our salen-based dinickel systems, and their maximum TON values (>9000) exceed those previously reported (TON = 6000 for **F**; 6200 for **G**). Furthermore, representative salen-derived mononuclear and binuclear complexes as well as multimetallic catalysts reported in the literature under similar copolymerization conditions are also included for comparisons (Scheme 4). Some catalysts have been evaluated for CO_2_/CHO copolymerization at temperatures above 120 °C, such as the Mg­(II)/Co­(II) catalyst **K** supported by the reduced Robson-type ligand, which exhibits notable activity at 140 °C (TOF = 12,460 h^–1^),^41^ and the macrocyclic-based Co_3_(II)/Nd­(III) catalyst **P**, which exhibits a high catalytic performance, affording a TOF of 1625 h^–1^ and a TON of 13,000 at 130 °C.^51^ Other catalysts also display excellent copolymerization activity at 120 °C, including bifunctional salen-type Co­(III) complex **H** (TOF = 6105 h^–1^),^76^ crown ether-modified salen-based Co­(III)/Na­(I) complex **L** (TOF = 4343 h^–1^),^43^ and Zn­(II)_2_/Na­(I) complex **O** (TOF = 2900 h^–1^).^48^ In addition, the anilido-aldimine dizinc complex **N** exhibits a TOF of 2880 h^–1^ and a TON of 9440 at 80 °C,^25^ while the Nd­(III)_2_/Zn­(II) complex **Q** coordinated on the diamine-tris­(phenolato) ligand gives a TOF of 5400 h^–1^ at 90 °C,^77^ indicating outstanding activity at the lower temperature. In comparison, the dinickel catalysts **2** and **6** display somewhat lower activity (TOF = 896 h^–1^ for **2**; 768 h^–1^ for **6**) at 120 °C. Nevertheless, at elevated temperatures (≥130 °C), both **2** and **6** exhibit excellent polymerization selectivity, affording alternating CO_2_-based polycarbonates. Moreover, the triphenylimidazole-derived salen-type ligands and their related metal complexes are relatively straightforward to synthesize as well as such nickel catalysts are stable in air. These features highlight the potential of this ligand platform for further development in the catalytic epoxide copolymerization.

**Scheme 3:**
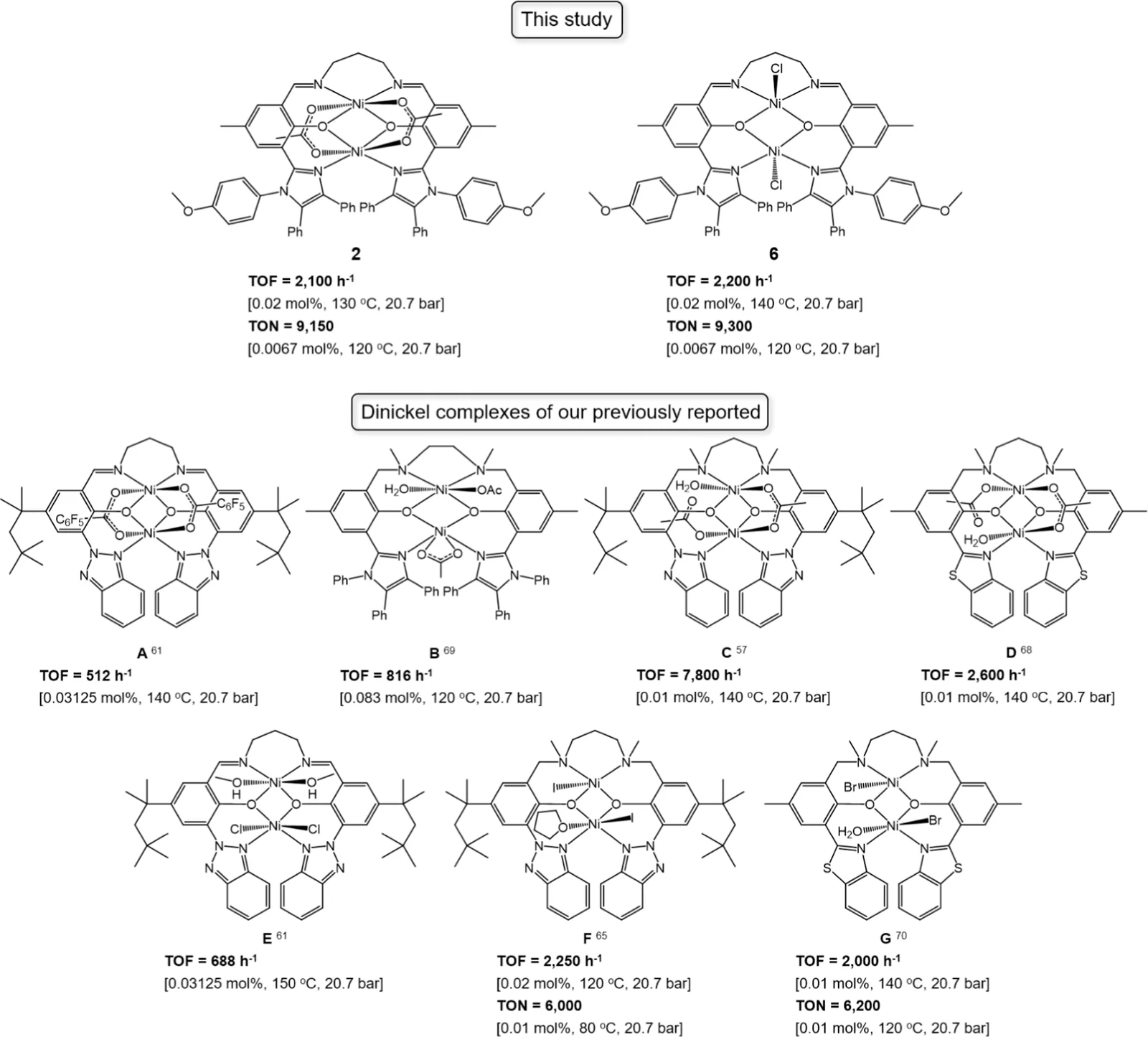
Selected Dinickel Complexes and Their Catalytic Performances for Copolymerization of CO_2_ and CHO

**Scheme 4:**
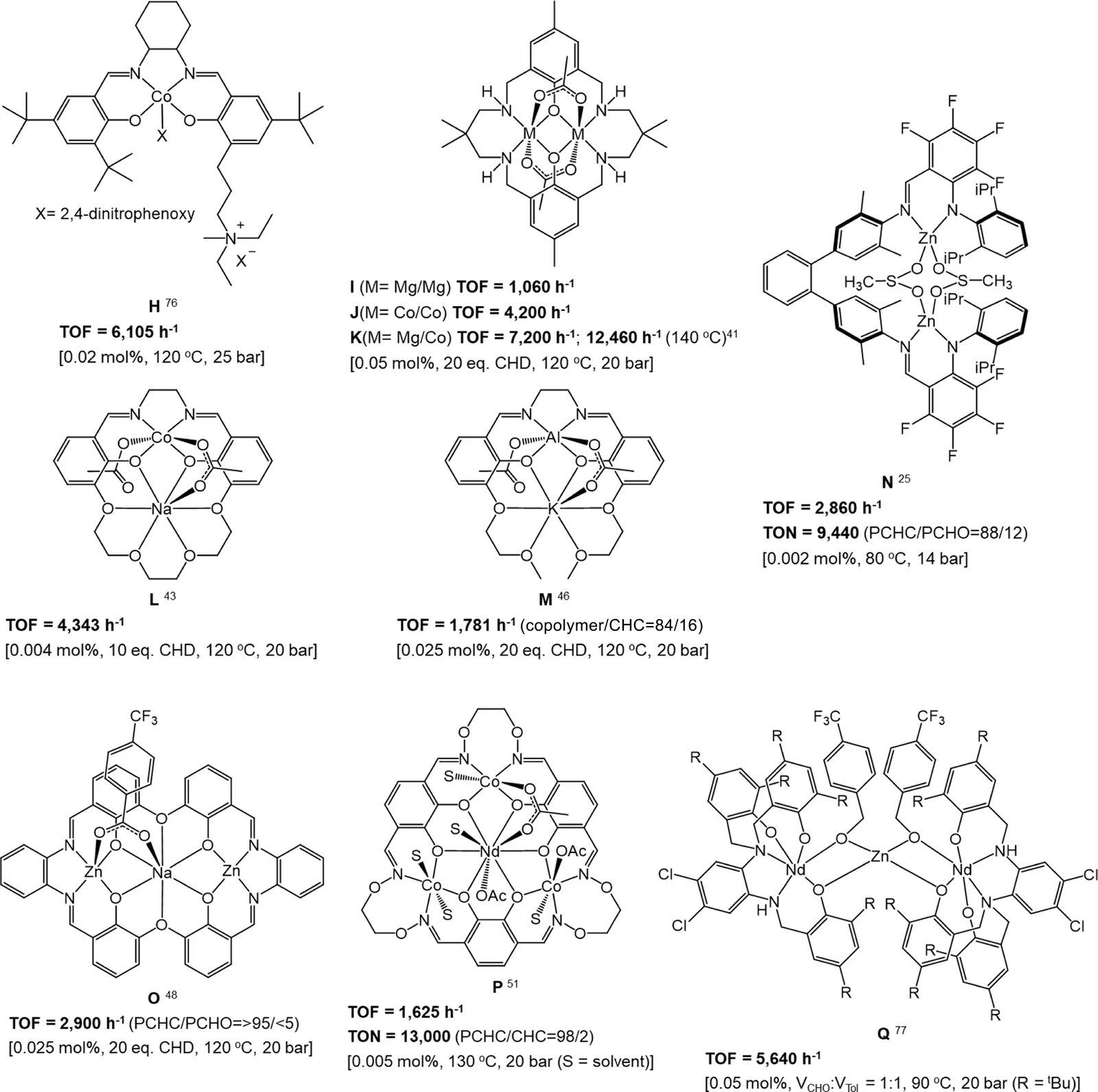
Selected Metal Complexes in the Literature and Their Catalytic Performances for Copolymerization of CO_2_ and CHO

### Kinetic Studies of CO_2_/CHO ROCOP Catalyzed by Dinickel Complex **6**

To understand the catalytic behavior of imidazole-based salen-like dinuclear nickel complexes toward the CO_2_/CHO ring-opening copolymerization (ROCOP), the polymerization kinetics of CO_2_ and CHO was thoroughly investigated. In our previous reports, the kinetic study on the dichloro-coordinated salen-type dinuclear nickel complexes had not yet been conducted, and the methoxy-substituted complex of this kind was shown to be the most effective. Therefore, complex **6** was selected as the representative catalyst for the further kinetic discussion. First, the effect of CO_2_ pressure on the copolymerization activity was examined under the conditions of [CHO]_0_/[**6**]_0_ = 3200/1 at 120 °C for 1 h. The results, as shown in Figure 6a and Table S3, indicate that within the initial CO_2_ pressure range of 13.8–34.5 bar (200–500 psi), comparable catalytic activities were observed, suggesting a zero-order dependence on CO_2_ concentration. Under the conditions of initial CO_2_ pressure 20.7 bar and 120 °C, the relationship between CHO conversion and copolymerization time at different catalyst concentrations was examined. As shown in Figure 6b and Tables S4 and S5, a linear relationship between ln­([CHO]_0_/[CHO]_t_) and time was observed, indicating a first-order dependence on the CHO concentration of such copolymerization. Moreover, identical linear dependencies were obtained at various catalyst concentrations. Accordingly, the rate equation for the ROCOP can be expressed as – *d*[CHO]/*d*t = *k*_obs_[CO_2_]^0^[CHO]^1^, where *k*_obs_ is the observed rate constant. A linear plot of ln­(*k*_obs_) versus ln­[**6**]_0_ afforded an apparent catalyst order of 1.24, indicating a slightly greater than first-order dependence on catalyst concentration (Figure 6c). To further examine this deviation, *k*_obs_ was plotted as a function of catalyst concentration. The resulting plot exhibited a nonzero *x*-axis intercept, corresponding to a limiting catalyst concentration of 0.508 mM. Such behavior has previously been reported for ROCOP systems and is generally attributed to partial catalyst deactivation by trace impurities (e.g., residual protic species in the monomer), leading to an inactive catalyst fraction at low catalyst concentrations.^39^ Consequently, the apparent catalyst order slightly greater than unity is likely an apparent effect arising from the limiting catalyst concentration. Based on the above kinetic analysis, the overall rate law follows the zero-order kinetics with respect to CO_2_ pressure as well as first-order kinetics with respect to both CHO and complex **6** concentrations. As a result, the overall rate equation can be presented as −*d*[CHO]/*d*t = *k*_p_[**6**]^1^[CO_2_]^0^[CHO]^1^, where *k*_p_ is the propagation rate constant. These findings are consistent with previous kinetic studies on dinuclear nickel­(II) complexes by our group.^57–59,68–70^

**Figure 6:**
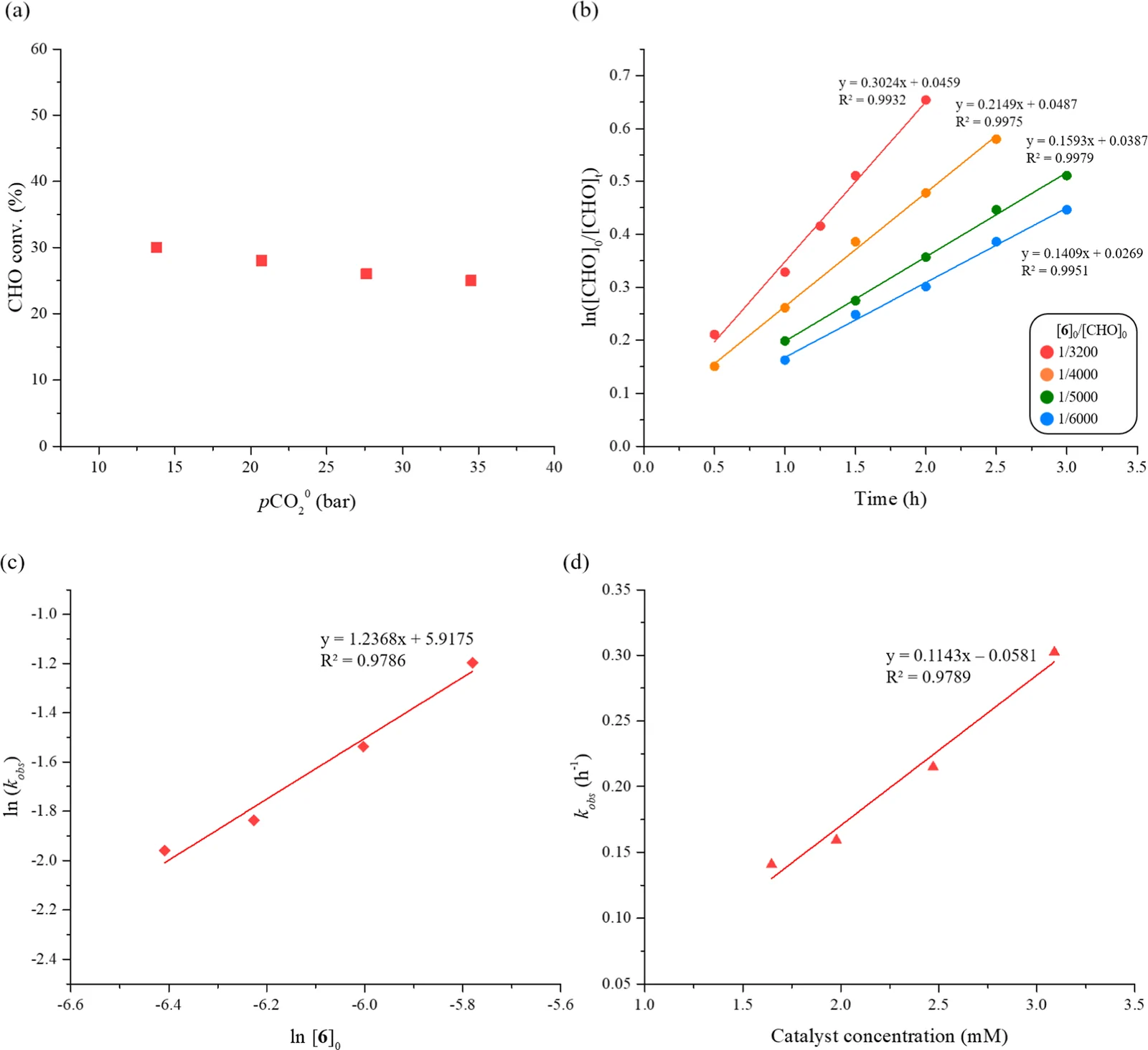
Kinetics for CO_2_/CHO ROCOP catalyzed by complex **6**. (a) Reaction order in CO_2_ concentration was determined by the plot of CHO conversion with different initial CO_2_ pressures. (b) Kinetic plots of ln­([CHO]_0_/[CHO]_t_) vs time under various monomer-to-catalyst ratios. (c) A linear plot of ln­(*k*_obs_) vs ln­[**6**]_0_ displaying the first-order dependence on dinickel catalyst concentration. (d) A linear plot of the observed rate constant (*k*_obs_) vs the concentration of **6** for CO_2_/CHO ROCOP.

## Conclusions

In this study, a series of dinuclear nickel complexes based on triphenylimidazole-containing salen-type ligands were developed, and their efficient catalytic performance toward the ROCOP of CO_2_ with alicyclic epoxides was demonstrated. Catalysis results indicate that ligand modifications on the phenyl rings possess a significant impact on catalytic performance, with the methoxy-substituted complexes **2** and **6** showing notably enhanced activity. This enhancement is likely associated with electronic modulation of the ligand framework, which facilitates substrate activation and improves catalytic efficiency. In particular, the dichloride complex **6** not only exhibits excellent activity and selectivity in CO_2_/CHO copolymerization, with a TOF of 2200 h^–1^ and a TON of 9300 under the optimized conditions, but also reveals unique capability in terpolymerization, enabling the synthesis of functionalized CO_2_-based polycarbonates containing vinyl or acrylate moieties. Kinetic studies of CO_2_/CHO ROCOP catalyzed by **6** revealed a rate law of −*d*[CHO]/*d*t = *k*_p_[**6**]^1^[CHO]^1^, indicating first-order dependence on both the dinickel catalyst and CHO concentrations. These findings provide insights into the structure–activity relationship of dinuclear nickel complex-mediated CO_2_/epoxide ROCOP. Furthermore, the modular preparation of imidazole-containing phenolate ligands, together with the fine-tuning of auxiliary coligands, offer a promising strategy for the further development of high-efficiency dinuclear/multinuclear metal catalysts.

## Experimental Section

The details of chemicals, measurements, and general procedure for the X-ray crystallography and catalytic studies of CO_2_/epoxides ROCOP are depicted in the Supporting Information. No uncommon hazards were noticed to be derived from the experimental work carried out.

### Synthesis of ^*OMe*^TPImP-H

Based on our previous works,^69^ 2-hydroxy-5-methylbenzaldehyde (2.04 g, 15.0 mmol) and benzil (3.15 g, 15.0 mmol) were dissolved in 120 mL of glacial acetic acid at room temperature. *p*-Anisidine (2.77 g, 22.5 mmol) was added dropwise to this solution, and ammonium acetate (5.78 g, 75.0 mmol) was added subsequently. The mixture was heated at 110 °C for 24 h and then cooled to room temperature. After the termination of the reaction, the resultant solution was poured into a large amount of water, and the formation of a white precipitate was observed. Recrystallization from an ethyl acetate solution produced 3.14 g of gray powder with an overall yield of 48%. ^1^H NMR (600 MHz, CDCl_3_, ppm): δ 7.59–7.54 (m, 2H, Ar–*H*), 7.31–7.23 (m, 5H, Ar–*H*), 7.21 (t, *J* = 7.32 Hz, 1H, Ar–*H*), 7.16 (dd, *J* = 7.97, 1.56 Hz, 2H, Ar–*H*), 7.12–7.06 (m, 2H, Ar–*H*), 7.02–6.94 (m, 2H, Ar–*H*), 6.87 (d, *J* = 8.88 Hz, 2H, Ar–*H*), 6.38 (s, 1H, Ar–*H*), 3.80 (s, 3H, OC*H*_3_), 1.94 (s, 3H, Ar–C*H*_3_). ^13^C NMR (151 MHz, CDCl_3_, ppm): δ 159.74, 156.14, 145.14, 134.87, 133.17, 131.27, 130.52, 130.46, 129.91, 129.76, 129.63, 128.39, 128.29, 128.17, 126.82, 126.78, 126.52, 126.37, 117.20, 114.49, 112.59 (Ar–*C*), 55.43 (O*C*H_3_), 20.59 (Ar-*C*H_3_).

### Synthesis of ^*Cl*^TPImP-H

This compound was synthesized as mentioned above for ^OMe^TPImP-H, except for 4-chloroaniline (2.87 g, 22.5 mmol) as the reactants. Recrystallization from an ethyl acetate solution produced 2.99 g of light pink powder with an overall yield of 46%. ^1^H NMR (600 MHz, CDCl_3_, ppm): δ 13.02 (s, 1H, O*H*), 7.57–7.52 (m, 2H, Ar–*H*), 7.37–7.19 (m, 8H, Ar–*H*), 7.16–7.09 (m, 4H, Ar–*H*), 6.99 (s, 2H, Ar–*H*), 6.31 (s, 1H, Ar–*H*), 1.95 (s, 3H, Ar–C*H*_3_). ^13^C NMR (151 MHz, CDCl_3_, ppm): δ 156.07, 144.97, 135.77, 135.46, 135.00, 132.91, 131.27, 130.78, 130.13, 129.94, 129.64, 129.57, 128.61, 128.24, 127.04, 126.84, 126.77, 126.39, 126.35, 117.41, 112.25 (Ar–*C*), 20.56 (Ar-*C*H_3_).

### Synthesis of ^OMeAld^TPImP-H

As per our previous works,^71^ a 250 mL round-bottom flask was placed with ^*OMe*^TPImP-H (3.03 g, 7.0 mmol) and hexamethylenetetramine (9.81 g, 70.0 mmol). To this was added trifluoroacetic acid (50 mL), and the resulting mixture was heated to 140 °C under reflux for 20 h. The resultant solution was then poured into 4 N HCl_(aq)_ (90 mL) and stirred for another 3 h. The mixture was then extracted with dichloromethane, and the organic layer was extracted to neutrality. The volatile components were removed in vacuum, and the final residue was washed with hexane, and the resultant solids were collected by filtration and dried under vacuum to give yellow solids. Yield: 2.56 g (79%). ^1^H NMR (600 MHz, CDCl_3_, ppm): δ 10.56 (s, 1H, Ald-*H*), 7.53 (d, *J* = 7.63 Hz, 2H, Ar–*H*), 7.49 (s, 1H, Ar–*H*), 7.31–7.26 (m, 4H, Ar–*H*), 7.25–7.20 (m, 2H, Ar–*H*), 7.16 (d, *J* = 6.75 Hz, 2H, Ar–*H*), 7.08 (d, *J* = 8.95 Hz, 2H, Ar–*H*), 6.87 (d, *J* = 8.95 Hz, 2H, Ar–*H*), 6.67 (s, 1H, Ar–*H*), 3.81 (s, 3H, OC*H*_3_), 1.97 (s, 3H, Ar–C*H*_3_). ^13^C NMR (151 MHz, CDCl_3_, ppm): δ 190.80 (Ald-*C*), 159.91, 159.86, 144.02, 135.06, 133.18, 132.73, 131.19, 130.92, 129.49, 129.43, 129.24, 128.84, 128.49, 128.26, 127.09, 126.83, 126.70, 123.64, 114.65 (Ar–*C*), 55.50 (O*C*H_3_), 55.45 (O*C*H_3_), 20.37 (Ar-*C*H_3_).

### Synthesis of ^ClAld^TPImP-H

This compound was synthesized as mentioned above for ^*OMeAld*^TPImP-H, except for ^*Cl*^TPImP-H (3.06 g, 7.0 mmol) as the reactants. Recrystallization from an ethyl acetate solution produced 2.38 g of light pink powder with an overall yield of 73%. ^1^H NMR (600 MHz, CDCl_3_, ppm): δ 13.56 (s, 1H, O*H*), 10.44 (s, 1H, Ald-*H*), 7.56–7.51 (m, 2H, Ar–*H*), 7.50 (s, 1H, Ar–*H*), 7.35–7.30 (m, 3H, Ar–*H*), 7.30–7.27 (m, 2H, Ar–*H*), 7.27–7.20 (m, 3H, Ar–*H*), 7.13 (d, *J* = 1.76 Hz, 2H, Ar–*H*), 7.09 (d, *J* = 8.51 Hz, 2H, Ar–*H*), 6.76 (s, 1H, Ar–*H*), 2.04 (s, 3H, Ar–C*H*_3_). ^13^C NMR (151 MHz, CDCl_3_, ppm): δ 191.42 (Ald-*C*), 159.36, 143.76, 136.00, 135.28, 135.12, 134.02, 132.64, 131.14, 130.49, 129.98, 129.68, 129.58, 129.25, 128.75, 128.70, 128.31, 127.24, 126.95, 123.35, 115.05­(Ar–*C*), 20.35 (Ar-*C*H_3_).

### Synthesis of Ligand Precursor ^*H3C*^L-H_2_

A mixture of ^*HAld*^TPImP-H (1.72 g, 4.0 mmol) and 1,3-diaminopropane (0.16 g, 2.2 mmol) in diethyl ether (150 mL) was stirred for 24 h, and the formation of a yellow precipitate was observed during the process. The resulting precipitate was collected by filtration and dried under vacuum to give a yellow solid. Yield: 1.66 g (93%). ^1^H NMR (600 MHz, CDCl_3_, ppm): δ 13.61 (s, 1H, O*H*), 8.25 (s, 1H, N = C*H*-Ar), 7.63 (d, *J* = 7.11 Hz, 2H, Ar–*H*), 7.26 (t, *J* = 7.49 Hz, 6H, Ar–*H*), 7.22–7.12 (m, 6H, Ar–*H*), 7.11–7.05 (m, 3H, Ar–*H*), 3.55 (t, *J* = 6.58 Hz, 2H, N–C*H*_2_), 2.23 (s, 3H, Ar–C*H*_3_), 1.95 (p, *J* = 6.66 Hz, 1H, N–CH_2_–C*H*_2_). ^13^C NMR (151 MHz, CDCl_3_, ppm): δ 164.28 (N = *C*H–Ar), 157.41, 144.74, 137.80, 136.98, 134.78, 134.42, 132.05, 130.97, 130.72, 129.78, 128.26, 127.93, 127.81, 127.74, 127.56, 127.48, 126.99, 126.35, 118.79 (Ar–*C*), 56.43 (N–*C*H_2_), 31.21 (N–CH_2_–*C*H_2_), 20.15 (Ar-*C*H_3_).

### Synthesis of Ligand Precursor ^*OMe3C*^L-H_2_

This compound was synthesized as mentioned above for ^*H3C*^L-H_2_, except for ^*OMeAld*^TPImP-H (1.84 g, 4.0 mmol) as the reactants. Yield: 1.76 g (92%). ^1^H NMR (600 MHz, CDCl_3_, ppm): δ 13.66 (s, 1H, O*H*), 8.28 (s, 1H, N = C*H*-Ar), 7.66–7.60 (m, 2H, Ar–*H*), 7.32–7.25 (m, 6H, Ar–*H*), 7.20 (dd, *J* = 7.80, 1.89 Hz, 3H, Ar–*H*), 7.10 (s, 1H, Ar–*H*), 7.03 (d, *J* = 8.93 Hz, 2H, Ar–*H*), 6.68 (d, *J* = 9.08 Hz, 2H, Ar–*H*), 3.69 (s, 3H, OC*H*_3_), 3.59 (t, *J* = 6.51 Hz, 2H, N–C*H*_2_), 2.24 (s, 3H, Ar–C*H*_3_), 2.00 (t, *J* = 6.58 Hz, 1H, N–CH_2_–C*H*_2_). ^13^C NMR (151 MHz, CDCl_3_, ppm): δ 164.31 (N = *C*H–Ar), 158.63, 157.44, 144.94, 137.58, 134.71, 134.50, 131.97, 130.99, 130.83, 130.02, 129.80, 128.94, 128.27, 127.91, 127.69, 127.43, 126.96, 126.29, 118.87, 113.44 (Ar–*C*), 56.45 (N–*C*H_2_), 55.17 (O*C*H_3_), 31.24 (N–CH_2_–*C*H_2_), 20.20 (Ar-*C*H_3_).

### Synthesis of Ligand Precursor ^*Cl3C*^L-H_2_

This compound was synthesized as mentioned above for ^*H3C*^L-H_2_, except for ^*ClAld*^TPImP-H (1.86 g, 4.0 mmol) as the reactants. Yield: 1.80 g (93%). ^1^H NMR (600 MHz, CDCl_3_, ppm): δ 13.55 (s, 1H, O*H*), 8.18 (s, 1H, N = C*H*-Ar), 7.58 (d, *J* = 8.33 Hz, 2H, Ar–*H*), 7.34 (s, 1H, Ar–*H*), 7.24 (dt, *J* = 21.50, 7.42 Hz, 5H, Ar–*H*), 7.16 (dd, *J* = 18.09, 7.34 Hz, 3H, Ar–*H*), 7.08 (d, *J* = 8.63 Hz, 2H, Ar–*H*), 7.05 (s, 1H, Ar–*H*), 6.96 (d, *J* = 8.63 Hz, 2H, Ar–*H*), 3.52 (t, *J* = 6.51 Hz, 2H, N–C*H*_2_), 2.25 (s, 3H, Ar–C*H*_3_), 1.92 (t, *J* = 6.58 Hz, 1H, N–CH_2_–C*H*_2_). ^13^C NMR (151 MHz, CDCl_3_, ppm): δ 164.76 (N = *C*H–Ar), 157.35, 144.83, 138.24, 135.70, 135.15, 134.31, 133.23, 132.61, 130.98, 130.51, 129.61, 128.96, 128.49, 128.42, 128.00, 127.50, 127.34, 126.48, 118.92, 118.53 (Ar–*C*), 56.11 (N–*C*H_2_), 31.17 (N–CH_2_–*C*H_2_), 20.20 (Ar-*C*H_3_).

### Synthesis of Ligand Precursor ^*OMe4C*^L-H_2_

This compound was synthesized as mentioned above for ^*OMe3C*^L-H_2_, except for 1,4-diaminobutane (0.22 g, 2.2 mmol) as the reactants. Yield: 1.81 g (93%). ^1^H NMR (600 MHz, CDCl_3_, ppm): δ 13.71 (s, 1H, O*H*), 8.19 (s, 1H, N = C*H*-Ar), 7.58–7.52 (m, 2H, Ar–*H*), 7.19 (t, *J* = 7.56 Hz, 6H, Ar–*H*), 7.16–7.09 (m, 3H, Ar–*H*), 7.00 (s, 1H, Ar–*H*), 6.95 (d, *J* = 7.73 Hz, 2H, Ar–*H*), 6.60 (d, *J* = 8.88 Hz, 2H, Ar–*H*), 3.67–3.55 (m, 3H, OC*H*_3_), 3.49 (s, 2H, N–C*H*_2_), 2.17 (s, 3H, Ar–C*H*_3_), 1.62 (s, 2H, N–CH_2_–C*H*_2_). ^13^C NMR (151 MHz, CDCl_3_, ppm): δ 163.88 (N = *C*H–Ar), 158.53, 157.53, 144.95, 137.57, 134.69, 134.50, 132.02, 130.95, 130.80, 129.96, 129.74, 128.91, 128.25, 127.88, 127.65, 127.44, 126.83, 126.24, 118.78, 113.40 (Ar–*C*), 59.03 (N–*C*H_2_), 55.12 (O*C*H_3_), 28.20 (N–CH_2_–*C*H_2_), 20.20 (Ar-*C*H_3_).

### Synthesis of [^*H3C*^LNi_2_(CH_3_COO)_2_] (**1**)

A solution of nickel­(II) acetate tetrahydrate (0.5 g, 2.0 mmol) in methanol (10 mL) was added to a solution of ligand precursor ^*H3C*^ *L*-H_2_ (0.90 g, 1.0 mmol) and triethylamine (0.70 mL, 5.0 mmol) in methanol (50 mL). The resulting mixture was heated under reflux for 24 h and then cooled to an ambient temperature. The volatile components were removed in vacuo. The residue was diluted with dichloromethane and extracted with deionized water. The organic layers were concentrated by a vacuum evaporation. The final residue was recrystallized with dichloromethane and *n*-hexane, and the resulting precipitate was collected by filtration and dried under a vacuum to give green solids. Yield: 0.79 g (70%). Single crystals suitable for X-ray structural determination were grown from a mixed solution of dichloromethane and diethyl ether. Anal. calcd for C_65_H_54_N_6_Ni_2_O_6_: C, 68.93; H, 4.81; N, 7.42%. Found: C, 68.73; H, 4.70; N, 7.26%. ESI-MS (*m*/*z*): 1071.3 {100% [^*H3C*^LNi_2_(CH_3_COO)]^+^}.

### Synthesis of [^*OMe3C*^LNi_2_(CH_3_COO)_2_] (**2**)

This complex was synthesized as mentioned above for ^*H3C*^LNi_2_(CH_3_COO)_2_, except for ^*OMe3C*^L-H_2_ (0.96 g, 1.0 mmol) as the reactants. Yield: 1.03 g (86%). Anal. calc. for C_67_H_58_N_6_Ni_2_O_8_·0.5H_2_O: C, 66.97; H, 4.95; N, 6.99%. Found: C, 66.55; H, 5.13; N, 7.04%. ESI-MS (*m*/*z*): 1131.3 {100%, [^*OMe3C*^LNi_2_(CH_3_COO)]^+^}.

### Synthesis of [^*Cl3C*^LNi_2_(CH_3_COO)_2_] (**3**)

This complex was synthesized as mentioned above for ^*H3C*^LNi_2_(CH_3_COO)_2_, except for ^*Cl3C*^L-H_2_(0.97 g, 1.0 mmol) as the reactants. Yield: 0.92 g (77%). Single crystals suitable for X-ray structural determinations were grown from a mixed solution of acetone and *n*-hexane. Anal. calc. for C_65_H_52_Cl_2_N_6_Ni_2_O_6_·0.5CH_2_Cl_2_: C, 63.27; H, 4.26; N, 6.76%. Found: C, 62.91; H, 4.43; N, 6.77%. ESI-MS (*m*/*z*): 1141.3 {100%, [^*Cl3C*^LNi_2_(CH_3_COO)]^+^}.

### Synthesis of [^*OMe4C*^LNi_2_(CH_3_COO)_2_] (**4**)

This complex was synthesized as mentioned above for ^*H3C*^LNi_2_(CH_3_COO)_2_, except for ^*OMe4C*^L-H_2_ (0.97 g, 1.0 mmol) as the reactants. Yield: 0.90 g (75%). Single crystals suitable for X-ray structural determination were grown from a mixed solution of dichloromethane and *n*-hexane. Anal. calcd for C_68_H_60_N_6_Ni_2_O_8_: C, 67.69; H, 5.01; N, 6.96%. Found: C, 67.72; H, 5.18; N, 6.92%. ESI-MS (*m*/*z*): 1145.3 {100% [^*OMe4C*^LNi_2_(CH_3_COO)]^+^}.

### Synthesis of [^*H3C*^LNi_2_Cl_2_] (**5**)

This complex was synthesized as mentioned above for ^*H3C*^LNi_2_(CH_3_COO)_2_, except for nickel­(II) chloride hexahydrate (0.48 g, 2.0 mmol) as the reactants. Yield: 0.98 g (88%). Anal. calc. for C_61_H_48_Cl_2_N_6_Ni_2_O_2_·0.5H_2_O: C, 66.95; H, 4.51; N, 7.86%. Found: C, 67.07; H, 4.92; N, 7.67%. MALDI-TOF-MS (*m*/*z*): 1049.4 {100%, [^*H3C*^LNi_2_Cl]^+^}.

### Synthesis of [^*OMe3C*^LNi_2_Cl_2_] (**6**)

This complex was synthesized as mentioned above for ^*OMe3C*^LNi_2_(CH_3_COO)_2_, except for nickel­(II) chloride hexahydrate (0.48 g, 2.0 mmol) as the reactants. Yield: 0.99 g (86%). Anal. calcd for C_63_H_52_Cl_2_N_6_Ni_2_O_4_: C, 66.06; H, 4.58; N, 7.34%. Found: C, 66.11; H, 5.07; N, 7.32%. MALDI-TOF-MS (*m*/*z*): 1109.5 {100% [^*OMe3C*^LNi_2_Cl]^+^}.

### Synthesis of [^*Cl3C*^LNi_2_Cl_2_] (**7**)

This complex was synthesized as mentioned above for ^*Cl3C*^LNi_2_(CH_3_COO)_2_, except for nickel­(II) chloride hexahydrate (0.48 g, 2.0 mmol) as the reactants. Yield: 0.94 g (82%). Anal. calc. for C_61_H_46_Cl_4_N_6_Ni_2_O_2_·0.5C_6_H_14_: C, 64.20; H, 4.46; N, 7.02%. Found: C, 64.27; H, 4.25; N, 7.36%. MALDI-TOF-MS (*m*/*z*): 1117.3 {100%, [^*Cl3C*^LNi_2_Cl]^+^}.

## Supplementary Material



## Notes

General procedures, experimental details, methods (copolymerization optimization and kinetic studies), and compound or polymer characterization data (MS, NMR, crystallographic data, and GPC plots) (PDF)
