# Supplementary material for: Dinuclear Nickel Complexes Containing Imidazole-Based Bis(iminophenolate) Derivatives: Highly Active Catalysts for Ring-Opening Copolymerization of Carbon Dioxide with Alicyclic Epoxides
Source: Inorg Chem. 2026 Sep 1;65(36):21079–93. doi: 10.1021/acs.inorgchem.6c02993 (PMC13580119; doi:10.1021/acs.inorgchem.6c02993)
Supplement: Supplementary file 1 [file ic6c02993_si_001.pdf]

Supporting Information

Dinuclear Nickel Complexes Containing Imidazole-Based Bis(iminophenolate) Derivatives: Highly Active Catalysts for Ring-Opening Copolymerization of Carbon Dioxide with Alicyclic Epoxides

Jun-Cheng Chen,† Guan-Lin Liu† and Bao-Tsan Ko*

Department of Chemistry, National Chung Hsing University, Taichung 402, Taiwan

†J.-C. Chen and G.-L. Liu contributed equally to this work

*E-mail: btko@dragon.nchu.edu.tw

Experimental Section

Scheme S1. Synthetic Routes of Ligand Precursors

Figure S1. Solid-state molecular structure of complex **3** (50% probability level). All hydrogen atoms have been omitted for clarity.

Figure S2. Solid-state molecular structure of complex **4** (50% probability level). All hydrogen atoms have been omitted for clarity.

Figure S3. The mass spectra and the corresponding simulated isotopic distributions[S4] of dinuclear nickel complexes **1–7**. (a) **1**, (b) **2**, (c) **3**, and (d) **4** were obtained using positive electrospray ionization (ESI^+), whereas (e) **5**, (f) **6**, and (g) **7** were obtained using matrix-assisted laser desorption/ionization time-of-flight mass spectrometry in the positive-ion mode ($MALDI\text{-}TOF^+$).

Figure S4. ^{1}H NMR spectrum (400 MHz, $CDCl_3$) of the purified (a) poly(cyclohexene carbonate) (PCHC, Table 1, entry 6); (b) poly(vinylcyclohexene carbonate) (PVCHC, Table 2, entry 19) produced by using dinuclear nickel complex **6**.

Figure S5. (a) The MALDI-TOF spectrum of the generated PCHC catalyzed by dinuclear nickel complex **6** (Copolymerization conditions: 0.03125 mmol complex **6**, $[CHO]_0/[\mathbf{6}]_0$ = 5,000/1, pCO_2^0 = 20.7 bar, 120 ºC, 40 min). (b) The selected fragments of mass-to-charge (m/z) ranging from 6,000 to 6,600.

Figure S6. Representative GPC traces for the generated PCHC mediated by dinuclear nickel (a) complex **2** (Table 2, entry 3); (b) complex **6** (Table 2, entry 8).

Figure S7. ^{1}H NMR spectrum (400 MHz, $CDCl_3$) of purified PCHC-*co*-PVCHC obtained from terpolymerization at different $[CHO]_0/[VCHO]_0$ ratios using dinickel complex **6**. (a) $[CHO]_0/[VCHO]_0$ = 80/20, n : m = 71 : 29 (Table 3, entry 1), (b) $[CHO]_0/[VCHO]_0$ = 20/80, n : m = 16 : 84 (Table 3, entry 2).

Figure S8. ^{1}H NMR spectrum (400 MHz, $CDCl_3$) of purified PCHC-*co*-PAMCHC obtained from terpolymerization using dinickel complex **6** (Table 3, entry 4, n : m = 84 : 16).

Figure S9. The (a) ^{1}H (600 MHz) and (b) ^{13}C (151 MHz) NMR spectrum of the ^{OMe}TPImP-H (in $CDCl_3$).

Figure S10. The (a) ^{1}H (600 MHz) and (b) ^{13}C (151 MHz) NMR spectrum of the ^{Cl}TPImP-H (in $CDCl_3$).

Figure S11. The (a) ^{1}H (600 MHz) and (b) ^{13}C (151 MHz) NMR spectrum of the ^{OMeAld}TPImP-H (in $CDCl_3$).

Figure S12. The (a) ^{1}H (600 MHz) and (b) ^{13}C (151 MHz) NMR spectrum of the ^{ClAld}TPImP-H (in $CDCl_3$).

Figure S13. The (a) ^1H (600 MHz) and (b) ^{13}C (151 MHz) NMR spectrum of the ^{H3C}L-H_2 (in $CDCl_3$).

Figure S14. The (a) ^1H (600 MHz) and (b) ^{13}C (151 MHz) NMR spectrum of the ^{OMe3C}L-H_2 (in $CDCl_3$).

Figure S15. The (a) ^1H (600 MHz) and (b) ^{13}C (151 MHz) NMR spectrum of the ^{Cl3C}L-H_2 (in $CDCl_3$).

Figure S16. The (a) ^1H (600 MHz) and (b) ^{13}C (151 MHz) NMR spectrum of the ^{OMe4C}L-H_2 (in $CDCl_3$).

Table S1. Crystallographic Data of Dinickel Complexes **1**, **3**, **4** and **5'**

Table S2. Selected Average Bond Distances and Bond Angles of Dinickel Complexes **1**, **3**, **4** and **5'**

Table S3. Copolymerization of CO_2 and CHO Catalyzed by Dinuclear Nickel Complex **6** Under Different Initial CO_2 Pressures for 1 h[a]

Table S4. Kinetic Studies of CHO/CO_2 Copolymerization Mediated by Dinuclear Nickel Complex **6** at Fixed Monomer-to-Catalyst Ratios[a]

Table S5. Kinetic Parameters of CO_2/CHO Copolymerization Mediated by Dinuclear Nickel Complex **6** at Different CHO-to-Catalyst Ratios

Experimental Section

Deuterated chloroform ($CDCl_3$) and benzene (C_6D_6) solvents were dried over 4 Å molecular sieves. 2-Hydroxy-5-methylbenzaldehyde, benzil, aniline, p-anisidine, 4-chloroaniline, ammonium acetate, acetic acid, hexamethylenetetramine , trifluoroacetic acid, 1,3-diaminopropane, 1,4-diaminobutane, nickel(II) acetate tetrahydrate, nickel(II) chloride hexahydrate, triethylamine (NEt_3), methanol (MeOH), dichloromethane (CH_2Cl_2), acetone, diethyl ether, *n*-hexane, and carbon dioxide (CO_2, 99.95%) were purchased and used without further purification. Cyclohexene Oxide (CHO), 4-vinyl-1,2-epoxycyclohexane (VCHO), and Cyclopentene Oxide (CPO) were purified by distillation over calcium hydride (CaH_2) at least four times prior to use. ^{1}H NMR and ^{13}C NMR spectra were recorded on a Varian Mercury-400 (400 and 100 MHz) spectrometer with chemical shifts given in parts per million from the peak of internal tetramethylsilane (TMS). Microanalyses were performed using a Heraeus CHN-O-RAPID instrument. Mass analyses were performed by utilizing the positive electron spray ionization (ESI^+) technique with a Thermo Finnigan TSQ Quantum mass spectrometer for newly prepared complexes upon dissolving in CH_2Cl_2 solvent. GPC measurements were performed using a Jasco PU-2080 plus system equipped with a RI-2031 detector using THF (HPLC grade) as the eluent. The chromatographic column was Phenomenex Phenogel 5 μ 103 Å, and the calibration curve utilized to calculate Mn(GPC) was produced from polystyrene standards. The calibration curve was constructed of 10 polystyrene standards, in which their molecular weights range from 1580 to 288,000 g mol^{-1}. The GPC results were calculated using the Scientific Information Service Corporation (SISC) chromatography data solution 3.1 edition. The MALDI-TOF spectra for polymer characterization were measured with an

Ultraflex III TOF mass spectrometer. 2,5-Dihydroxybenzoic acid was utilized as the matrix. The samples were prepared by mixing 20 mg/mL THF solution of PCHC and the matrix (1 μL) together. This mixture (1 μL) was then spotted into a stainless-steel MALDI target plate (MTP 384 target plate) and allowed to dry at room temperature.

ROCOP of CO_2 with CHO Catalyzed by Dinickel Complexes

The CO_2-copolymerization of CHO by complex **2** was exemplified as a representative procedure (Table 1, entry 2). [^{OMe3C}LNi$_2$(CH$_3$COO)$_2$] (**2**) (37.3 mg, 0.03125 mmol) was dissolved in 100.0 mmol of neat CHO in a dry nitrogen atmosphere. The mixture was added to a 100 mL autoclave with a magnetic stir bar under a CO_2 atmosphere. The reactor was then charged with CO_2 until a pressure of 20.7 bar was reached, and the stirrer was started. The copolymerization was carried out at 120 °C for 2 h. After cooling with ice water and venting the reactor, a small aliquot of the copolymerization mixture was taken for ^{1}H NMR spectroscopic analysis. The CHO conversion (56%) and the copolymerization selectivity (>99% PCHC; >99% carbonate linkages) were characterized by ^{1}H NMR spectroscopy. The remaining crude was further dissolved in 5 mL of CH_2Cl_2 and precipitated in excess MeOH to give yellowish solids. In order to remove metal salts, the resulting copolymer was diluted with 20 mL of CH_2Cl_2 and washed with aqueous HCl (4 N, 100 mL). The organic layer was then extracted and concentrated to 5 mL by vacuum evaporation. Finally, the copolymer was precipitated into MeOH (150 mL) again to yield a white solid.

X-ray crystallographic studies

Suitable crystals of bimetallic nickel complexes **1**, **3**, **4** and **5'** were mounted onto glass fiber by utilizing perfluoropolyether oil and cooled rapidly in a stream of cold

nitrogen gas to collect diffraction data using a Bruker APEX2 diffractometer (for complexes **1**, **3** and **4**) and Rigaku XtaLAB Synergy DW (for complex **5'**). The space group determination was based on a check of the Laue symmetry and systematic absences and was verified by utilizing the structure solution. The structure was solved and refined with the SHELXTL package.[S1] All non-H atoms were located from successive Fourier maps, and hydrogen atoms were treated as a riding model on their parent C atoms. Anisotropic thermal parameters were used for all non-H atoms, and fixed isotropic parameters were used for H-atoms. Drawing of the molecular structure was performed by using Oak Ridge Thermal Ellipsoid Plots (ORTEP).[S2] Crystallographic data of dinuclear nickel complexes **1**, **3**, **4** and **5'** are shown in Tables S1 and S2. In addition, all crystallographic data generally revealed the presence of highly disordered solvent molecules, thus the SQUEEZE subroutine in the PLATON program (for complexes **4** and **5'**) was applied to data refinements.[S3] According to the solvent mask results calculated by the crystallographic program, the masked molecule types and quantities were added to the formula and presented in Tables S1 and S2.

Scheme S1. Synthetic Routes of Ligand Precursors

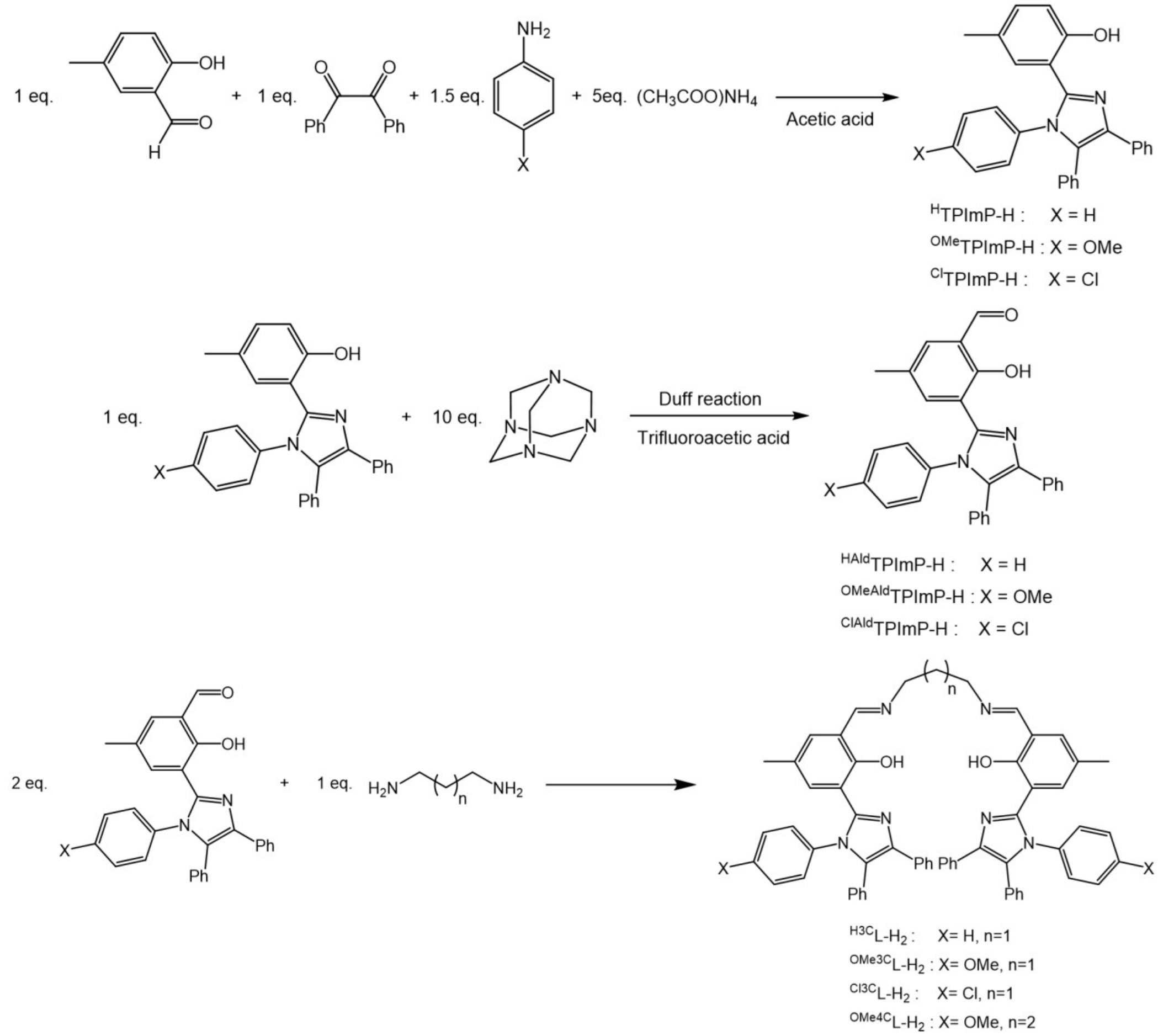

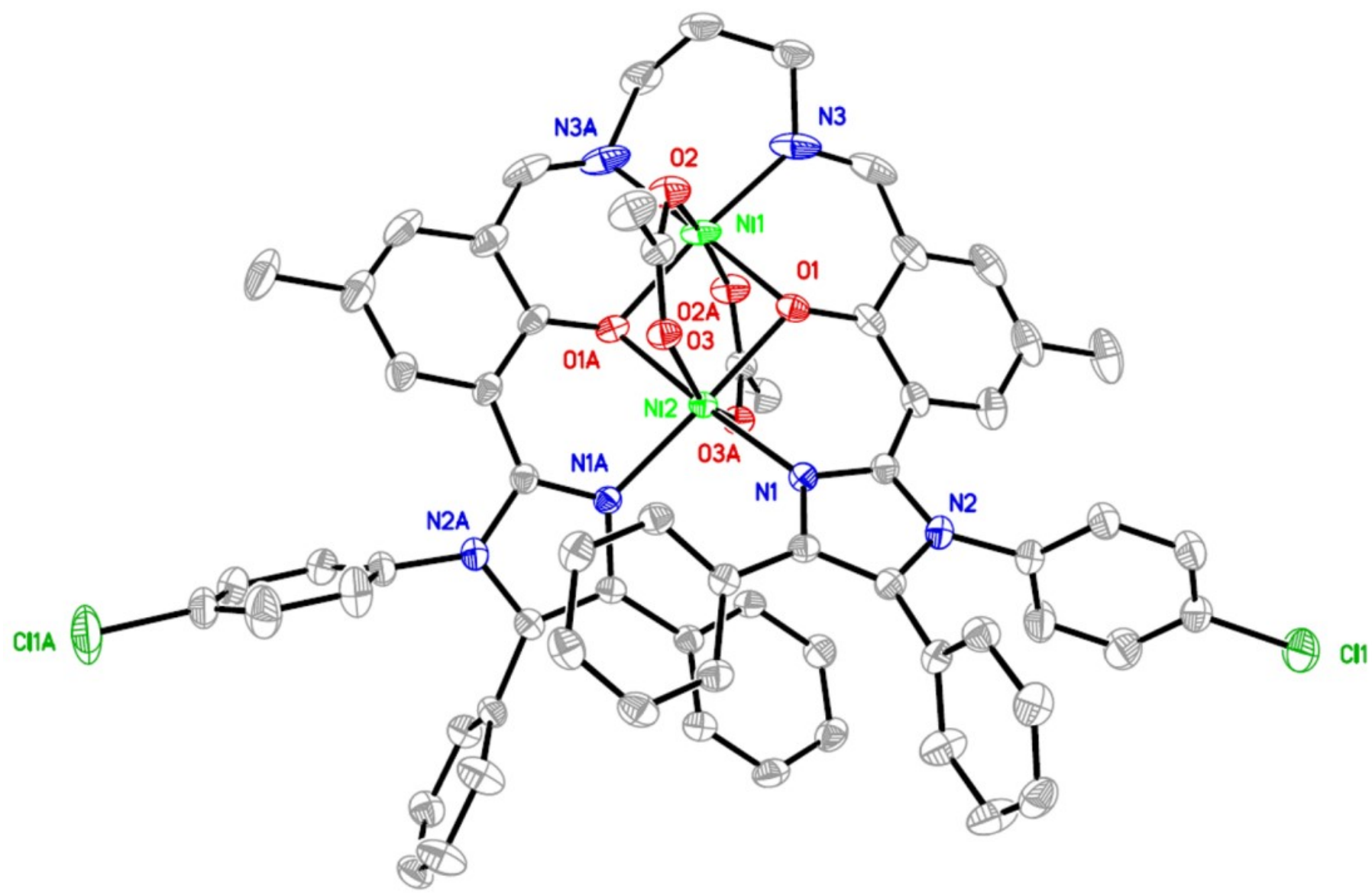

Figure S1. Solid-state molecular structure of complex **3** (50% probability level). All hydrogen atoms have been omitted for clarity.

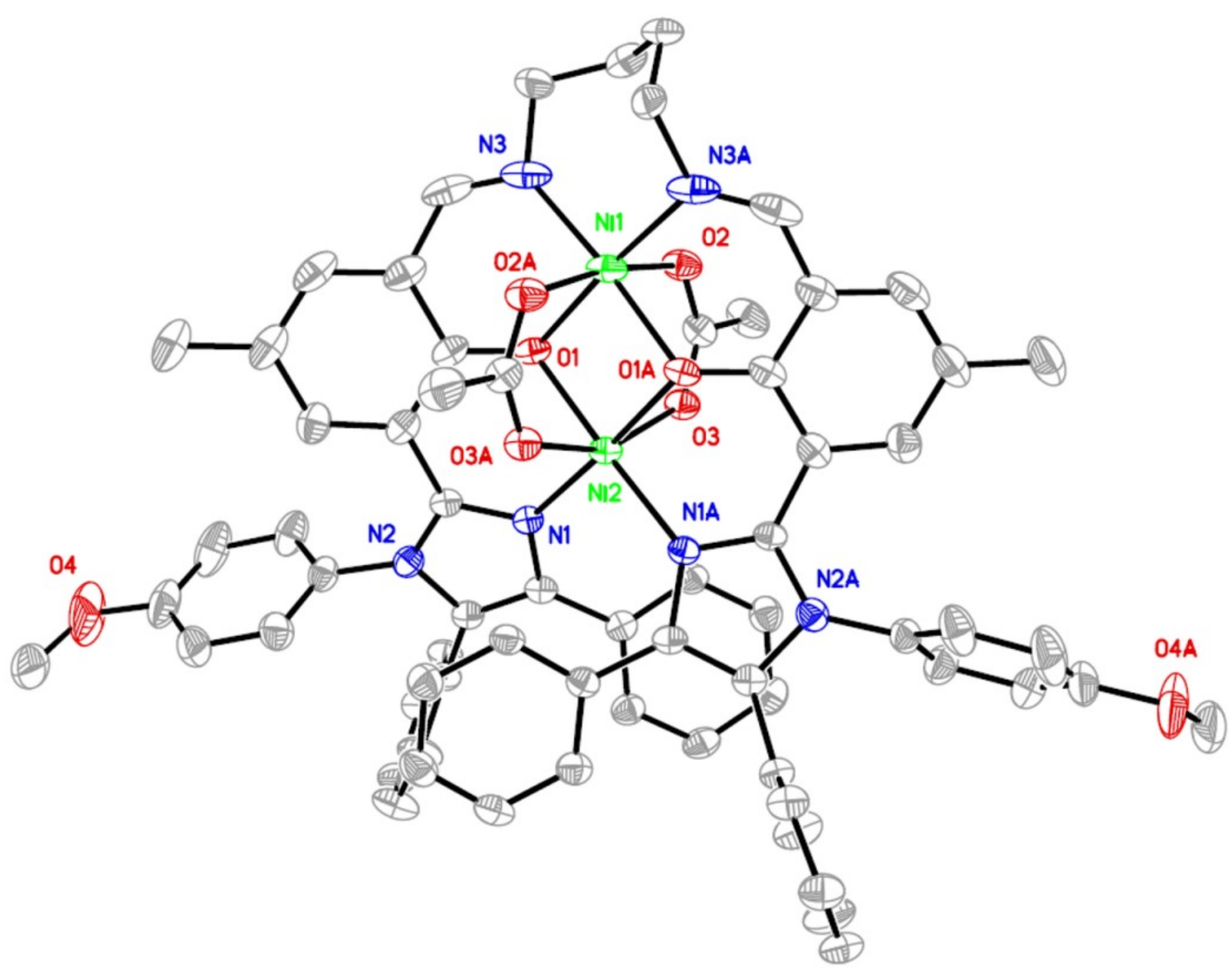

Figure S2. Solid-state molecular structure of complex **4** (50% probability level). All hydrogen atoms have been omitted for clarity.

(a)

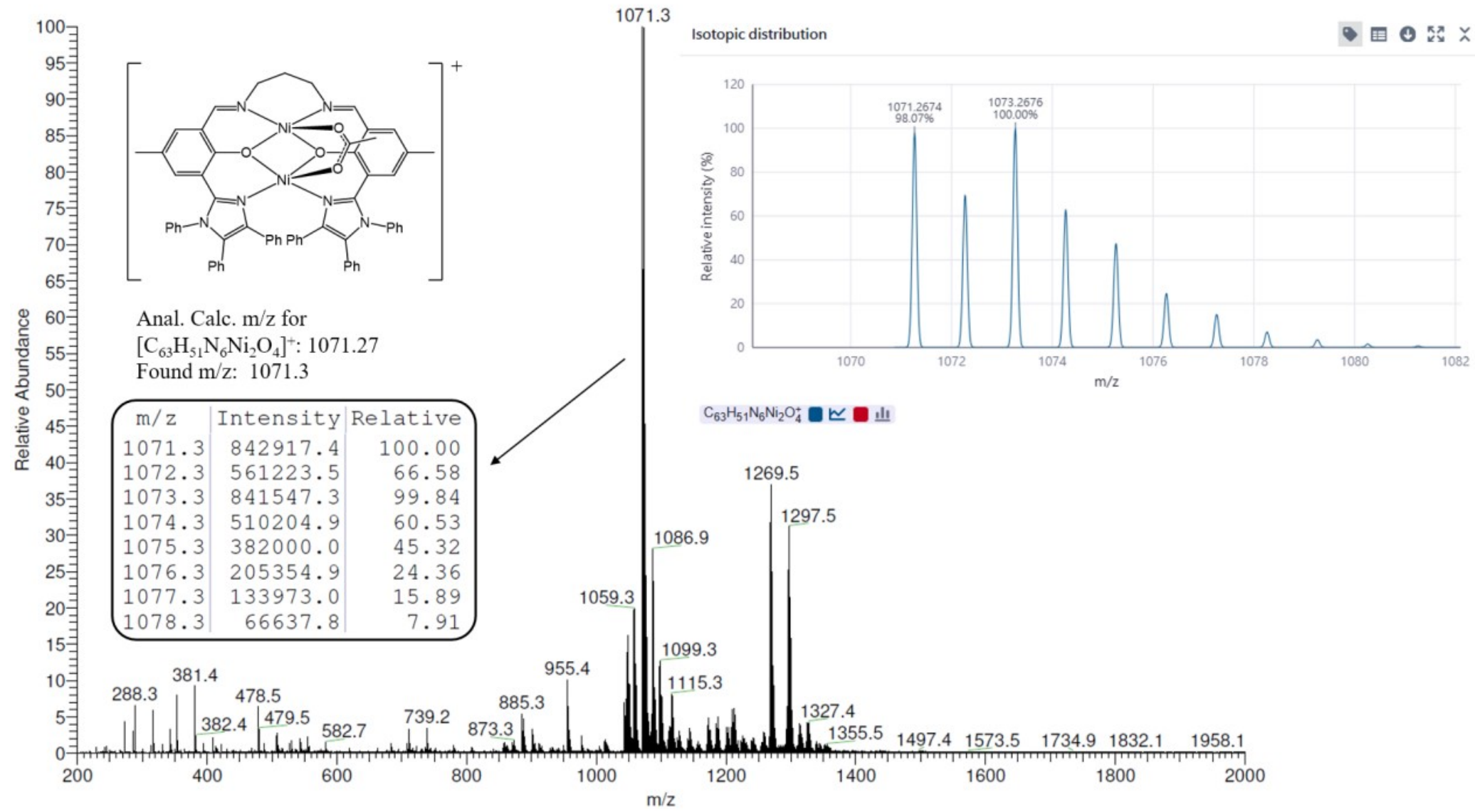

Anal. Calc. m/z for
$[C_{63}H_{51}N_6Ni_2O_4]^+$: 1071.27
Found m/z: 1071.3
m/z Intensity Relative
1071.3 842917.4 100.00
1072.3 561223.5 66.58
1073.3 841547.3 99.84
1074.3 510204.9 60.53
1075.3 382000.0 45.32
1076.3 205354.9 24.36
1077.3 133973.0 15.89
1078.3 66637.8 7.91
Isotopic distribution
1071.2674 98.07%
1073.2676 100.00%
Relative Abundance
m/z

(b)

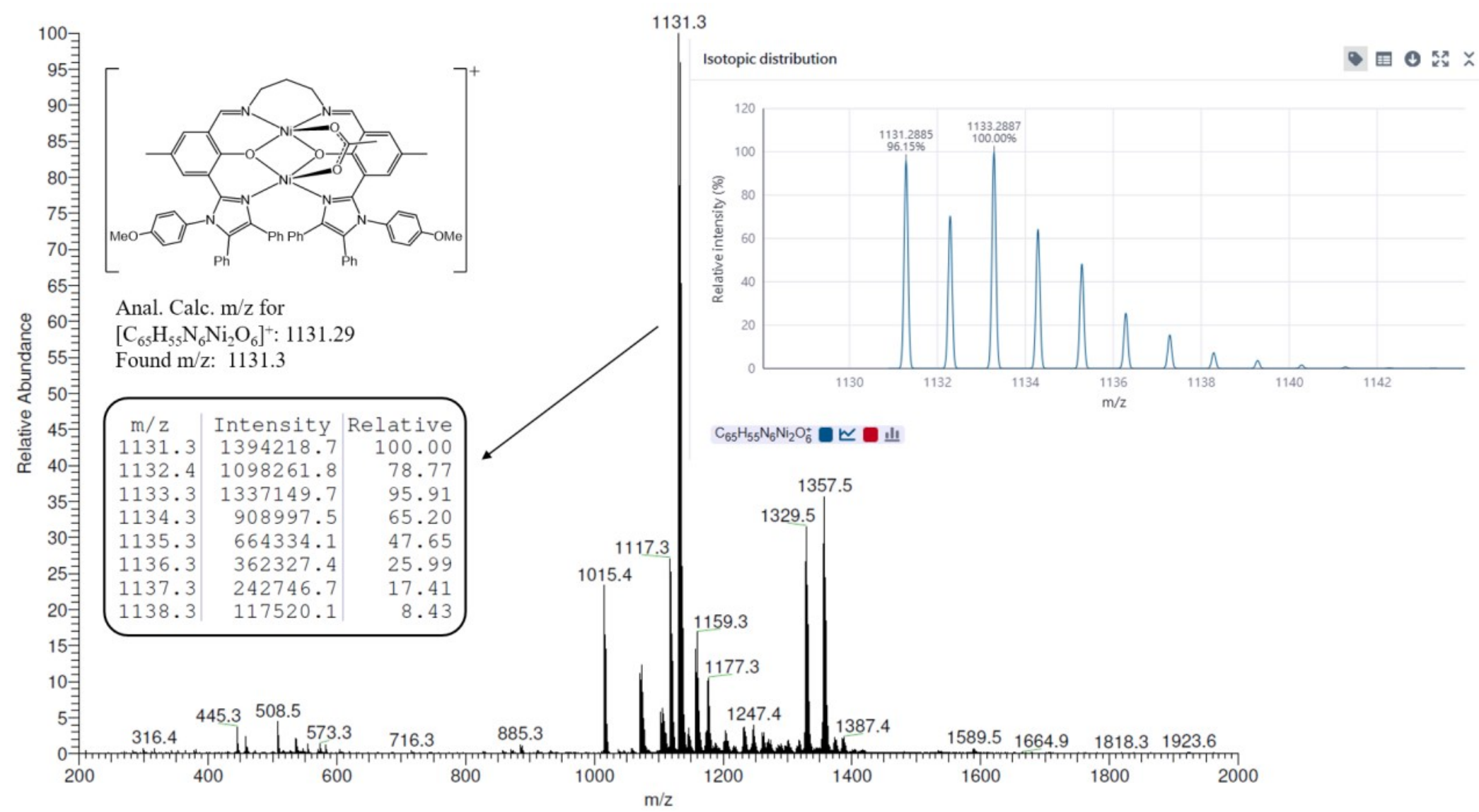

Anal. Calc. m/z for
$[C_{65}H_{55}N_6Ni_2O_6]^+$: 1131.29
Found m/z: 1131.3
m/z Intensity Relative
1131.3 1394218.7 100.00
1132.4 1098261.8 78.77
1133.3 1337149.7 95.91
1134.3 908997.5 65.20
1135.3 664334.1 47.65
1136.3 362327.4 25.99
1137.3 242746.7 17.41
1138.3 117520.1 8.43
Isotopic distribution
1131.2885 96.15%
1133.2887 100.00%
Relative Abundance
m/z

(c)

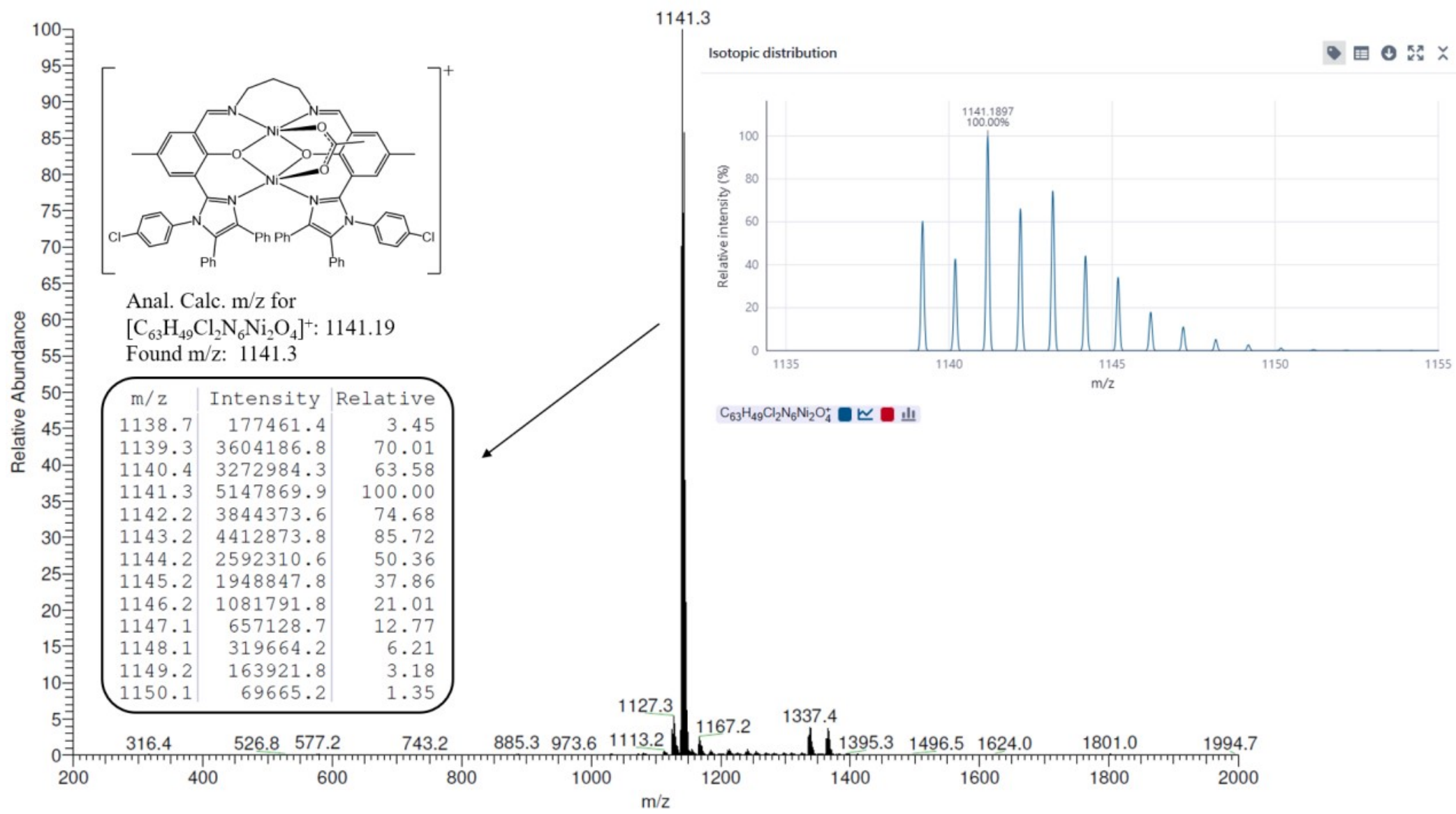

Anal. Calc. m/z for
$[C_{63}H_{49}Cl_2N_6Ni_2O_4]^+$: 1141.19
Found m/z: 1141.3
m/z | Intensity | Relative
1138.7 177461.4 3.45
1139.3 3604186.8 70.01
1140.4 3272984.3 63.58
1141.3 5147869.9 100.00
1142.2 3844373.6 74.68
1143.2 4412873.8 85.72
1144.2 2592310.6 50.36
1145.2 1948847.8 37.86
1146.2 1081791.8 21.01
1147.1 657128.7 12.77
1148.1 319664.2 6.21
1149.2 163921.8 3.18
1150.1 69665.2 1.35
Relative Abundance
m/z
1141.3
1127.3
1167.2
1337.4
Isotopic distribution
1141.1897
100.00%
Relative intensity (%)
$C_{63}H_{49}Cl_2N_6Ni_2O_4^+$

(d)

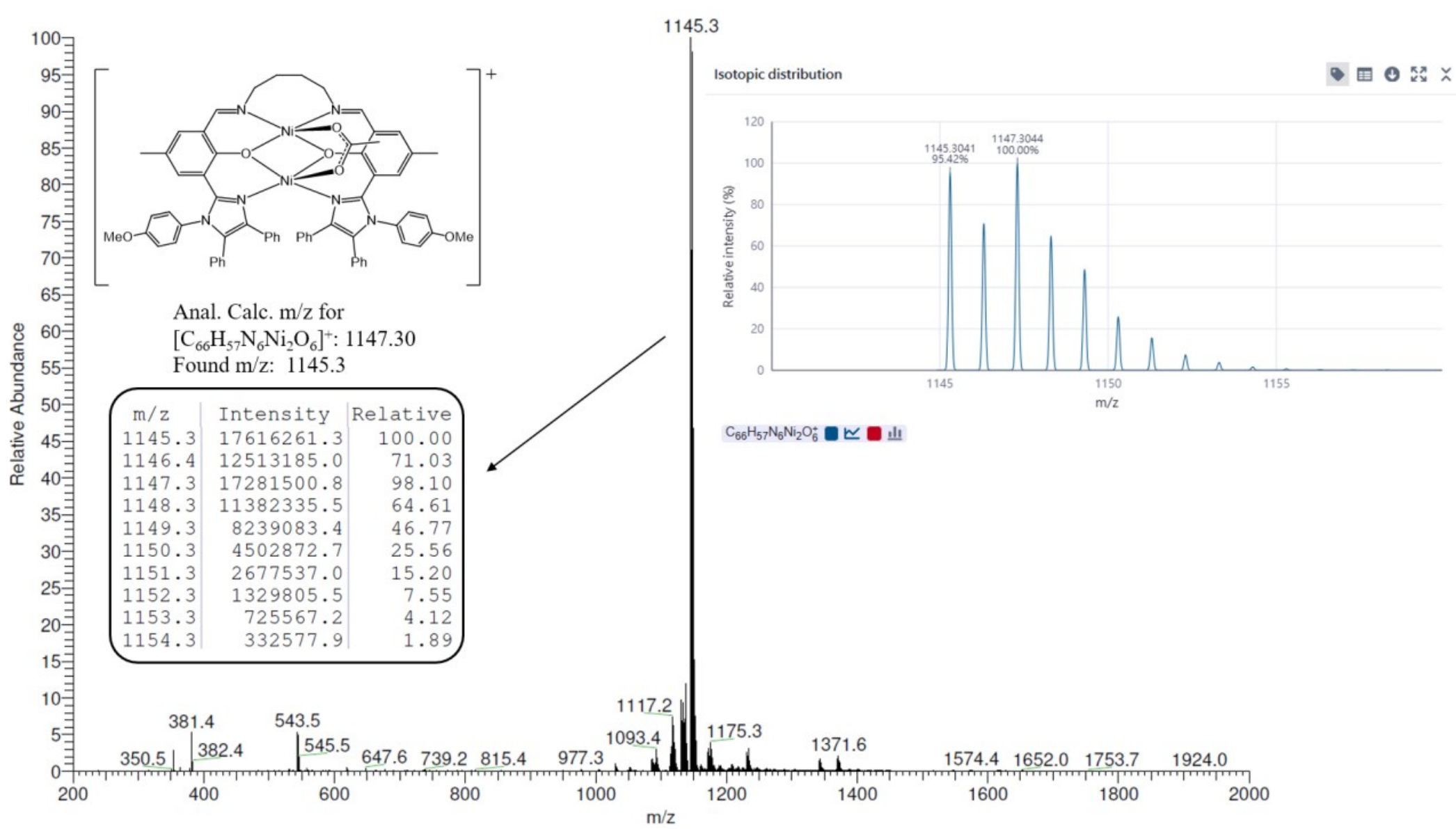

Anal. Calc. m/z for
$[C_{66}H_{57}N_6Ni_2O_6]^+$: 1147.30
Found m/z: 1145.3
m/z | Intensity | Relative
1145.3 17616261.3 100.00
1146.4 12513185.0 71.03
1147.3 17281500.8 98.10
1148.3 11382335.5 64.61
1149.3 8239083.4 46.77
1150.3 4502872.7 25.56
1151.3 2677537.0 15.20
1152.3 1329805.5 7.55
1153.3 725567.2 4.12
1154.3 332577.9 1.89
Relative Abundance
m/z
1145.3
1117.2
1093.4
1175.3
1371.6
Isotopic distribution
1145.3041
95.42%
1147.3044
100.00%
Relative intensity (%)
$C_{66}H_{57}N_6Ni_2O_6^+$

(e)

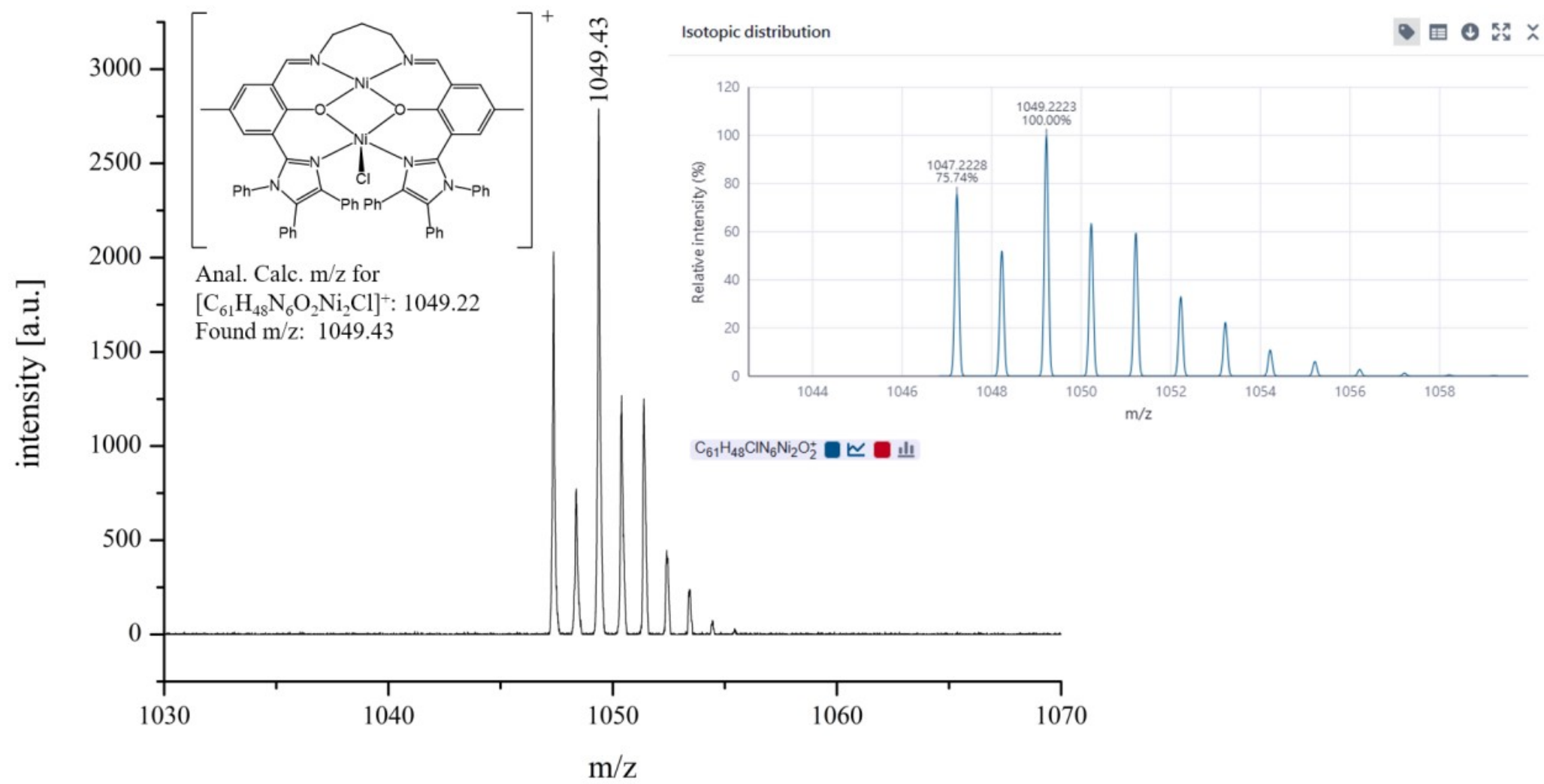
1049.43
Anal. Calc. m/z for
[C61H48N6O2Ni2Cl]+: 1049.22
Found m/z: 1049.43
intensity [a.u.]
m/z
Isotopic distribution
1047.2228
75.74%
1049.2223
100.00%
Relative intensity (%)
m/z
C61H48ClN6Ni2O2+

(f)

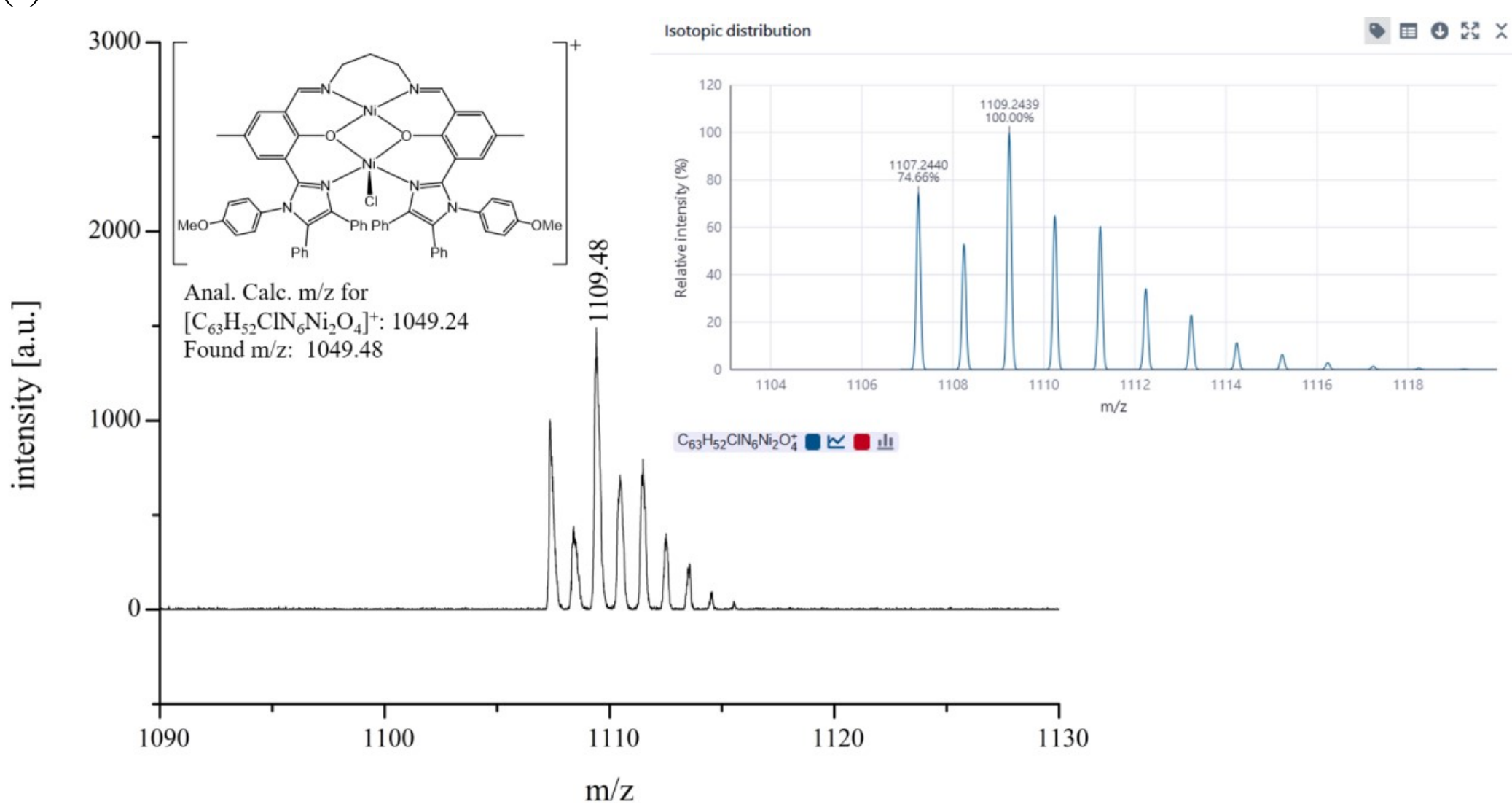
1109.48
Anal. Calc. m/z for
[C63H52ClN6Ni2O4]+: 1049.24
Found m/z: 1049.48
intensity [a.u.]
m/z
Isotopic distribution
1107.2440
74.66%
1109.2439
100.00%
Relative intensity (%)
m/z
C63H52ClN6Ni2O4+

(g)

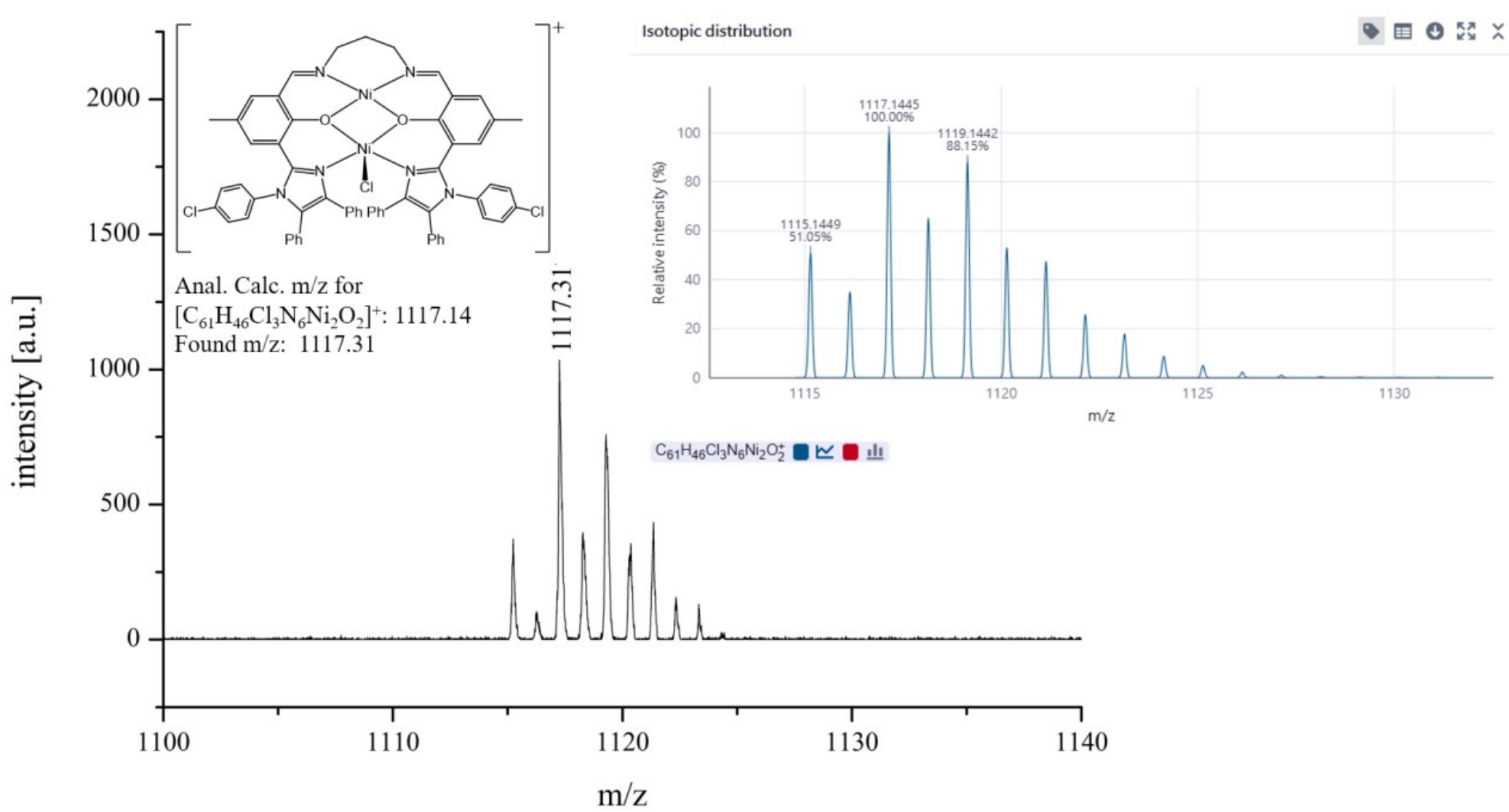

Figure S3. The mass spectra and the corresponding simulated isotopic distributions[S4] of dinuclear nickel complexes **1**–**7**. (a) **1**, (b) **2**, (c) **3**, and (d) **4** were obtained using positive electrospray ionization (ESI^+), whereas (e) **5**, (f) **6**, and (g) **7** were obtained using matrix-assisted laser desorption/ionization time-of-flight mass spectrometry in the positive-ion mode ($MALDI\text{-}TOF^+$).

(a)

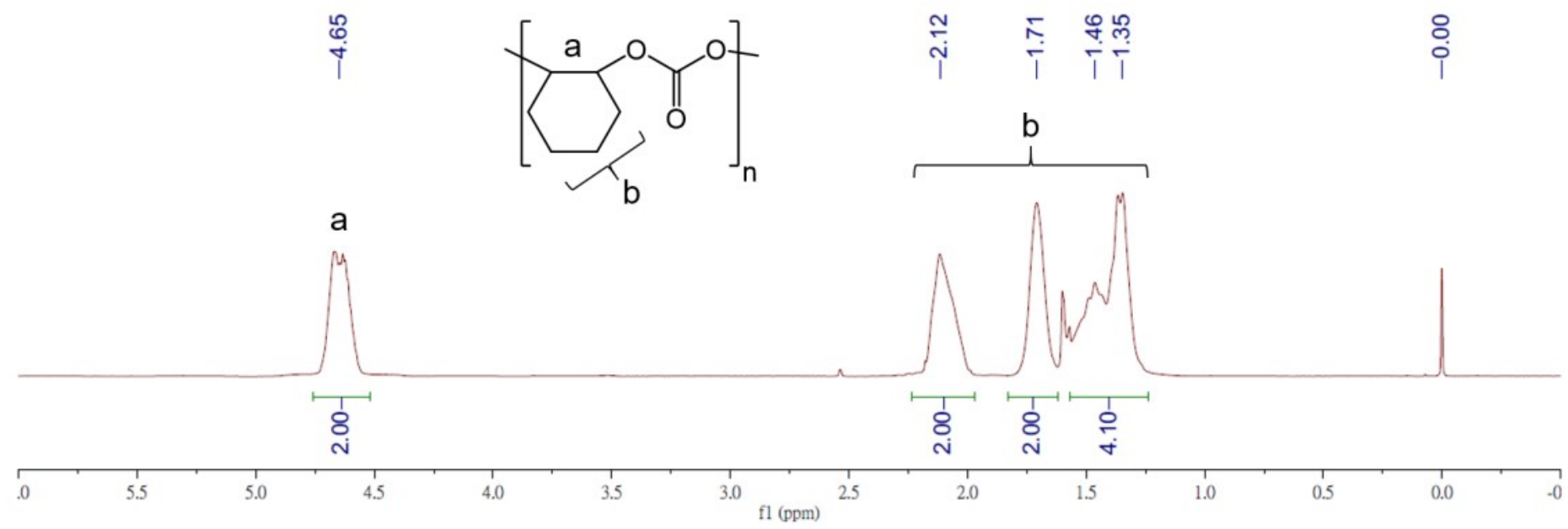

(b)

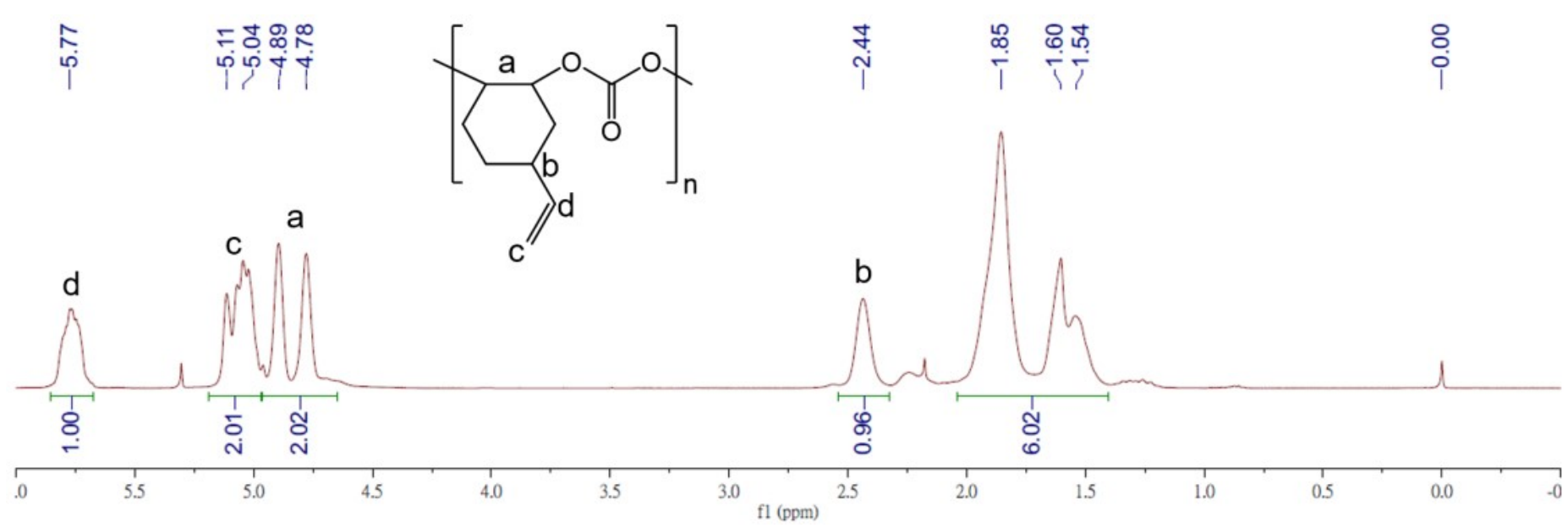

Figure S4. ^{1}H NMR spectrum (400 MHz, $CDCl_3$) of the purified (a) poly(cyclohexene carbonate) (PCHC, Table 1, entry 6); (b) poly(vinylcyclohexene carbonate) (PVCHC, Table 2, entry 19) produced by using dinuclear nickel complex **6**.

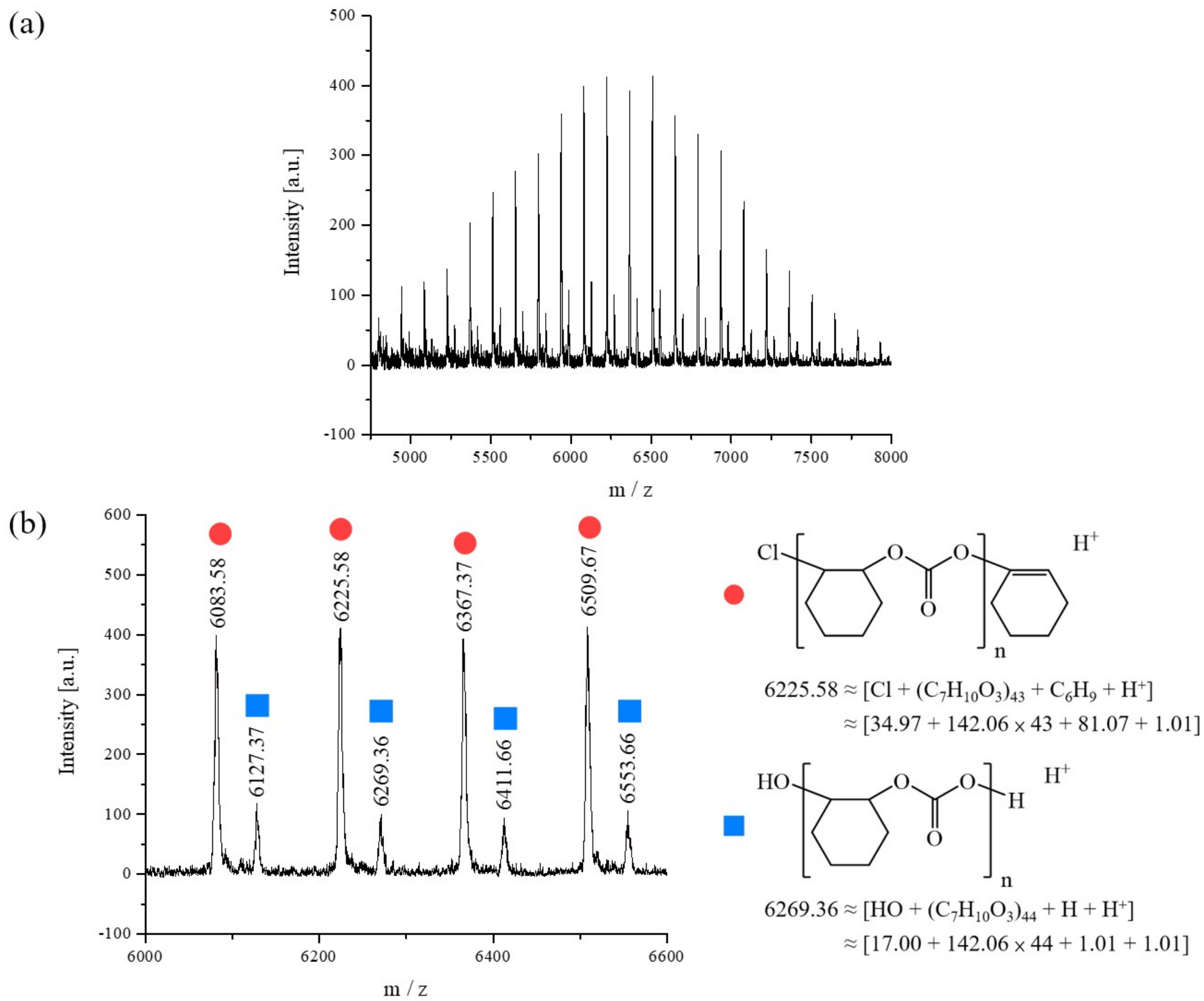

Figure S5. (a) The MALDI-TOF spectrum of the generated PCHC catalyzed by dinuclear nickel complex **6** (Copolymerization conditions: 0.03125 mmol complex **6**, $[CHO]_0/[\mathbf{6}]_0$ = 5,000/1, pCO_2^0 = 20.7 bar, 120 ºC, 40 min). (b) The selected fragments of mass-to-charge (m/z) ranging from 6,000 to 6,600.

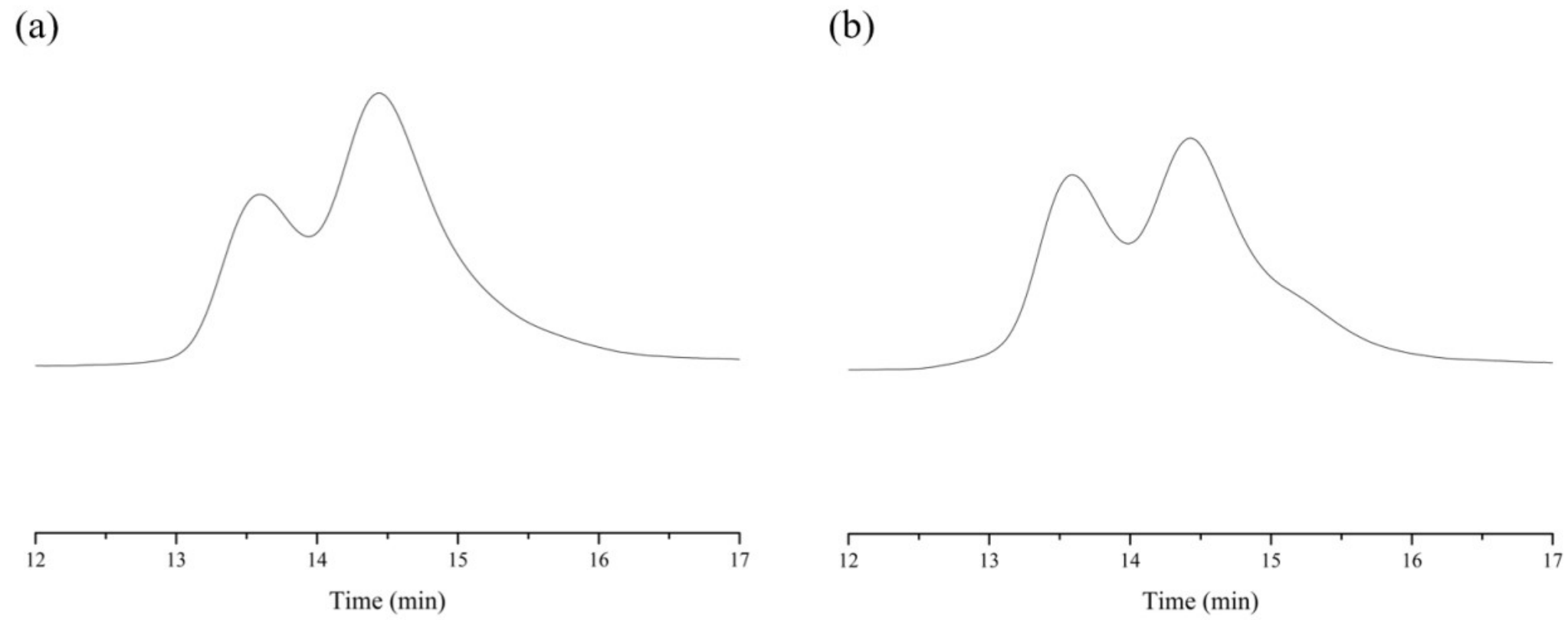

Figure S6. Representative GPC traces for the generated PCHC mediated by dinuclear nickel (a) complex **2** (Table 2, entry 3); (b) complex **6** (Table 2, entry 8).

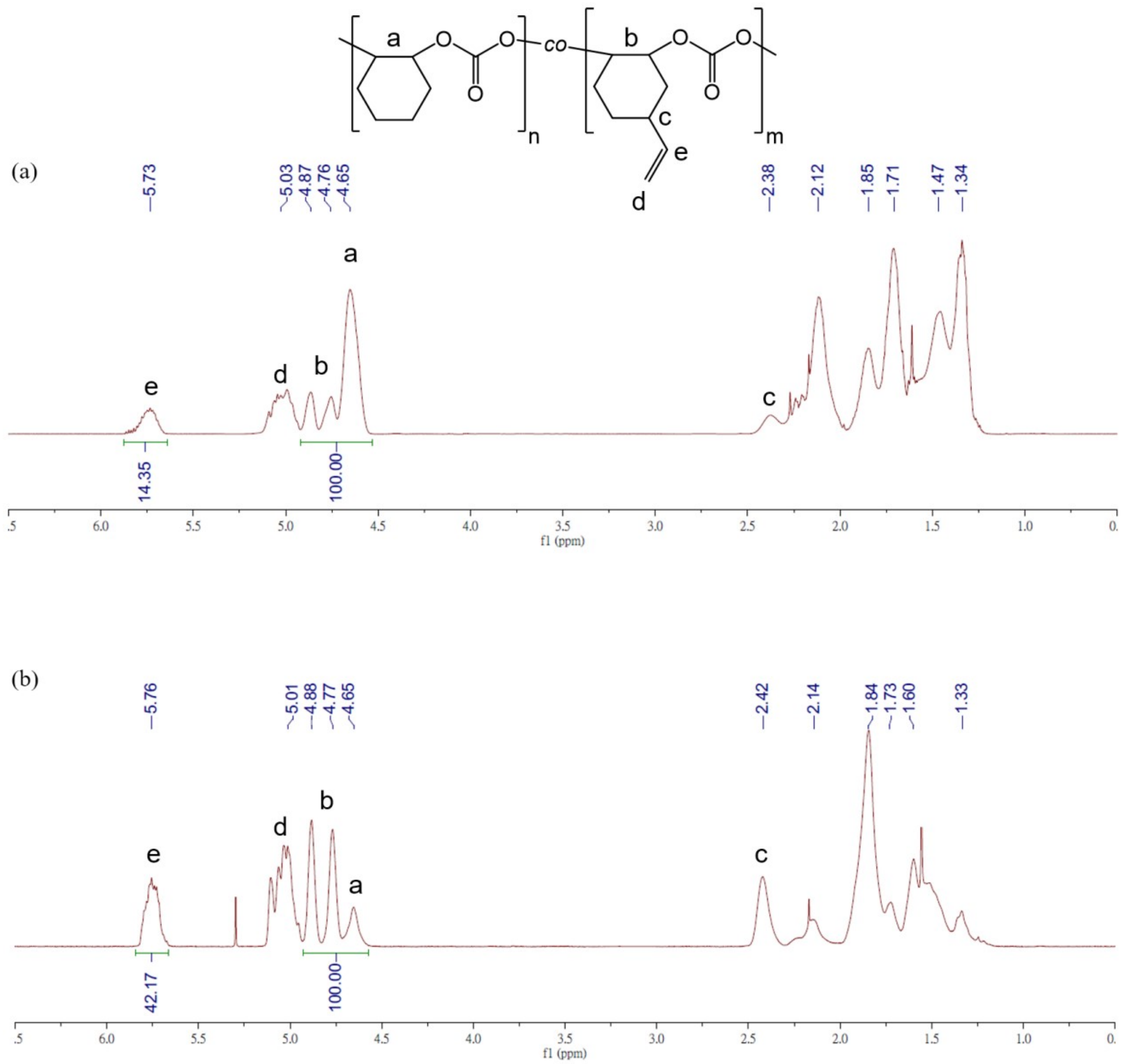

Figure S7. ^{1}H NMR spectrum (400 MHz, $CDCl_3$) of purified PCHC-*co*-PVCHC obtained from terpolymerization at different $[CHO]_0/[VCHO]_0$ ratios using dinickel complex **6**. (a) $[CHO]_0/[VCHO]_0$ = 80/20, n : m = 71 : 29 (Table 3, entry 1), (b) $[CHO]_0/[VCHO]_0$ = 20/80, n : m = 16 : 84 (Table 3, entry 2).

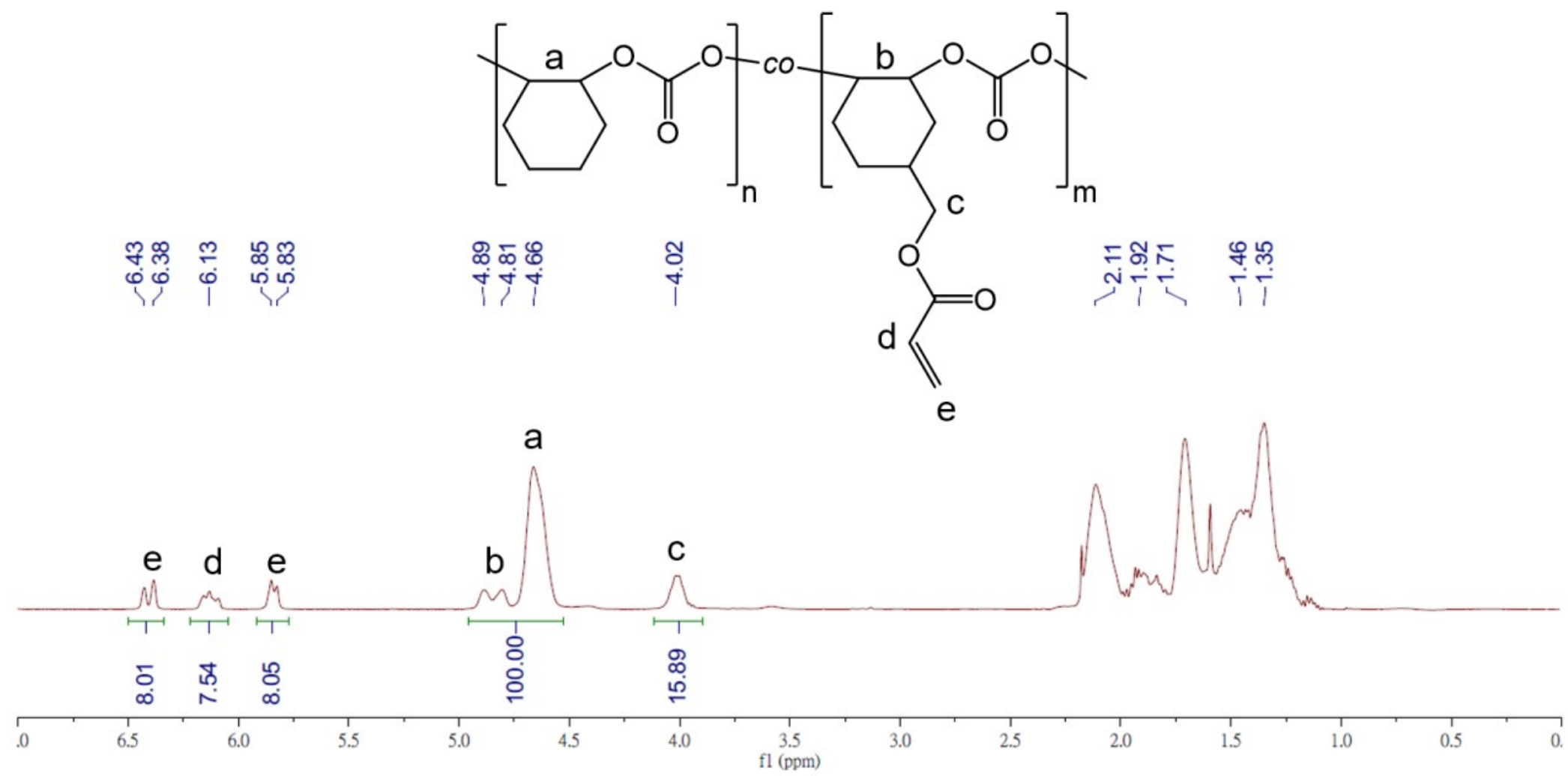

Figure S8. ^{1}H NMR spectrum (400 MHz, $CDCl_3$) of purified PCHC-*co*-PAMCHC obtained from terpolymerization using dinickel complex **6** (Table 3, entry 4, n : m = 84 : 16).

(a)

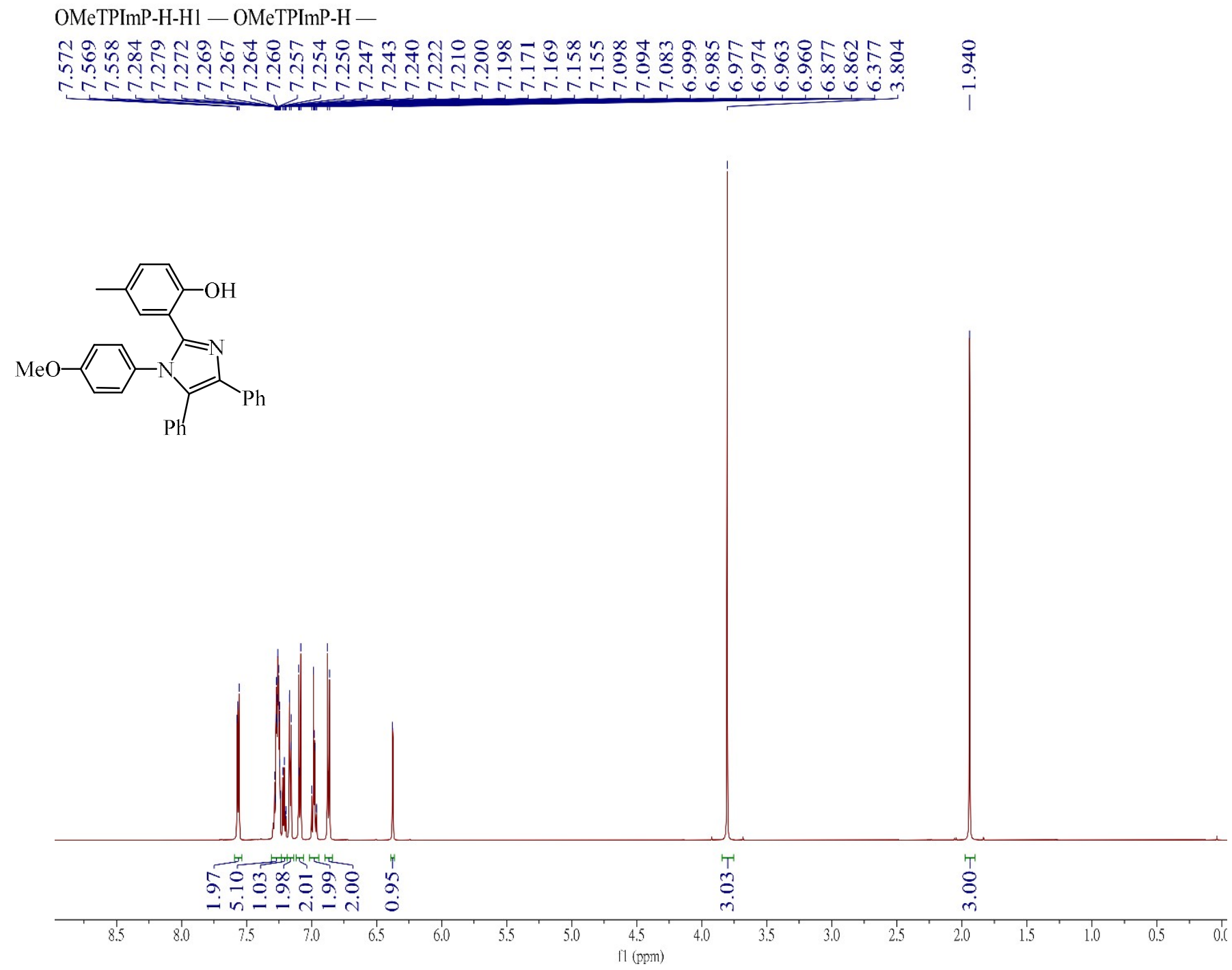

(b)

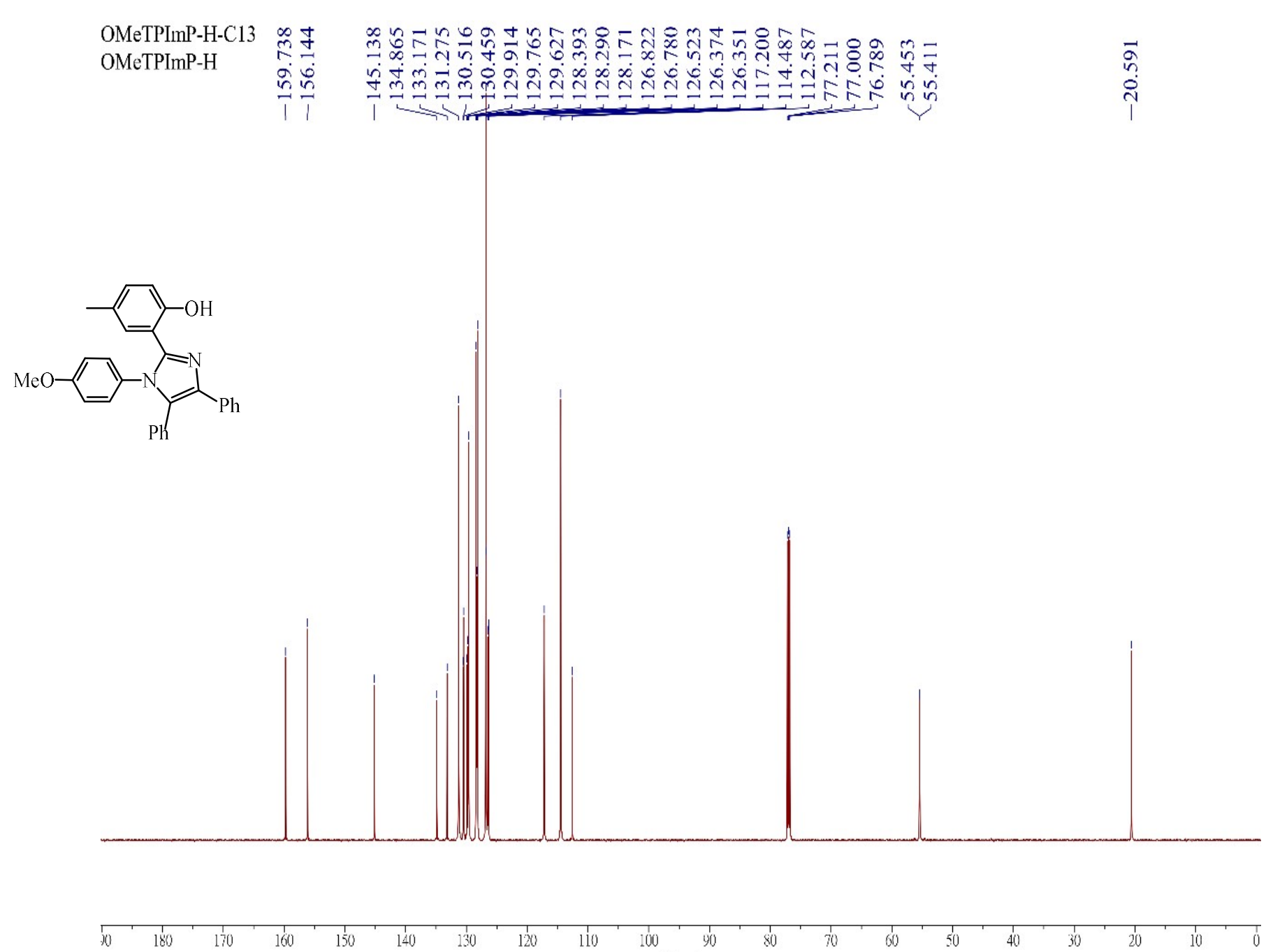

Figure S9. The (a) ^{1}H (600 MHz) and (b) ^{13}C (151 MHz) NMR spectrum of the ^{OMe}TPImP-H (in $CDCl_3$).

(a)

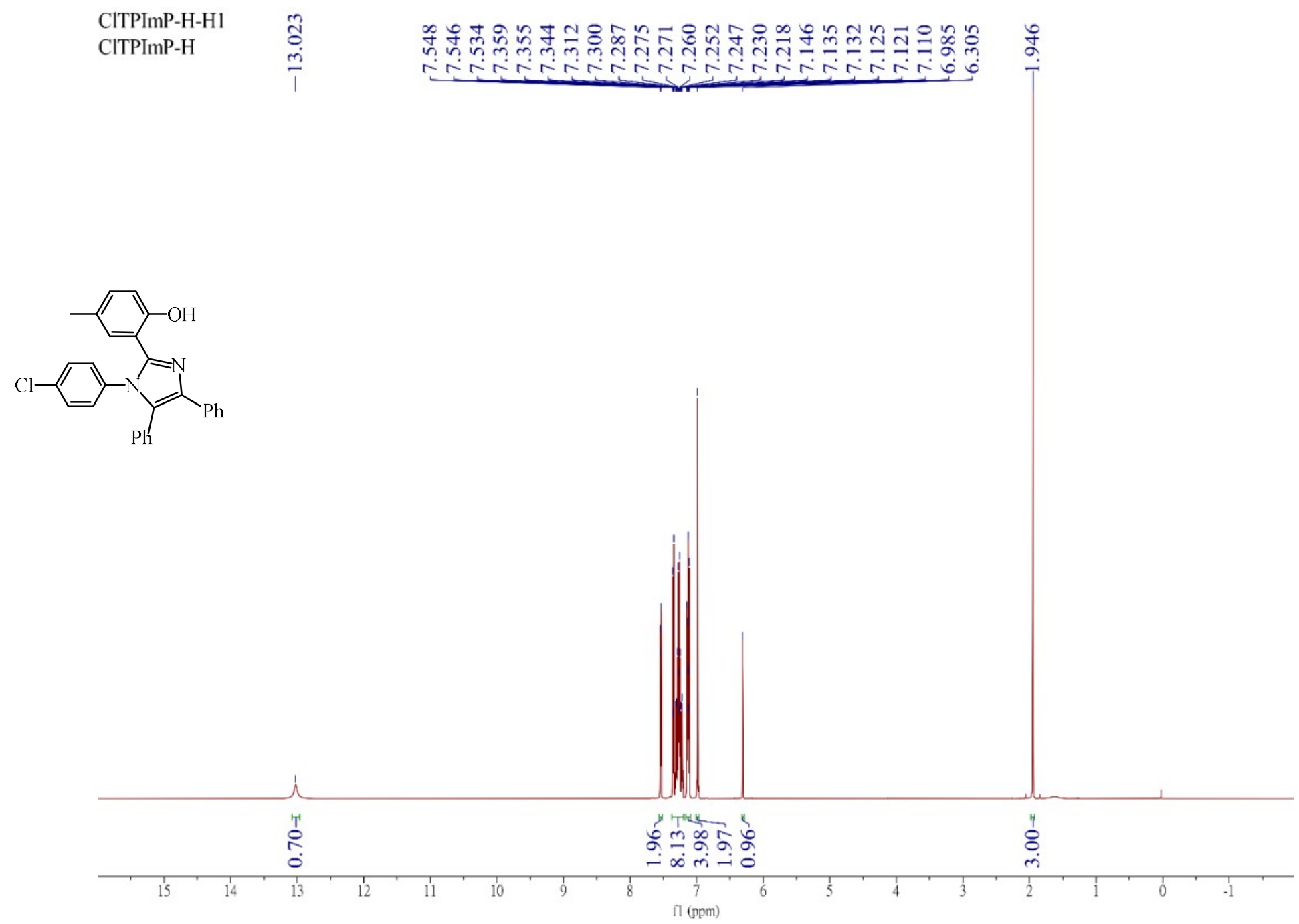

(b)

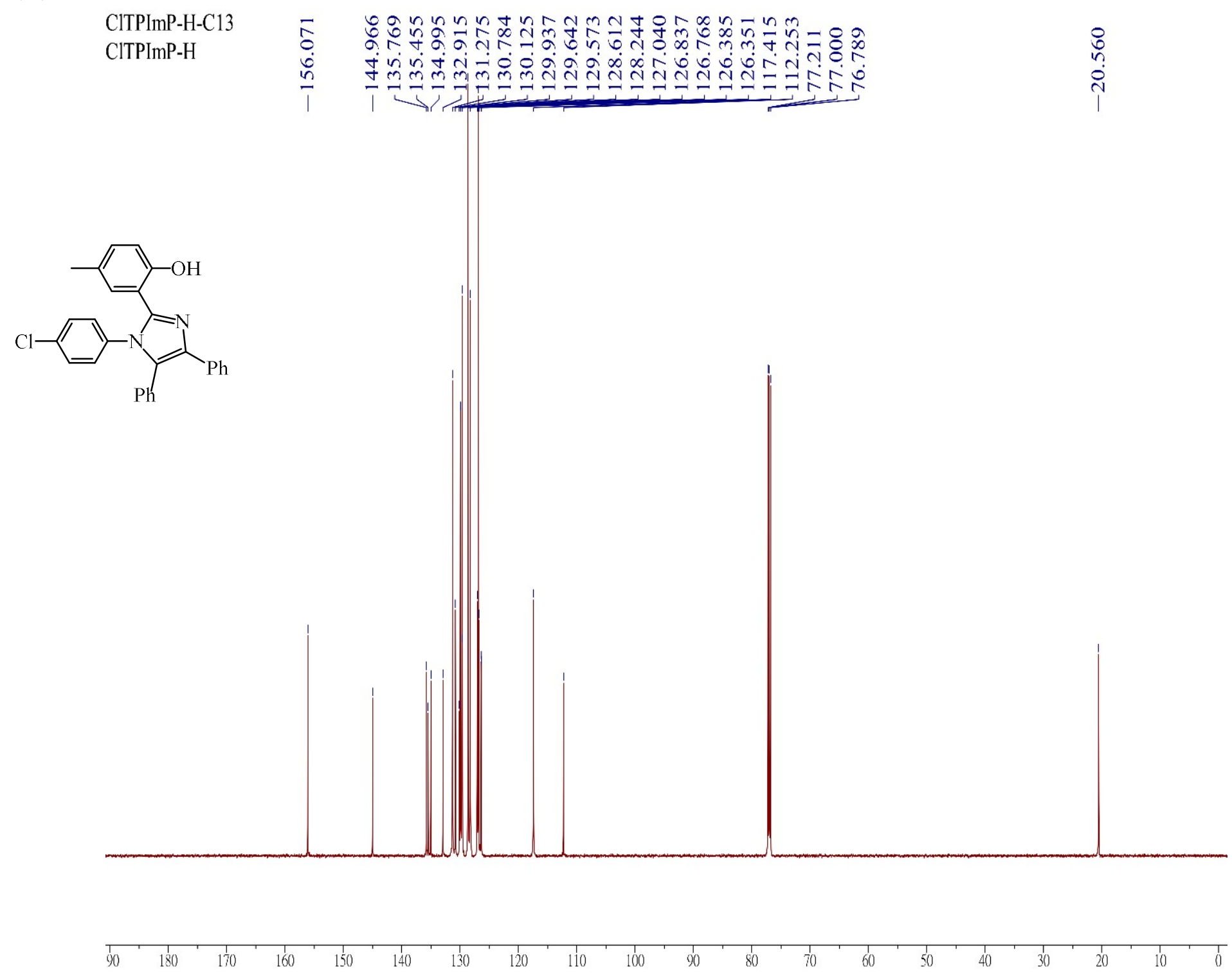

Figure S10. The (a) ^{1}H (600 MHz) and (b) ^{13}C (151 MHz) NMR spectrum of the ^{Cl}TPImP-H (in $CDCl_3$).

(a)

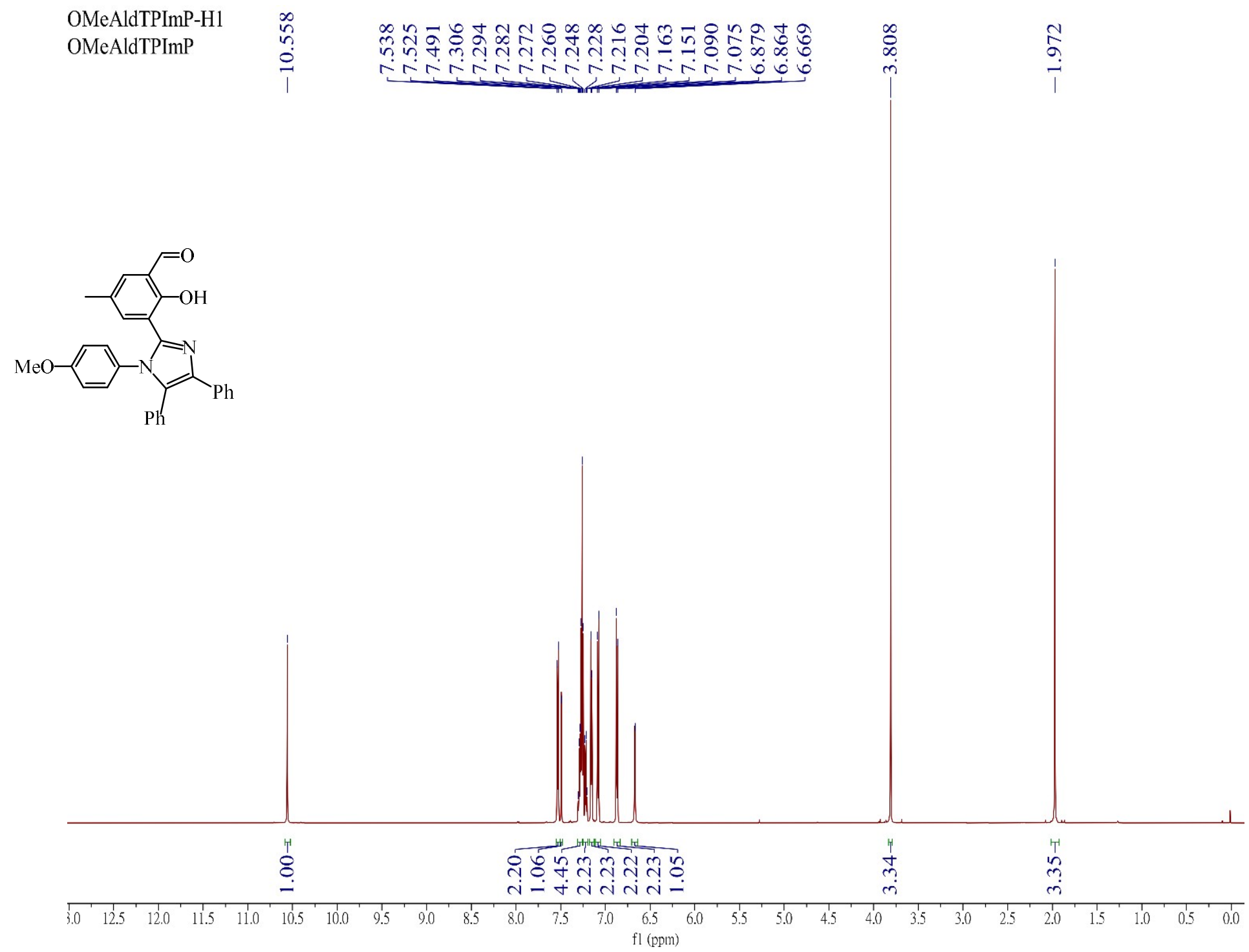

(b)

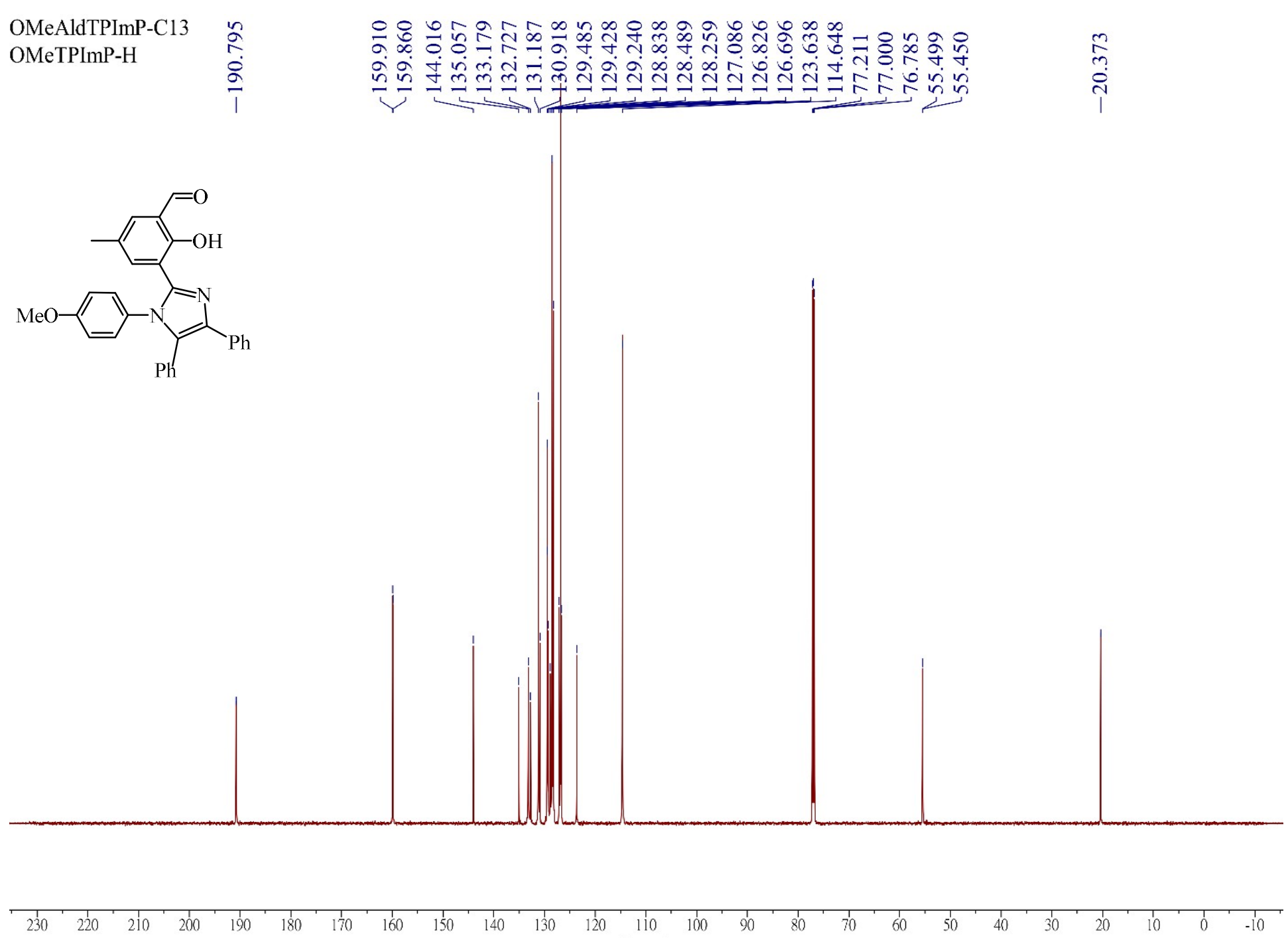

Figure S11. The (a) ^{1}H (600 MHz) and (b) ^{13}C (151 MHz) NMR spectrum of the ^{OMeAld}TPImP-H (in $CDCl_3$).

(a)

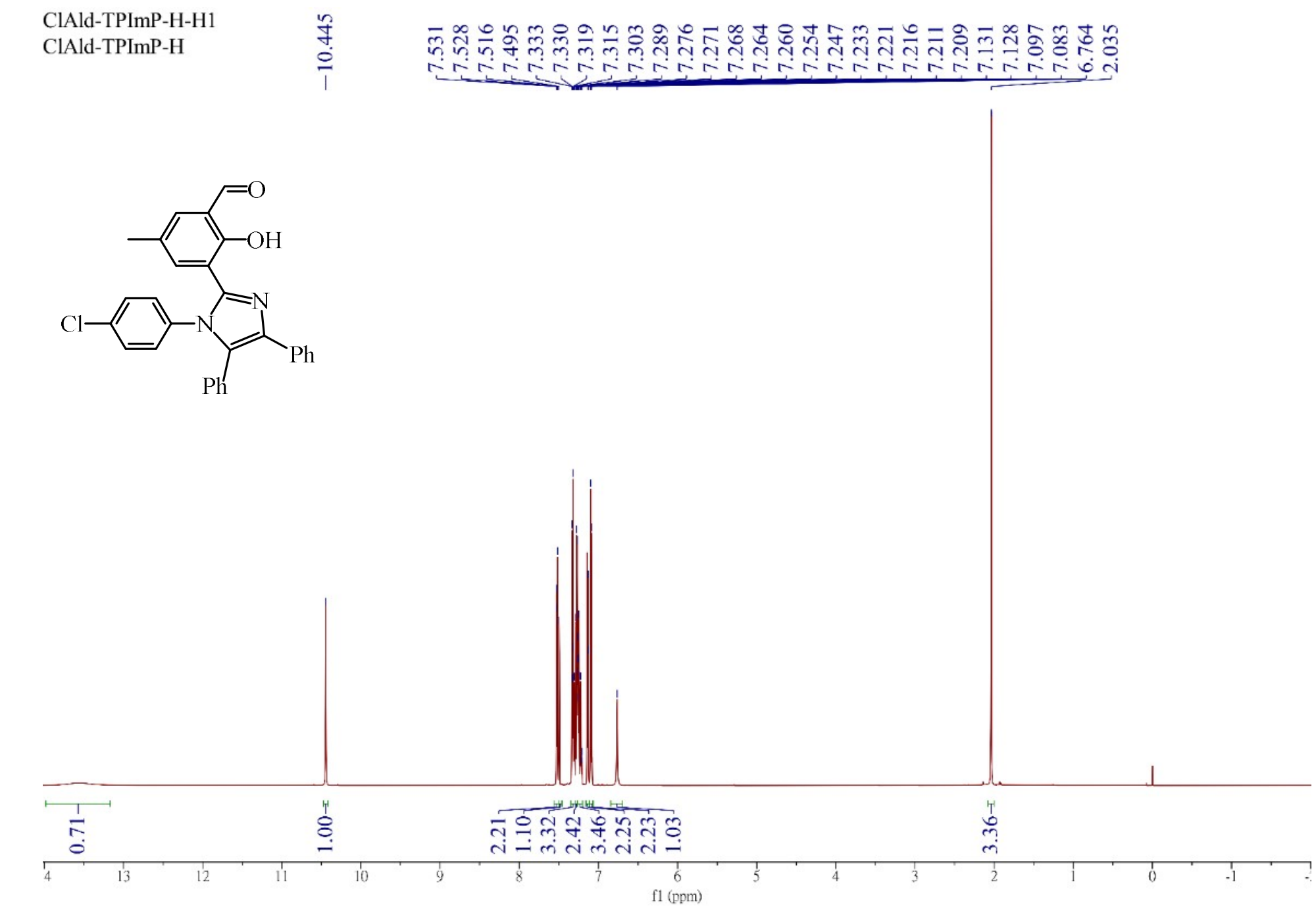

(b)

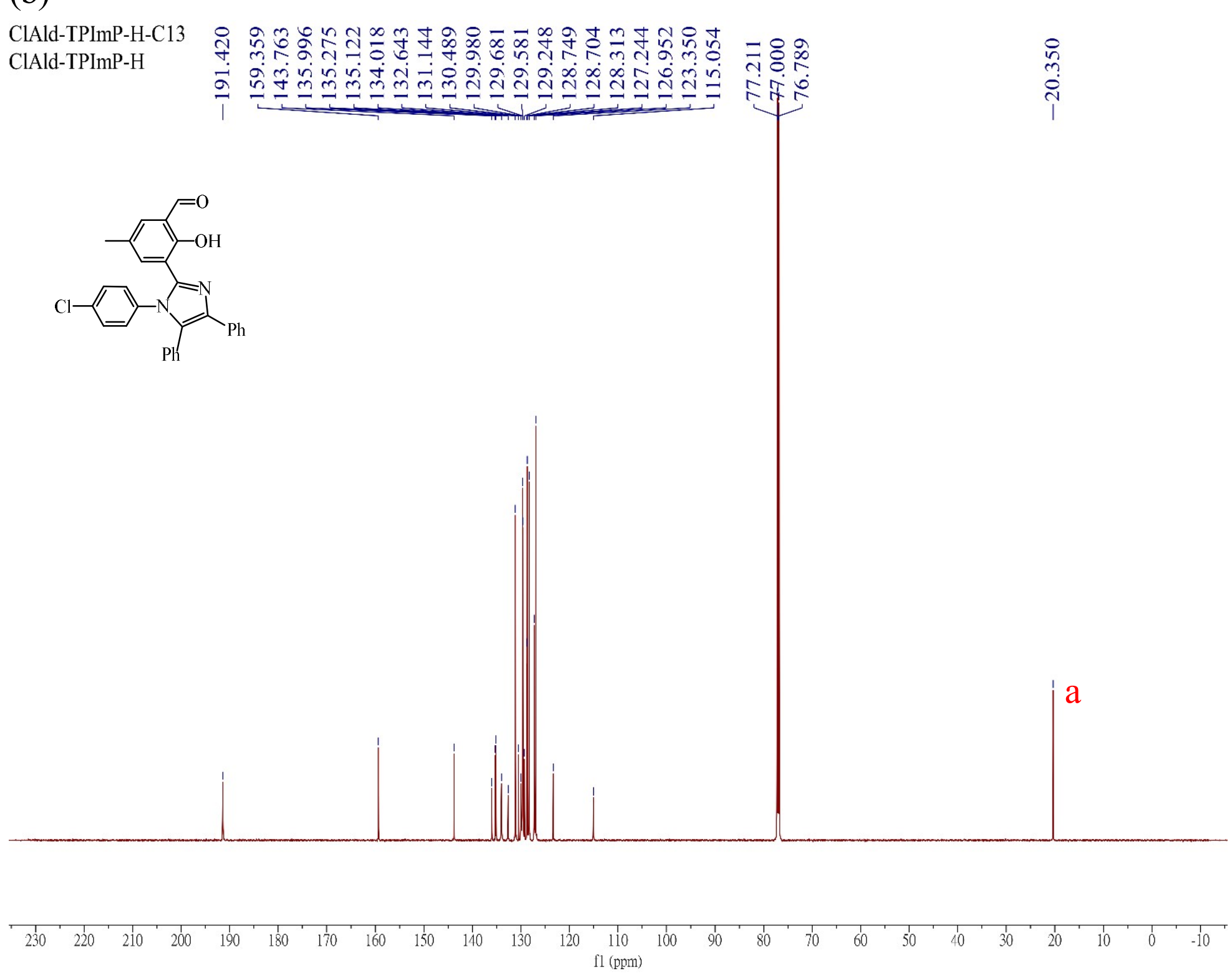

Figure S12. The (a) ^{1}H (600 MHz) and (b) ^{13}C (151 MHz) NMR spectrum of the ^{ClAld}TPImP-H (in $CDCl_3$).

(a)

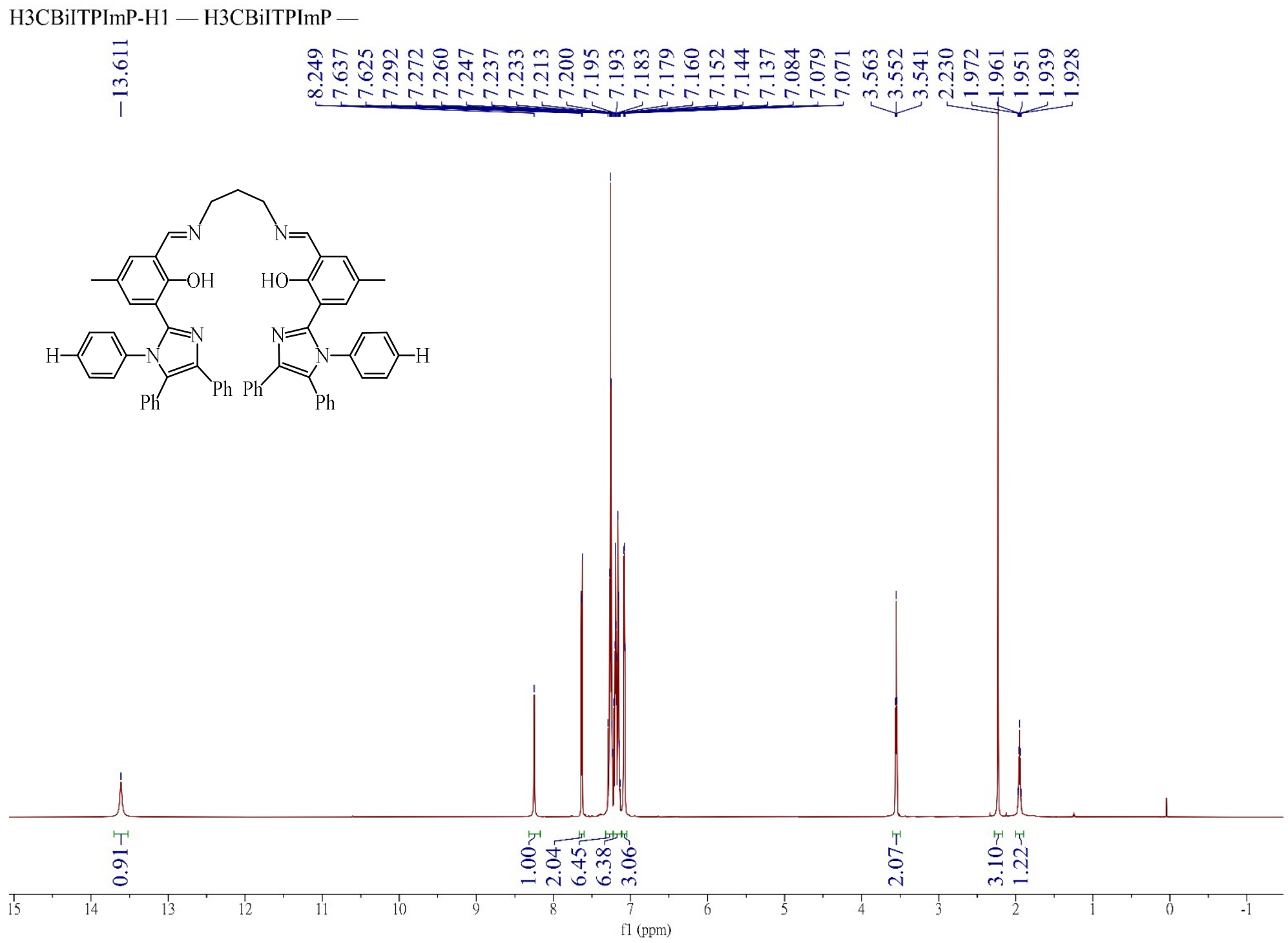

(b)

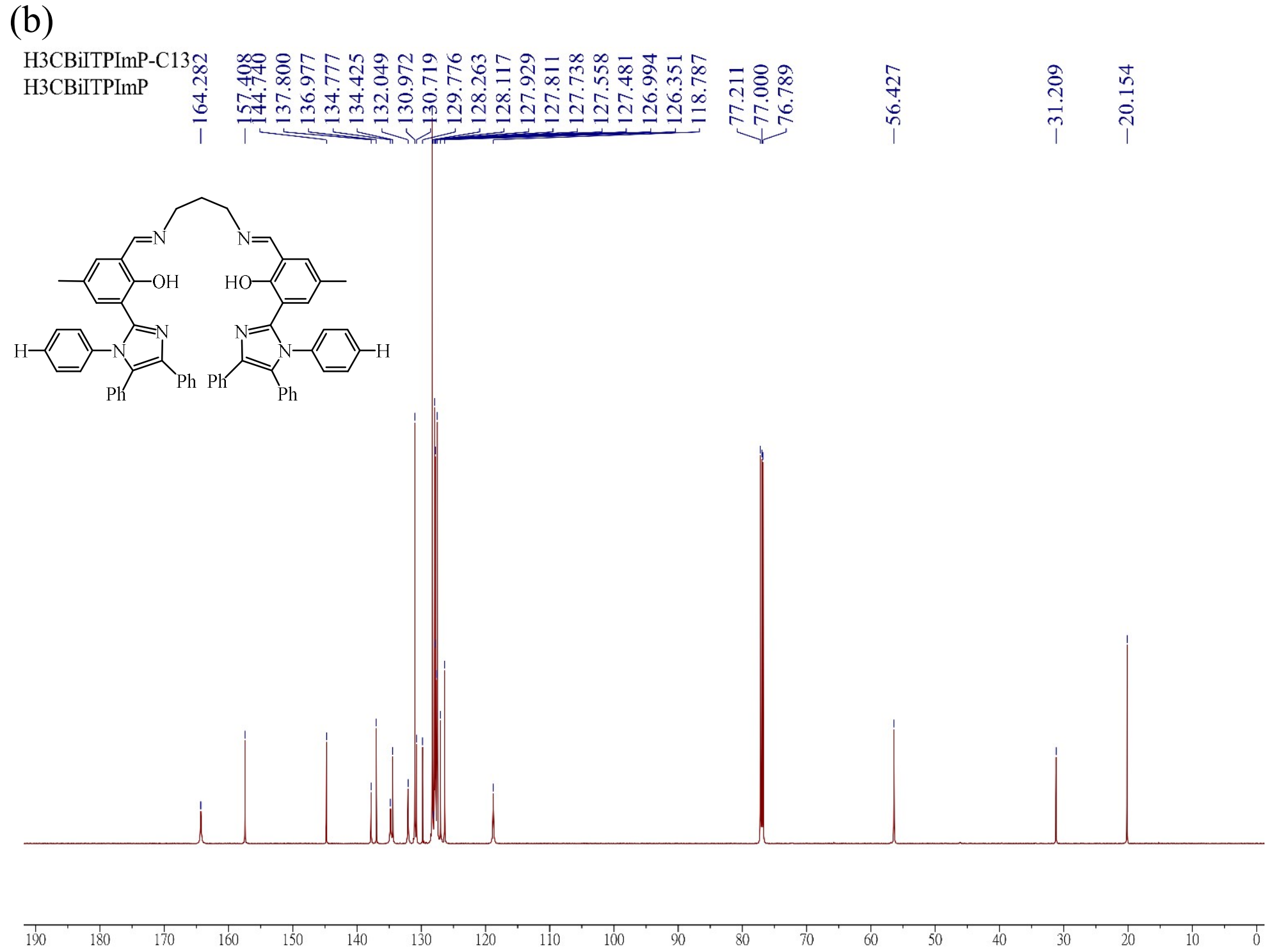

Figure S13. The (a) ^{1}H (600 MHz) and (b) ^{13}C (151 MHz) NMR spectrum of the ^{H3C}L-H_2 (in $CDCl_3$).

(a)

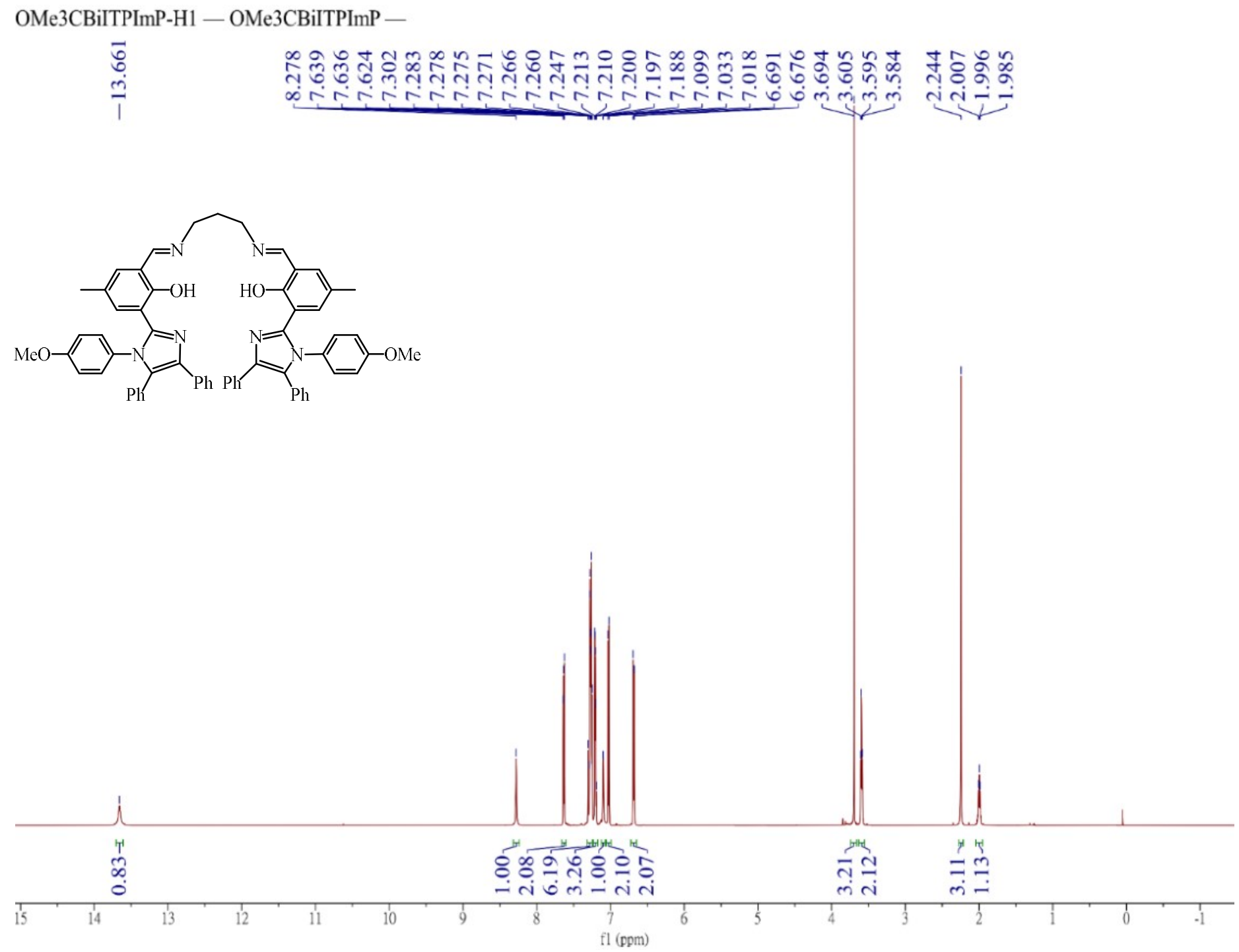

(b)

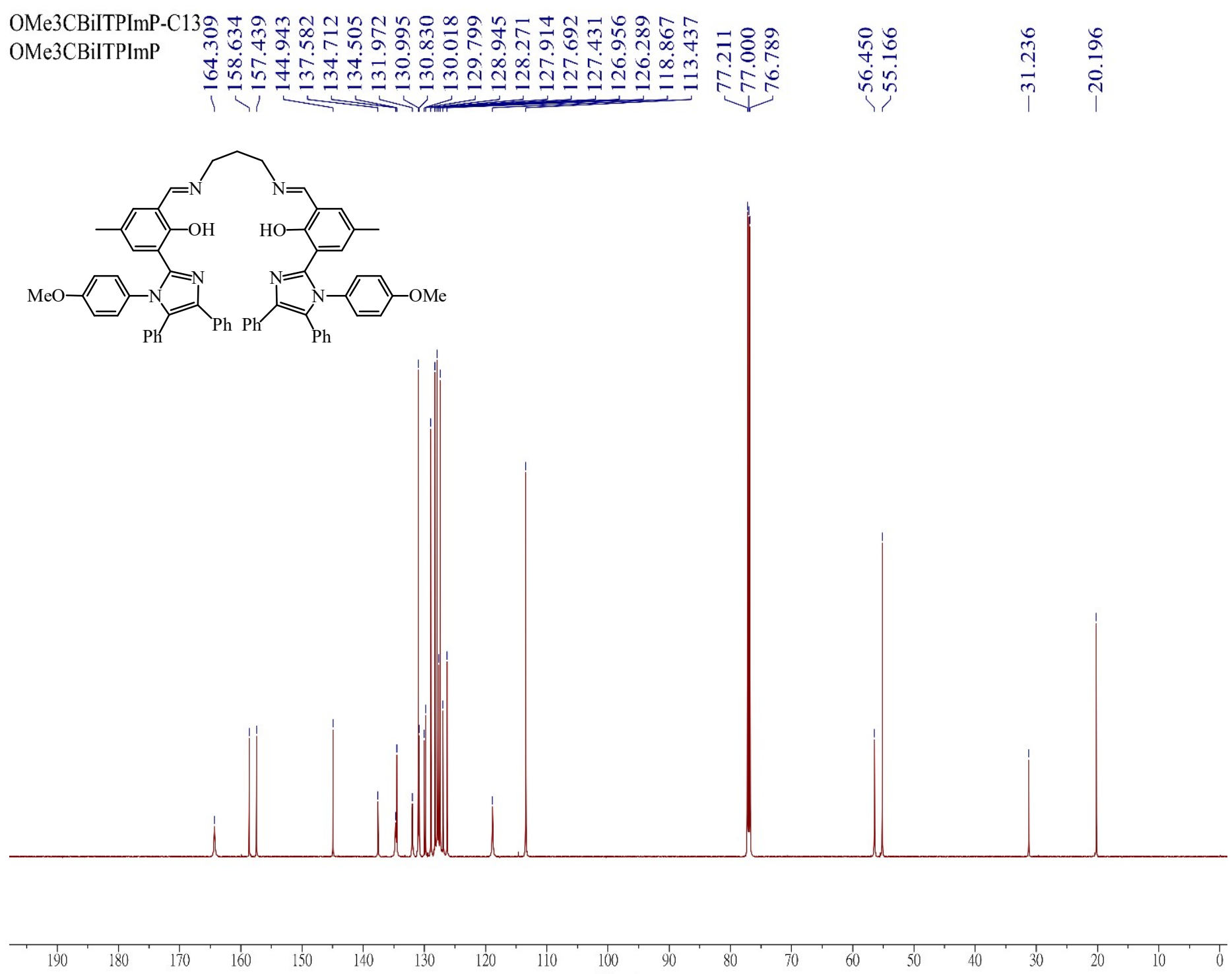

Figure S14. The (a) ^{1}H (600 MHz) and (b) ^{13}C (151 MHz) NMR spectrum of the ^{OMe3C}L-H_2 (in $CDCl_3$).

(a)

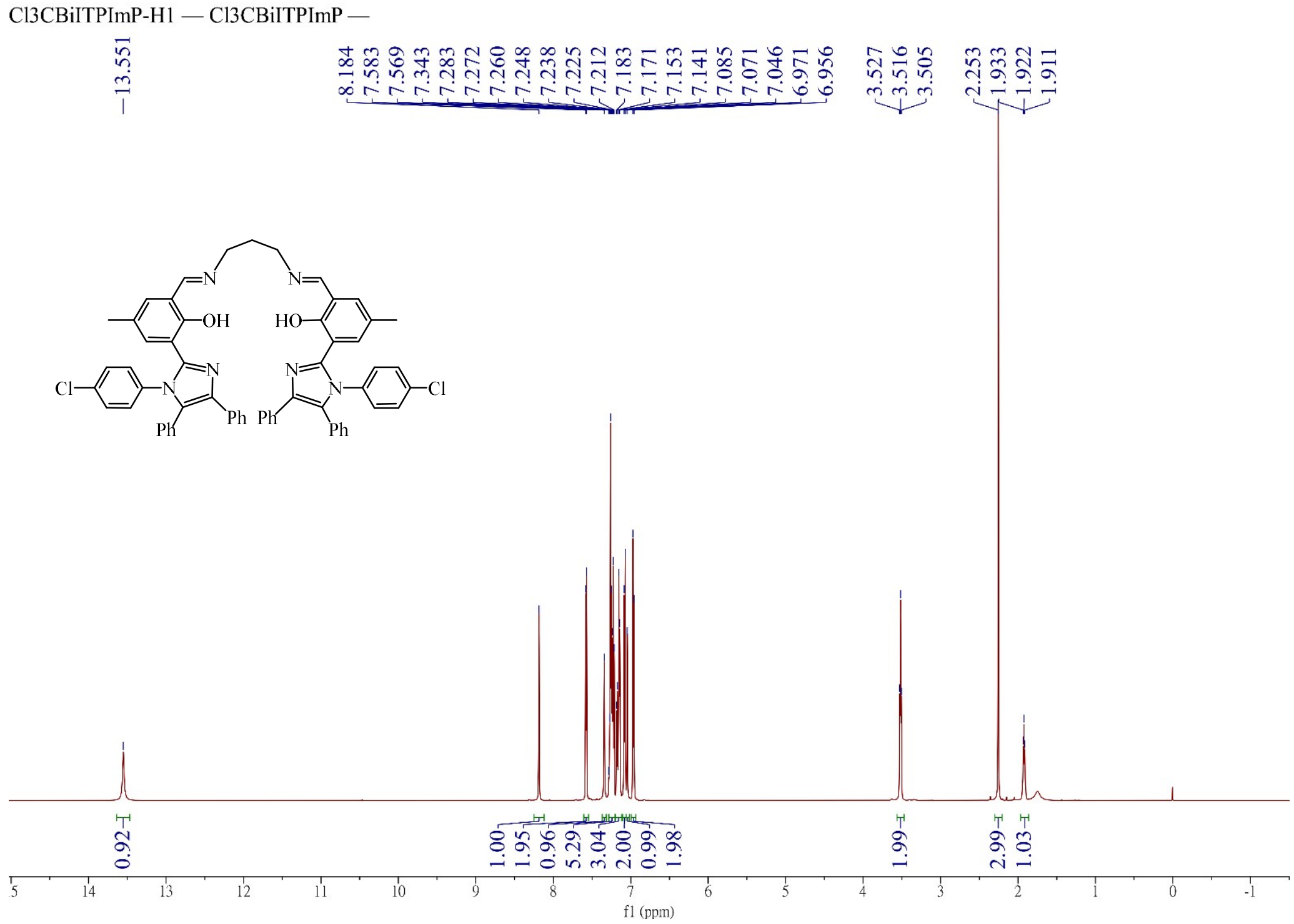

(b)

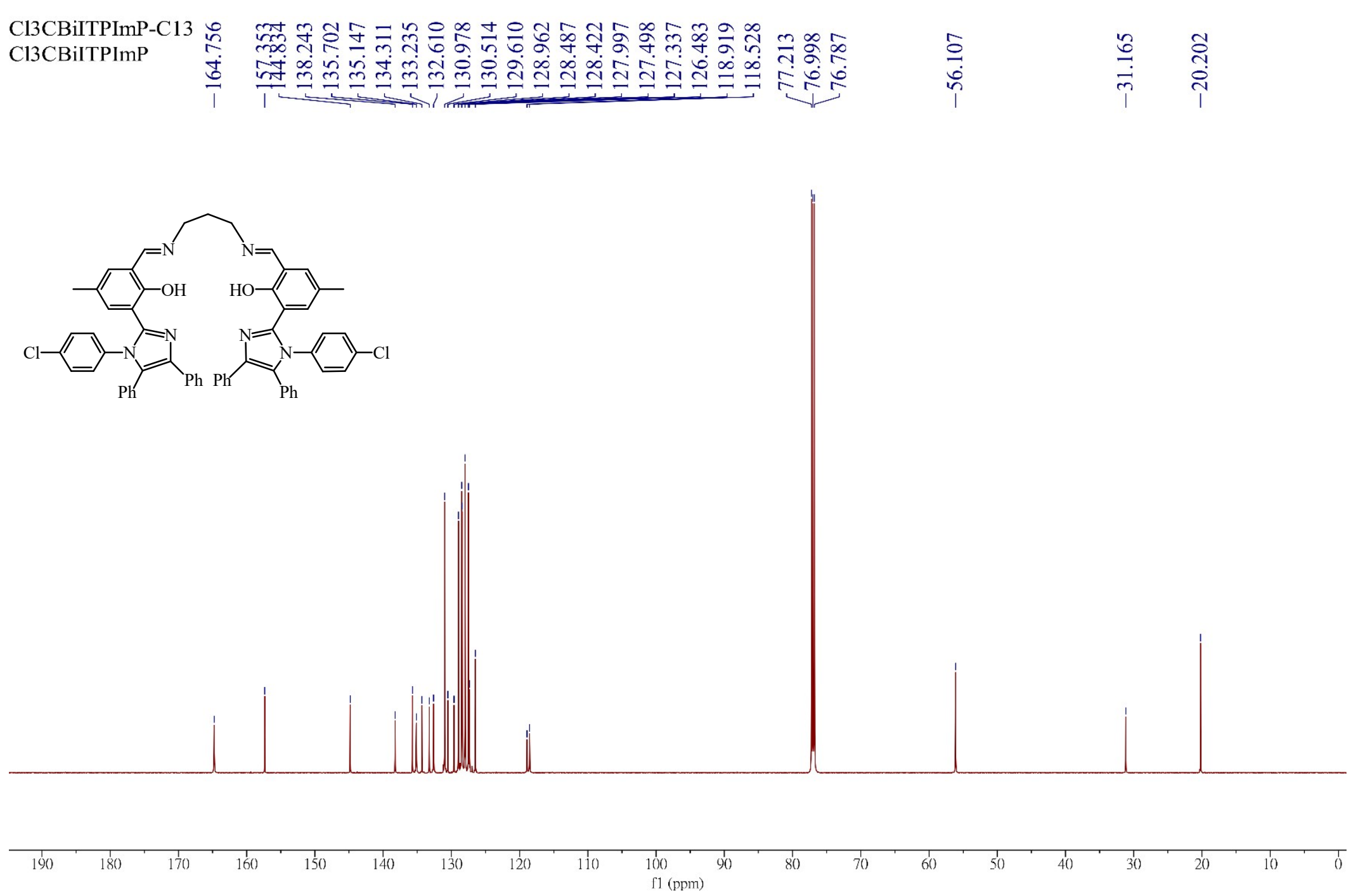

Figure S15. The (a) ^{1}H (600 MHz) and (b) ^{13}C (151 MHz) NMR spectrum of the $^{Cl3C}L\text{-}H_2$ (in $CDCl_3$).

(a)

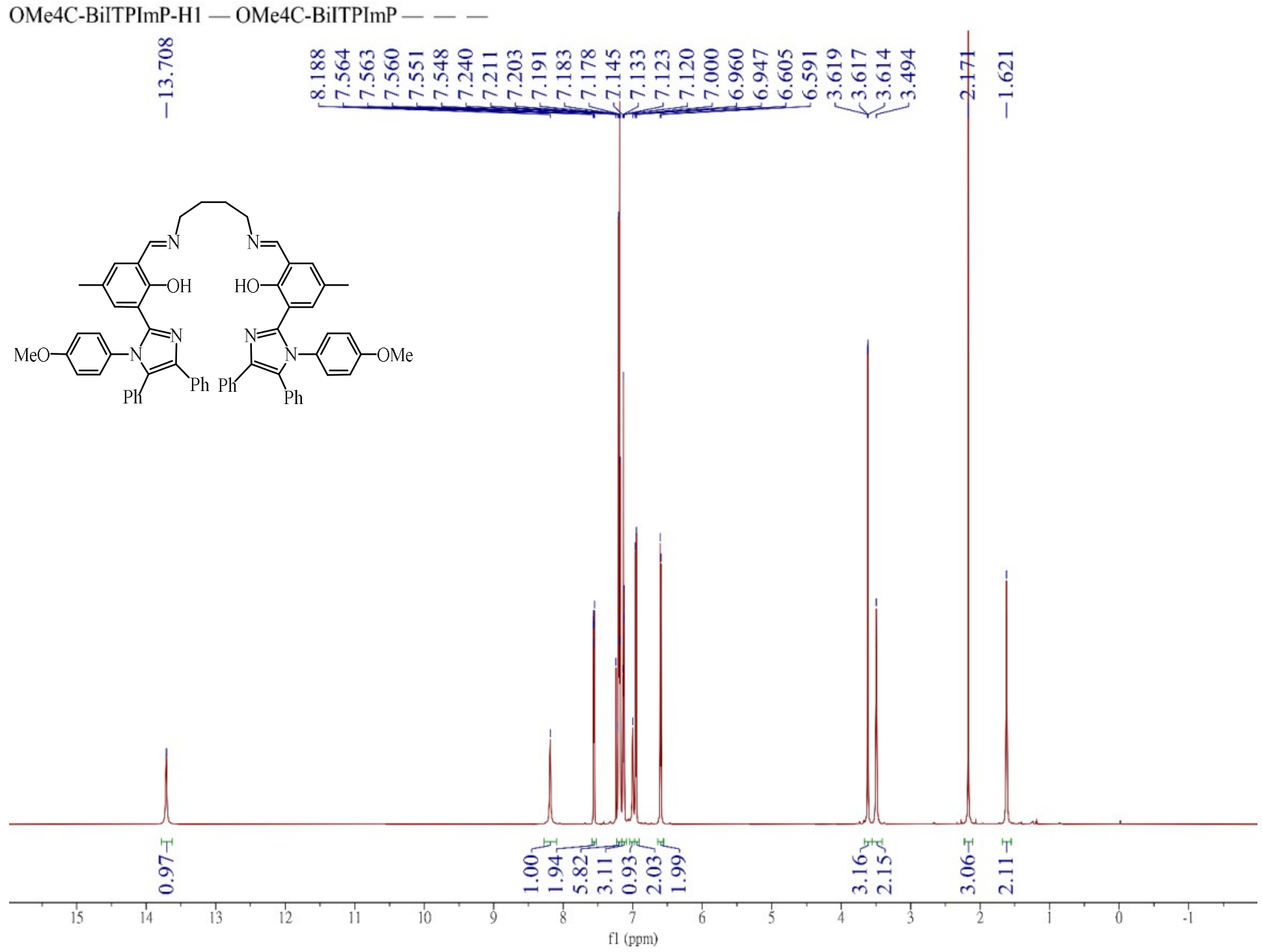

(b)

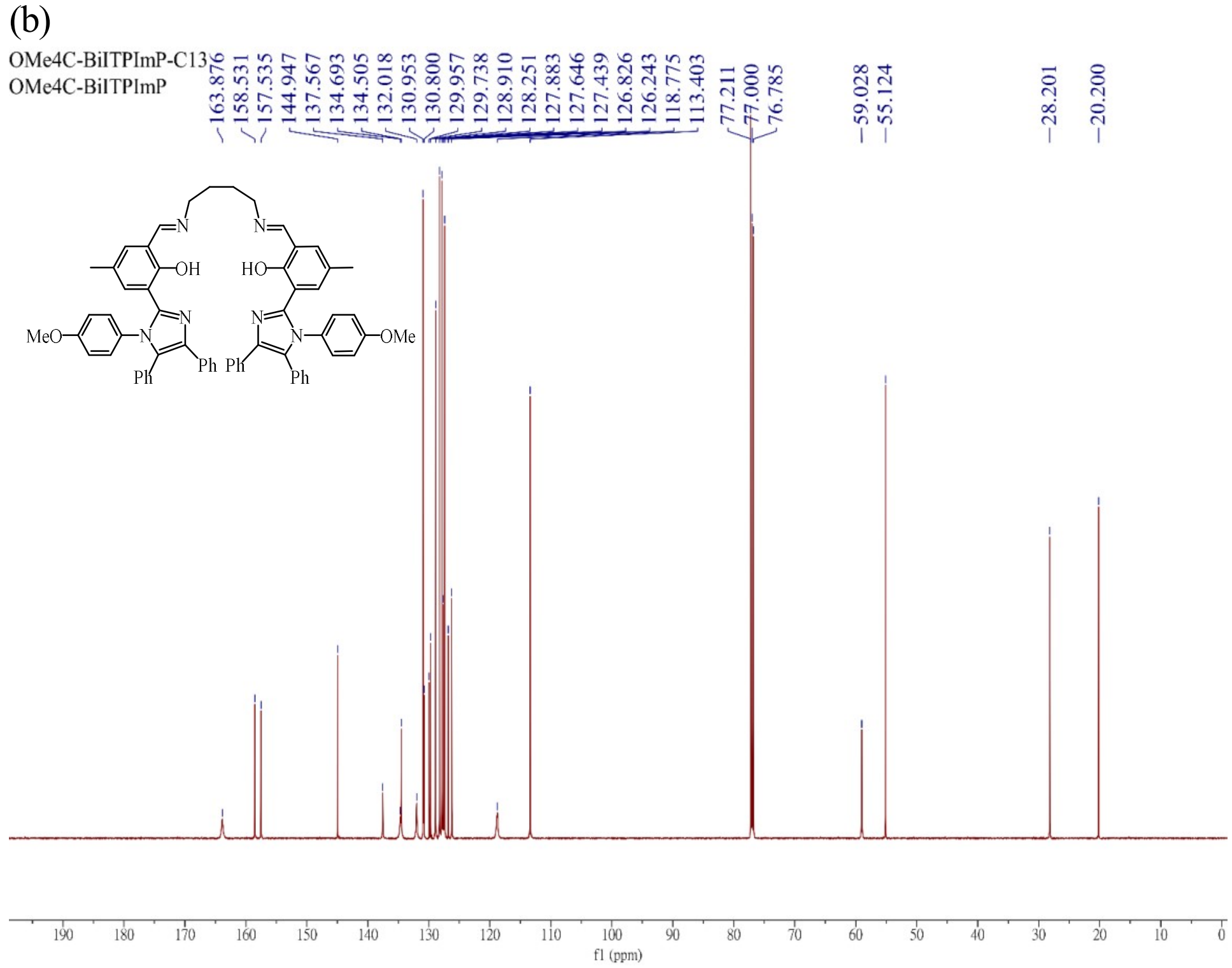

Figure S16. The (a) ^{1}H (600 MHz) and (b) ^{13}C (151 MHz) NMR spectrum of the ^{OMe4C}L-H_2 (in $CDCl_3$).

Table S1. Crystallographic Data of Dinickel Complexes **1**, **3**, **4** and **5'**

	**1**	**3**	**4·CH$_2$Cl$_2$**	**5'·0.5C$_2$H$_5$OH**
Formula	$C_{66}H_{56}Cl_2N_6Ni_2O_6$	$C_{66}H_{54}Cl_4N_6Ni_2O_6$	$C_{69}H_{62}Cl_2N_6Ni_2O_8$	$C_{68}H_{71}Cl_2N_6Ni_2O_{6.5}$
Formula weight	1217.48	1286.37	1291.56	1264.62
Temp (K)	150.0	150.15	150.15	100.15
Crystal system	Triclinic	Monoclinic	Monoclinic	Monoclinic
Space group	P-1	C2/c	C2/c	P2/c
a (Å)	12.1492(12)	15.7826(12)	16.1370(9)	24.4070(3)
b (Å)	12.4461(12)	18.8775(15)	18.7839(10)	10.11280(10)
c (Å)	19.5360(16)	19.5076(12)	19.5975(12)	26.3516(3)
α (deg)	97.258(3)	90	90	90
β (deg)	92.275(3)	94.481(2)	93.546(3)	112.0360(10)
γ (deg)	103.728(4)	90	90	90
V(Å^3)	2839.2(5)	5794.3(7)	5928.9(6)	6029.04(12)
Z	2	4	4	4
D_{calc}(Mg/m^3)	1.424	1.475	1.447	1.393
μ(Mo Kα)(mm^{-1})	0.817	0.894	0.790	2.068
F(000)	1264	2656	2688	2652
Reflections collected	86702	54316	83249	47731
No. of parameters	754	415	401	764
Indep. reflns (R_{int})	11599(0.0544)	5919(0.0453)	6065(0.0505)	11570(0.0291)
$R1[I > 2\sigma(I)]$	0.0788	0.0362	0.0463	0.0406
w$R2$ $[I > 2\sigma(I)]$	0.2086	0.0817	0.1206	0.1064
Goodness-of-fit on F^2	1.111	1.086	1.131	1.028

Table S2. Selected Average Bond Distances and Bond Angles of Dinickel Complexes **1**, **3**, **4** and **5'**

	**1**	**3**	**4·CH_2Cl_2**	**5'·0.5C_2H_5OH**
Ni-N(imidazole)	2.077(5)	2.0727(17)	2.073(2)	2.0221(18)
Ni-N(imine)	2.043(5)	2.034(2)	2.049(3)	2.0156(19)
Ni-O(phenoxy)	2.045(4)	2.0351(16)	2.0395(19)	2.0150(15)
Ni-O(acetate)	2.091(4)	2.0945(16)	2.0971(20)	---
Ni(1)-O(acetate)	2.080(4)	2.0793(16)	2.071(2)	---
Ni(2)-O(acetate)	2.103(4)	2.1151(15)	2.1232(19)	---
Ni-Cl	---	---	---	2.3229(7)
Ni-O(water)	---	---	---	2.1247(16)
Ni-O(ethanol)	---	---	---	2.1290(18)
O(1)-Ni(1)-N(6)	172.85(19)	---	---	170.17(7)
N(5)-Ni(1)-O(2)	174.04(19)	---	---	166.76(7)
O(3)-Ni(1)-O(5)	169.08(16)	---	---	---
O(1)-Ni(1)-N(3A)	---	173.60(7)	175.41(10)	---
O(1A)-Ni(1)-N(3)	---	173.60(7)	175.41(10)	---
O(2)-Ni(1)-O(2A)	---	170.70(9)	170.72(12)	---
O(3)-Ni(1)-O(4)	---	---	---	175.73(7)
O(1)-Ni(2)-N(3)	175.14(17)	---	---	168.96(7)
O(2)-Ni(2)-N(1)	176.31(17)	---	---	126.44(7)
O(4)-Ni(2)-O(6)	158.22(16)	---	---	---
N(1)-Ni(2)-O(1A)	---	175.75(7)	176.38(8)	---
N(1A)-Ni(2)-O(1)	---	175.75(7)	176.38(8)	---
O(3)-Ni(2)-O(3A)	---	157.21(8)	156.54(11)	---
N(1)-Ni(2)-Cl(1)	---	---	---	120.81(6)
O(2)-Ni(2)-Cl(1)				108.68(5)
Ni(1)···Ni(2)	2.8499(10)	2.8450(6)	2.8536(7)	3.0735(5)

Table S3. Copolymerization of CO_2 and CHO Catalyzed by Dinuclear Nickel Complex **6** Under Different Initial CO_2 Pressures for 1 h[a]

Entry	pCO_2^0 (bar)	CHO conv. (%)[b]	% Copolymer[b] (% CO_3 linkages)[c]	TON[d]
S1	13.8	30	>99 (>99)	960
S2	20.7	28	>99 (>99)	896
S3	27.6	26	>99 (>99)	832
S4	34.5	25	>99 (>99)	800

Copolymerization conditions: 100.0 mmol of CHO, $[CHO]_0/[\mathbf{6}]_0$ = 3,200/1, 120 °C. [b]Based on ^1H NMR analysis of the reaction mixture. [c]Based on ^1H NMR determination of the purified copolymers. [d]TON = number of moles of CHO consumed per mole of the catalyst.

Table S4. Kinetic Studies of CHO/CO_2 Copolymerization Mediated by Dinuclear Nickel Complex **6** at Fixed Monomer-to-Catalyst Ratios[a]

Entry	$[\mathbf{6}]_0/[CHO]_0$	Time (h)	CHO conv. (%)[b]	$\ln([CHO]_0/[CHO]_t)$
S1	1/3200	0.5	19	0.2107
S2	1/3200	1	28	0.3285
S3	1/3200	1.25	34	0.4155
S4	1/3200	1.5	40	0.5108
S5	1/3200	2	48	0.6539
S6	1/4000	0.5	14	0.1508
S7	1/4000	1	23	0.2614
S8	1/4000	1.5	32	0.3857
S9	1/4000	2	38	0.4780
S10	1/4000	2.5	44	0.5798
S11	1/5000	1	18	0.1985
S12	1/5000	1.5	24	0.2744
S13	1/5000	2	30	0.3567
S14	1/5000	2.5	36	0.4463
S15	1/5000	3	40	0.5108
S16	1/6000	1	15	0.1625
S17	1/6000	1.5	22	0.2485
S18	1/6000	2	26	0.3011
S19	1/6000	2.5	32	0.3857
S20	1/6000	3	36	0.4463

[a]Copolymerization condition: CHO = 156.25 mmol, pCO_2^0 = 20.7 bar, 120 °C, [b]Based on ^1H NMR analysis of the reaction mixture.

Table S5. Kinetic Parameters of CO_2/CHO Copolymerization Mediated by Dinuclear Nickel Complex **6** at Different CHO-to-Catalyst Ratios

Entry	[**6**]$_0$ (mol%)	Observed rate coefficient, k_{obs} (h^{-1})	ln(k_{obs})	Catalyst concentrations [**6**]$_0$ (mM)	ln[**6**]$_0$
S1	0.03125	0.3024	–1.1960	3.0887	–5.7800
S2	0.02500	0.2149	–1.5376	2.4710	–6.0031
S3	0.02000	0.1593	–1.8370	1.9768	–6.2263
S4	0.01667	0.1409	–1.9597	1.6473	–6.4086

REFERENCES

S1. Sheldrick, G. M. A short history of SHELX. Acta Crystallogr., Sect. A: Found. Crystallogr. 2008, 64, 112–122.

S2. Burnett, M. N.; Johnson, C. K., ORTEPIII, Report ORNL-6895; Oak Ridge National Laboratory: Oak Ridge, TN, USA, 1996.

S3. Spek, A. PLATON SQUEEZE: a tool for the calculation of the disordered solvent contribution to the calculated structure factors. *Acta Crystallogr., Sect. C: Struct. Chem.* **2015**, *71* (1), 9–18.

S4**.** Patiny, L.; Borel, A. ChemCalc: A Building Block for Tomorrow's Chemical Infrastructure. *J. Chem. Inf. Model.* **2013**, *53* (5), 1223–1228.
